# Planar Differential Growth Rates Initiate Precise Fold Positions in Complex Epithelia

**DOI:** 10.1016/j.devcel.2019.09.009

**Published:** 2019-11-04

**Authors:** Melda Tozluoǧlu, Maria Duda, Natalie J. Kirkland, Ricardo Barrientos, Jemima J. Burden, José J. Muñoz, Yanlan Mao

**Affiliations:** 1 MRC Laboratory for Molecular Cell Biology, University College London, London WC1E 6BT, UK; 2 Mathematical and Computational Modeling (LaCàN), Universitat Politècnica de Catalunya, Barcelona, Spain; 3 Institute for the Physics of Living Systems, University College London, London WC1E 6BT, UK; 4 College of Information and Control, Nanjing University of Information Science and Technology, Nanjing, Jiangsu 210044, China

**Keywords:** computational modeling, tissue mechanics, finite element, folding, morphogenesis

## Abstract

Tissue folding is a fundamental process that shapes epithelia into complex 3D organs. The initial positioning of folds is the foundation for the emergence of correct tissue morphology. Mechanisms forming individual folds have been studied, but the precise positioning of folds in complex, multi-folded epithelia is less well-understood. We present a computational model of morphogenesis, encompassing local differential growth and tissue mechanics, to investigate tissue fold positioning. We use the *Drosophila* wing disc as our model system and show that there is spatial-temporal heterogeneity in its planar growth rates. This differential growth, especially at the early stages of development, is the main driver for fold positioning. Increased apical layer stiffness and confinement by the basement membrane drive fold formation but influence positioning to a lesser degree. The model successfully predicts the *in vivo* morphology of overgrowth clones and *wingless* mutants via perturbations solely on planar differential growth *in silico*.

## Highlights

• *Drosophila* wing discs grow with spatial and temporal heterogeneity
• This differential growth determines the positions of epithelial folds
• Constriction from the basement membrane is necessary for correct fold initiation
• Our computational model correctly predicts the shape of growth mutants

## Graphical Abstract

**Figure:**
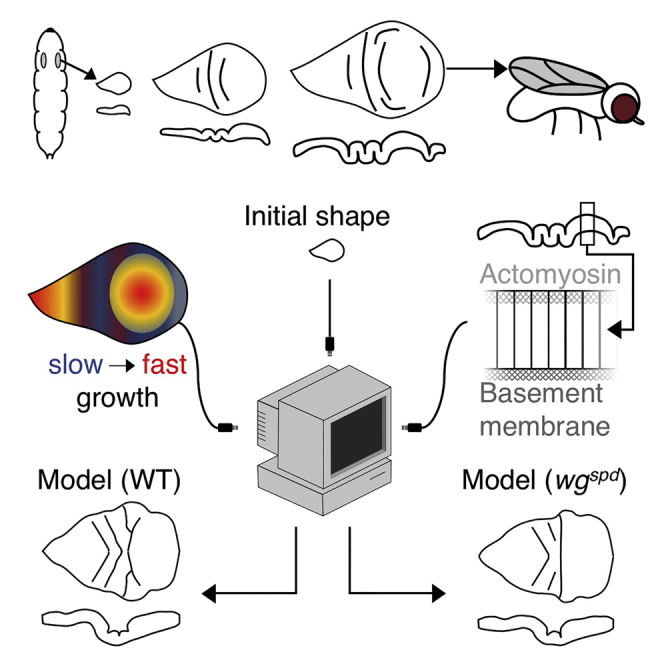

## Teaser

Like an origami crane forming from the folds of a paper, the precise positioning of multiple folds in epithelia is key for the final shape of a tissue. Tozluoǧlu et al. present a computational model that explains how precise fold positioning can be predicted by heterogeneous growth in epithelia.

## Introduction

Epithelial folding is a fundamental morphological process that is encountered abundantly during the development of multiple organisms. It is used to sculpt organs from flat epithelial sheets into complex structures such as tubular, undulated, and branched tissues (Nelson, 2016). Folds may function as a means of compartmentalization, surface area increase to facilitate material exchange, or may emerge as a side effect of pathology, such as overgrowth in cancer (Gutzman et al., 2008, Hruban et al., 2000, Nelson, 2016).

Possibly the most extensively studied driver of folding is apical constriction via accumulation of non-muscle myosin II (Dawes-Hoang et al., 2005, Granholm and Baker, 1970, Lecuit and Lenne, 2007, Lewis, 1947, Polyakov et al., 2014). Basal relaxation, lateral constriction (Štorgel et al., 2016, Sui et al., 2012, Sui et al., 2018, Wang et al., 2016, Wen et al., 2017) and cell shortening (Conte et al., 2012, Gutzman et al., 2008, Sherrard et al., 2010), constriction of the regions surrounding the prospective fold (Kondo and Hayashi, 2013, Monier et al., 2015, Röper, 2012), and constriction of supporting structures by other cells (Hughes et al., 2018), have all been demonstrated as potential folding mechanisms. Other force generation mechanisms, such as cell rounding in mitosis, adhesion shifts, or basal extrusions can also induce folds (Kondo and Hayashi, 2013, Kondo and Hayashi, 2015, Monier et al., 2015, Wang et al., 2012). In all these scenarios, what defines the position of the prospective fold is a biochemical signaling mechanism responsible for selecting the cell population to actively generate the forces.

Beyond cellular forces, differential growth between tissue layers can induce patterns of buckling such as those of the gut (Savin et al., 2011), dental epithelium (Marin-Riera et al., 2018), brain (Tallinen et al., 2014), lungs (Kim et al., 2015), and the rippled edges of plant leaves (Dervaux and Ben Amar, 2011, Liang and Mahadevan, 2009, Marder et al., 2003). External structures, such as the extracellular matrix (ECM), can provide sufficient confinement to growing tissue to induce folding (Diaz-de-la-Loza et al., 2018). Uniform growth and constriction will induce folds in predictable patterns following the physical rules of buckling (Karzbrun et al., 2018, Pocivavsek et al., 2008, Shyer et al., 2013, Wang and Zhao, 2015). The patterns can then be refined by the overall shape of the tissue (Tallinen et al., 2016) or further local perturbations such as constriction by smooth muscles (Kim et al., 2015), adhesive forces, and ECM alterations (Sui et al., 2012).

While shaping a tissue, various mechanisms are likely to occur in parallel, such that once a fold is initiated in a selected position, a combination of modifications of the confinement and active force generation can help its progression. A key question is, how are the initial positions of the folds defined to achieve the precise tissue morphology (Nelson, 2016)?

As an emergent mechanical phenomenon, fold position selection is likely to depend on a combination of the forces accumulating in the growing tissue, the dynamics of surrounding structures, and the inherent properties of the tissue such as stiffness or shape prior to folding. None of these factors are trivial to investigate independently in an experimental system—how would one eliminate the influence of the shape of a tissue on its form? Therefore, the topology of folding morphogenesis is a problem particularly suitable for computational exploration.

**Figure 1:**
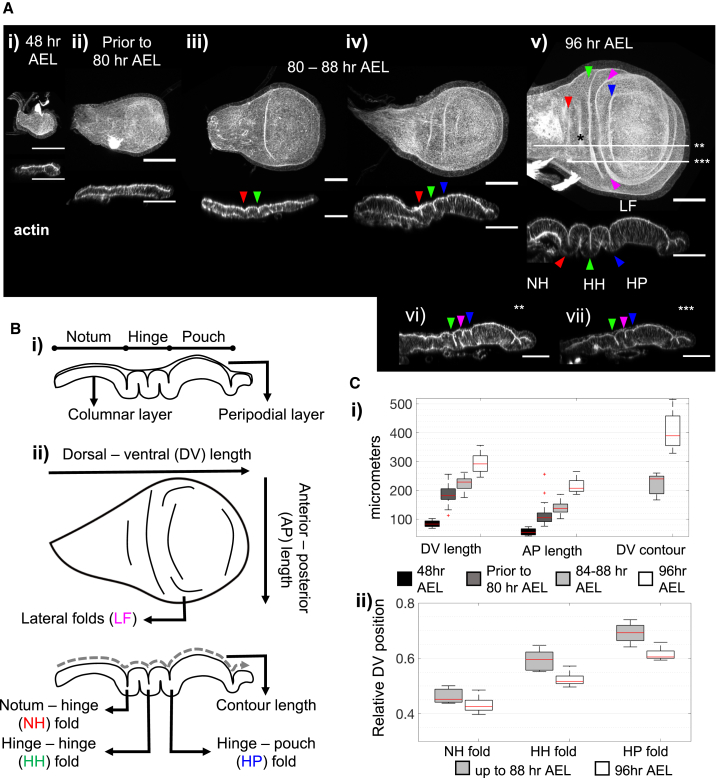
Characterization of Wing Imaginal Disc Morphology (A) (i–v) The morphology changes between 48 and 96 h AEL. Maximum projection images, top and cross-section from DV axis midline views. Arrowheads point to HN, HH, HP, and LF in red, green, blue, and magenta, respectively. Scale bars are 50 μm. Due to the projection, basal folds are visible on the top view, example marked by black star on (v). (vi and vii) Lateral cross-sections along lines marked with white stars on (v). (B) Schematic of the wing disc structure. (i) Domains are labeled, the thin peripodial layer is hardly visible on the experimental images. (ii) Top and cross-section with developmental axes and fold names labeled. (C) (i) Wing disc size during fold formation, developmental age progresses from black to white, see STAR Methods for n. At 48 h AEL, the mean AP and DV lengths are 56 and 84 μm, respectively. Prior to 80 h AEL, 114 and 185 μm; at 88 h AEL, 128 and 222 μm. At 96 h AEL, 214 and 294 μm, the apical contour length on the DV axis is 402 μm. (ii) Fold positions normalized to DV length, error bars represent one standard deviation. Up to 88 h AEL, the positions are 0.48, 0.58, and 0.66 for NH, HH, and HP folds respectively. At 96 h AEL, they are 0.43, 0.52, and 0.61. 72–88 h AEL, NH fold n = 22, HH fold n = 29, HP fold n = 18. 96 h AEL, n = 14 for all folds. Boxes represent 25^th^ and 75^th^ percentiles, medians in red, whiskers extend the most extreme data points that are not outliers, outliers plotted with red plus.

*Drosophila melanogaster* is an established model system for studying morphogenesis. The wing imaginal disc of *Drosophila* forms three distinct folds, perpendicular to the dorsal-ventral axis. These major folds are highly reproducible in their number and positions, marking the boundaries between the notum, hinge, and pouch regions of the wing disc (Figure 1). There is evidence that basal relaxation, lateral constriction, and stiffness changes within the cell compartments play roles in the generation of the folds (Sui et al., 2012, Sui et al., 2018, Wang et al., 2016). However, what determines their positions and drives the initiation of these folds is an open question. This makes the wing disc an ideal experimental system to investigate general mechanisms that control the position of folds in complex epithelia, a problem that has been under-investigated but critical in determining the final functional architecture of the tissue.

Here, we investigate the minimum set of requirements for initiating the correct topology of the complex multi-folded wing disc epithelia. Our search allows us to postulate planar differential growth as a mechanism for fold initiation. We measure the differential planar growth rates of wing imaginal disc development with high spatial resolution. Utilizing a computational approach, and from experimental measurements, we demonstrate that the differential growth in the plane of the tissue, especially in the early stages, under the compression of the ECM, drives the initiation of three folds from the apical surface. When challenged with mutants, our model can successfully recapitulate the morphology of overgrowth clones. We also predict that a reduction of early growth in the hinge region, prior to any folds being visible, can affect the number and position of folds that form later. We experimentally validate this prediction against a *wingless* mutant, which has reduced proliferation specifically in the hinge region (Neumann and Cohen, 1996). Our simulations show that the alterations of planar growth rates are sufficient to explain the observed fold perturbations in overgrowth and undergrowth mutants.

## Results

### Characterization of Wing Imaginal Disc Fold Morphology

The wing imaginal disc is a monolayered epithelial sac. The peripodial layer, positioned as the top layer throughout this paper, is formed of squamous cells. The bottom layer is the columnar layer, which forms the folds by the end of third instar (Figures 1Aiv, 1Av, and 1B). The apical surfaces of both layers face each other toward the lumen, and the basal surfaces face outwards. Both apical and basal surfaces harbor ECM of different compositions (Pastor-Pareja and Xu, 2011, Ray et al., 2015).

We focus our attention on the columnar layer and characterize the two-day period from 48 h AEL, when the tissue is flat (Figure 1Ai), to 96 h AEL, when the columnar layer has formed three folds at the hinge region (Figures 1Av and 1B). From dorsal to ventral tips, these folds are termed notum-hinge (NH), hinge-hinge (HH), and hinge-pouch (HP) folds (Figure 1Bii). Between the HH and HP folds, the tissue forms additional lateral folds (LF) that do not reach the midline (Figures 1Avi, 1Avii, and 1Bii). There are multiple smaller folds at the ventral tip of the wing disc, where the tissue loops connect the columnar layer to the peripodial layer. These smaller folds are beyond the scope of the current work, as the largely unknown dynamics of the peripodial layer are not included in the current model.

In our analysis, we segment the development into three morphological stages (Figures 1A and S3A): (1) The early stage before initiation of folds, ending prior to approximately 80 h AEL. The tissue is relatively flat, and folds do not start forming (Figures 1Aii and 1Ci). (2) The intermediate stage where the folds are starting to initiate on the apical surface, with the possibility that the HH fold has fully formed, ending by 88 h AEL (Figures 1Aiii, 1Aiv, and 1Ci). (3) The stage where all folds are established, ending approximately 96 h AEL (Figures 1Av and 1Avi). The folds are formed at reproducible, precise positions as normalized to the tissue DV length (Figure 1Cii).

We match the initial state of our simulations to the tissue size and shape at 48 h AEL and start simulations using simple growth rates derived from the changes in dimensions of the wing disc as described above (Figure 1Ci). Step by step, we add in external confinement with apical ECM and BM, physical property heterogeneities, and fine growth patterns to characterize the requirements of fold initiation in the wing disc.

### The Computational Model

For the purposes of identifying the mechanisms driving wing disc folding, we develop a finite element (FE) model (Bonet and Wood, 2008) of tissue morphogenesis. The goal of this model is to realistically capture the mechanical behavior of the tissue, then simulate its shape dynamics for different tissue physical properties and growth scenarios. The dynamics of the tissue emerge by balancing the elastic forces upon deformation and viscous resistances against the shape changes at each time step.

**Figure 2:**
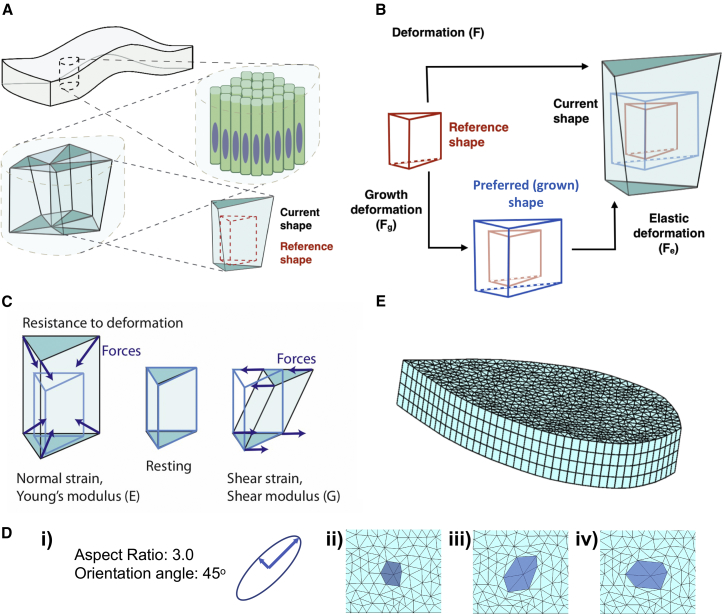
The Computational Model Design (A) Schematic describing the finite elements. (B) Schematic of growth methodology in the model, red wire plot, the reference shape of the element; blue wire plot, the preferred shape of the element. (C) Elastic forces generated by elements upon deformation. (D) Simulation of clone (dark blue) growth surrounded by non-growing tissue (i) the oriented growth input (ii) Initial state of a tissue fragment, (iii) after application of growth in (i). (iv) Tissue in (ii) after growing with the same rates at an orientation angle of zero degrees. (E) Initial mesh of the simulations, at 48 h AEL. See also Figure S1.

In our model, the tissue is defined as a non-homogeneous continuous material. It is composed of a set of elastic FEs in the shape of triangular prisms. These prism elements are independent of the cells in the tissue and can have sizes varying from subcellular to multicellular scales (Figures 2A–2C and S1A). These elements have fixed “reference shapes,” which represent the initial shape of the element (Figure 2B, red wireframe). The growth is defined in the form of a deformation relative to the element’s reference shape. The shape resulting from the combination of growth and the reference shape becomes the element’s current grown or “preferred” shape (Figure 2B, blue wireframe). The total deformation of each element is calculated with respect to its reference shape at each time step. This total deformation is then decomposed into two components, the expected deformation due to growth of the element and the remaining elastic deformation (Rodriguez et al., 1994, Taber, 1995) (Figure 2B). Elements resist the elastic deformation with reactive forces (Figure 2C). The dynamics of morphogenesis then is governed by the forces depending on the viscoelastic properties and growth of the tissue. The relationship between the elastic deformations and resistance forces is modeled as a Neo-Hookean material, and a viscous resistance from the environment is applied on the exposed surfaces. In our approach, we extend the existing decomposition methodology to include oriented growth that follows the plane of the tissue (Figures 2D and S1B). In addition to active growth, the model allows for remodeling of tissue sub-compartments, such as the BM. In remodeling, the growth of an element is slowly updated such that the preferred shape slowly approaches to its current (deformed) shape, relaxing the tissue strains in the process.

The model starts with the preferred and current shapes of each element being equal to the element’s reference shape. As the tissue grows in time, a mismatch between the preferred and current shape emerges, creating elastic deformations and generating consequent reactive forces. Balancing these elastic forces against deformation with the viscous resistance to movement at each time step, the new current shape of each element, therefore the whole tissue is obtained.

Further details of the modeling procedures, meshing (Shewchuk, 2005), boundary conditions (Muñoz and Jelenić, 2004), linearization (Hughes, 2000), a pseudo code of the simulations and a table summarizing all utilized model parameters can be found in Methods S1, Methodology for the Computational Model.

### Resistances from Apical-Basal Surface Confinement Are Essential for Folding

**Figure 3:**
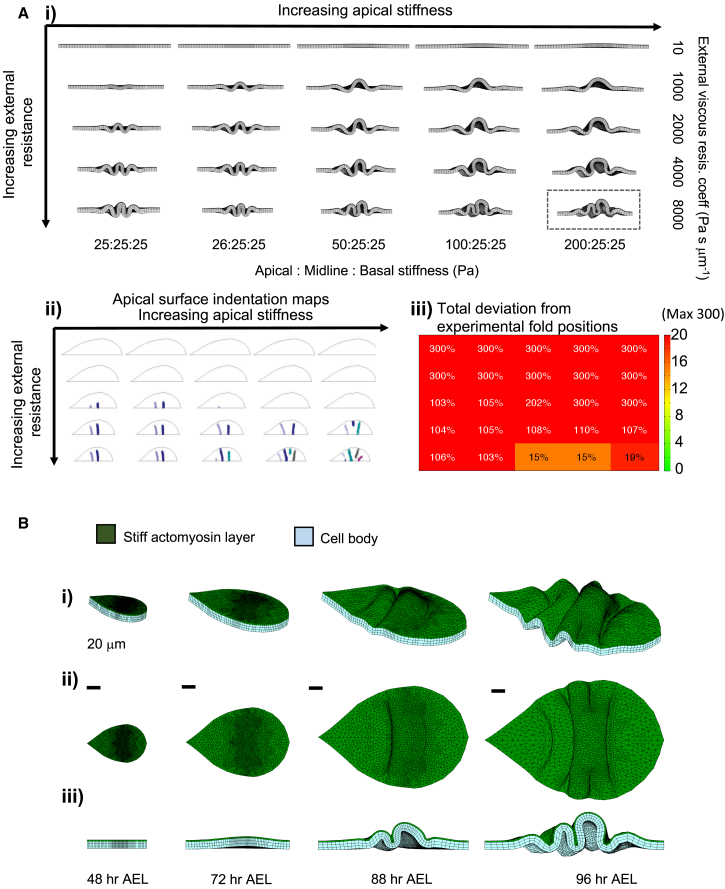
Relative Increase in Apical Stiffness and External Resistance to Tissue Growth Are Essential for Fold Formation (A) (i) Simulations from 48 to 96 h AEL, with uniform planar growth rates. Images show the cross-section from the DV axis midline at 96 h AEL, ventral tip on the right. Columns, increasing external viscous resistance; rows, increasing apical stiffness relative to the rest of the tissue. Poisson ratio is taken to be 0.29 for all simulations (Schluck et al., 2013). (ii) Apical indentation maps. (iii) Fold position deviation scores. The grid organization is same in (i–iii). (B) Simulation boxed in (Ai). (i) Orthogonal perspective view, (ii) top view, (iii) cross-section from the DV axis midline. Scale bars are 20 μm. See Video S1 and Figure S2.

We start our simulations with uniform, constant growth rates that would bring the tissue AP and DV contour lengths from their sizes at 48 to 96 h AEL, in a 48-h time window (0.028 h^−1^ in AP and 0.033 h^−1^ in DV). Simulations with uniform growth on a tissue with minimal external resistance and homogenous physical properties do not form any folds (Figure 3A, top-left corner). This result reinforces that the tissue must have external factors driving compression.

During morphogenesis, both apical ECM and BM can exert resistances to tissue growth and movement and contribute to correct tissue architecture (Diaz-de-la-Loza et al., 2018, Hannezo et al., 2015). Initially, we represent this as a viscous external resistance on the apical and basal surfaces. As the viscous resistance is increased, the tissue starts forming ripples with compact, regular folds, initiating from the center and distributing toward the dorsal and ventral edges (Figure 3Ai, first column). To assess the fold initiation, we generate apical indentation maps (Figure 3Aii), then calculate the total deviation in fold positions as a percentage of the DV length, each missing fold deviation contributing 100% to the sum (Figure 3Aiii). Within the tested range with uniform tissue physical properties (Figures 3Ai–3Aiii, first columns), the tissue can form only two folds. The peaks of these folds are also higher than the apical surface of the pouch and notum regions, which is not the case for the wing disc.

Next, we consider physical property heterogeneities as a source of breaking symmetry. It is likely that the imaginal disc epithelium has heterogeneities, especially along its apical-basal axis. The dense actomyosin mesh on the apical surface, the actin of the basal surface, or the accumulation of the cell nuclei in the middle zone could all result in higher stiffness than the rest of the cell (Farhadifar et al., 2007, Meyer et al., 2011, Sui et al., 2018). To account for all these possibilities, we simulate wing disc growth with an increased stiffness on the apical surface (Figure 3A), on apical and basal surfaces (Figure S2A), or on the midline (Figure S2B). Of the tested cases, only stiffness increase on the apical surface can generate three folds; with 15%–19% deviation from the correct fold positions at tissue midline (Figures 3Ai–3Aiii bottom rows, B; Video S1). However, none of the cases generate folds similar to the experimental morphology (Figure 1Av).

Video S1. Uniform Planar Growth Rates Are Not Sufficient to Generate Experimental Fold Morphology, Related to Figure 3 Tissue growing from 48 to 96-h AEL, with uniform in-plane growth rates. The growth rate is 0.033 h-1 in DV, and 0.028 h-1 in AP, as calculated from Figure 1C. Apical stiffness is 200 Pa (green), and stiffness for the rest of the cell body is 25 Pa (blue). Apical ECM or BM effects modeled as viscous resistances and external viscous resistance coefficient applied to both surfaces at 8,000 Pa s μm^−1^. Scale bar is 20 μm. Simulation time depicted on the frames.

With this analysis, we conclude that external resistance to growth is essential for buckling the tissue and that increased apical stiffness can induce correct number of folds. However, defining growth rates as uniform and the BM as a simple viscous resistance is not sufficient to induce folds in the correct positions and shapes. Therefore, we construct detailed maps of tissue growth at fine spatial and temporal resolutions to improve the implemented growth rates.

### Wing Imaginal Disc Planar Growth Patterns Show Spatial and Temporal Heterogeneity

**Figure 4:**
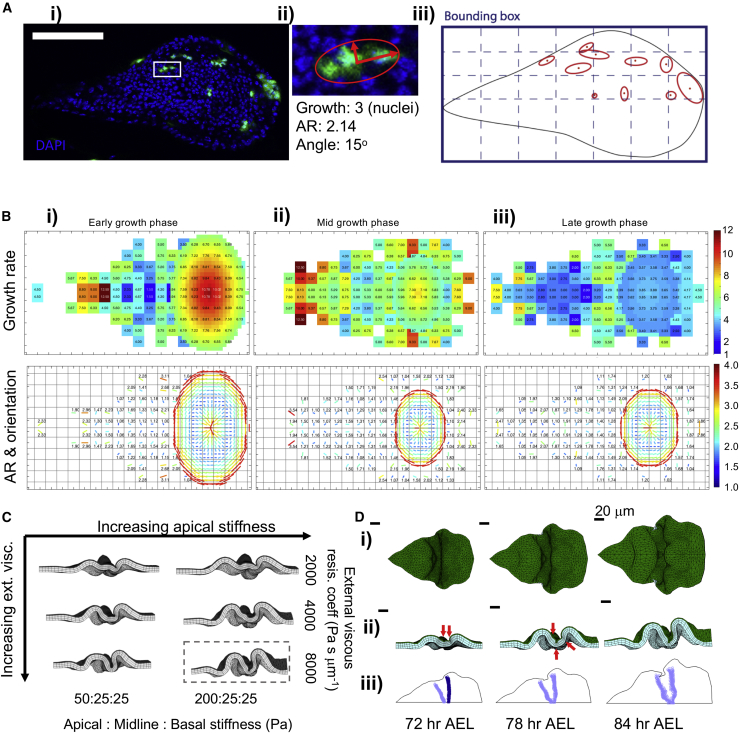
Wing Imaginal Disc Planar Growth Patterns Show Spatial and Temporal Heterogeneity (A) (i) 72 h AEL wing disc with sparse single cell clones (green), scale bar 50 μm. (ii) Clone marked by the white box in (i) with fitted ellipse (red); AR, aspect ratio. (iii) Schematic showing fitted ellipses of all clones in (i), mapped on to a grid on the bounding box of the projected image. (B) Top panels, growth maps. Bottom panels, growth orientation maps. The major axis of the average fitted ellipse are shown with lines. The length and color of the line represent the AR, orientation of the line represents growth orientation angle. (i) 48–80 h AEL, (ii) 56–88 h AEL, (iii) 72–96 h AEL. (C) Simulations with growth maps in (B). Increasing apical stiffness on rows, increasing external viscous resistance coefficient in columns. (D) Snapshots for the boxed simulation (C). (i) top, (ii) cross-section. Emerging indentations marked by red arrows. (iii) Apical indentation maps. See Video S2; Figure S3.

We experimentally measure the local growth rates via clonal analysis. By inducing sufficiently sparse single cell clones at different ages AEL, we quantify the local growth rates and generate spatial growth maps (Figure 4A). The extent of growth is defined by the number of nuclei in each clone; the orientation and aspect ratio of growth are defined by the ellipse fitted to the clone shape (Figure 4Aii). This analysis results in coupled growth rate heatmaps and orientation maps, in three-time windows as identified from the morphological quantifications (Figures 4B, S3A, and S3E; STAR Methods, Growth Rate Analysis).

The maps reveal that the wing disc harbor high spatial and temporal variability in its planar growth patterns. Specifically, notum and pouch regions have significantly higher growth than the hinge region at early stages (Figures 4B and S3E). Similar to the previous observations for the pouch region (Mao et al., 2013), the overall growth rates of the tissue are reduced as the tissue ages.

Next, we implement these measured growth rates in our simulations and model the 48 hours of development (48–96 h AEL). Here, the measured planar growth rates of three morphological age groups are applied sequentially in three equal (16 h) time windows (Figure S3Aiii), and growth is assumed to be constant through each window. With apical and basal confinement from viscous resistances, the experimental growth patterns result in folding patterns distinct from those of the uniform growth (Figures 4C and 4D; Video S2). Some indications of apical indentations emerge around the hinge region (Figure 4Dii, red arrows), and two lateral apical indentations that merge at tissue midline in later stages form (Figure 4Diii). None of the tested cases can initiate three distinct folds. This suggests that additional to the spatial-temporal variability in growth patterns, the BM should also be modeled in finer detail.

Video S2. Emergent Fold Morphology with Planar Differential Growth Rates and a Simplistic Definition of BM, Related to Figure 4 Tissue growing from 48 to 84-h AEL, with experimental planar growth rates (Figure 4B). Apical stiffness is 200 Pa (green), and stiffness for the rest of the cell body is 25 Pa (blue). Apical ECM/BM effects modeled as viscous resistances and external viscous resistance coefficient applied to both surfaces at 8,000 Pa s μm^−1^. Scale bar is 20 μm. Simulation time depicted on the frames.

### Characterization and Explicit Definition of the Basement Membrane of Wing Discs

**Figure 5:**
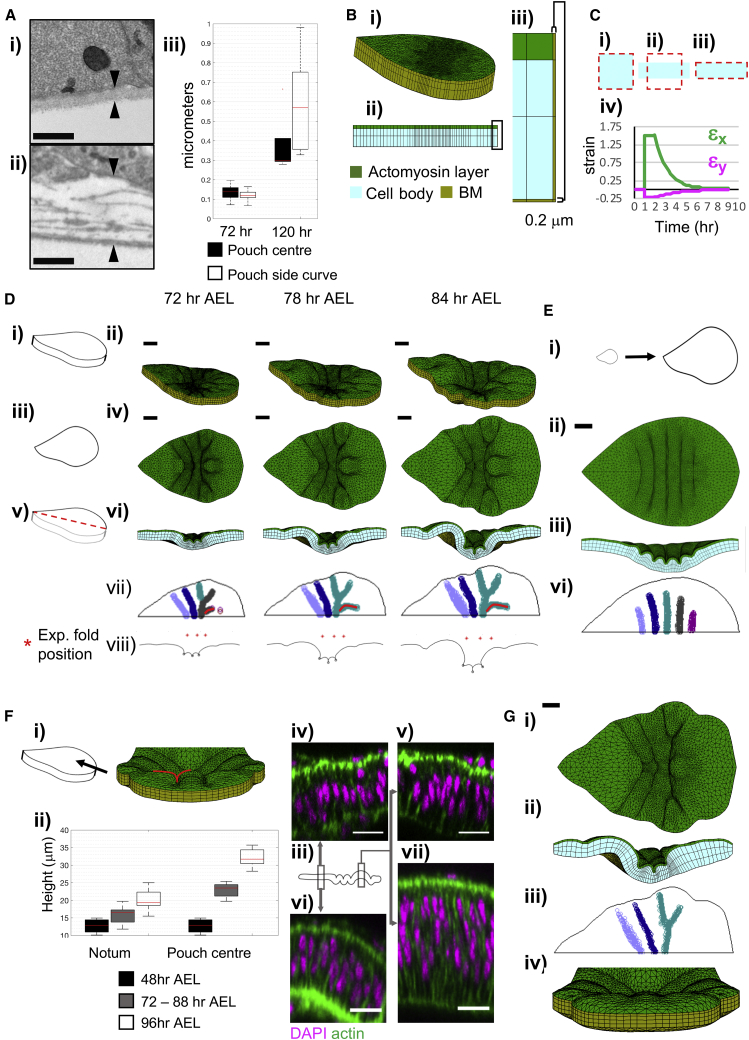
An Explicitly Defined BM and Planar Differential Growth Rates Enable Emergence of *In Vivo* Mimetic Fold Morphology (A) EM images of the wing disc BM below the pouch, (i) 72 h AEL, and (ii) 120 h AEL. Arrowheads mark the thickness measurement. Scale bars are 0.5 μm. See Figure S5C. (iii) BM thickness below the pouch. Box represents 25^th^ and 75^th^ percentiles, median in red, whiskers extend the most extreme data points. (B) (i and ii) Initial simulation mesh with the BM, (iii) close up of the boxed area in (ii). (C) Remodeling exemplified with single 2D element, (i–iii) blue quadrilateral is the current shape; dashed red lines depict the preferred shape with remodeling imposed on the reference shape. (i) Initial state, (ii) immediately after deformation, corners fixed on the x direction. (iii) Same element after area conserving remodeling. (iv) Green strains against time. Element deformed at t = 1 h, remodeling activated at t = 2 h, with half-life of 1 h. (D) Snapshots from simulation with explicit BM definition. Apical stiffness is 100 Pa, cell body stiffness is 25 Pa, BM stiffness is 1,600 Pa with renewal half-life of 8 h, apical viscous resistance coefficient is 16,000 Pa s μm^−1^, and basal is 10 Pa s μm^−1^ (Video S3). (i–vi) Schematics representing the views and the simulation snapshots. (i and ii) orthogonal perspective, (iii and iv) top, (v and vi) cross-section from the DV axis midline. All snapshots at the same scale, bars are 20 μm. (vii) Apical indentation maps. Ectopic pouch fold is marked with red line. (viii) the apical surface contour of the DV midline cross-section with computational fold positions (black circles). Experimental fold positions for 72–88 h AEL (Figure 1Ci) are in red stars. (E) Simulation results with uniform growth on the tissue plane, simulation parameters same as (D), growth rate same as Figure 3A. (i) Schematic to scale, representing uniform growth from 48 to 96 h AEL. (ii–iv) top and cross-section views and the apical indentation map at 84 h AEL. Scale bar, 20 μm. (F) Close up view of tissue pouch from ventral tip showing the ectopic folding (red line). (i) Schematic representing the view and simulation snapshot at 84 h AEL. (ii) Experimental tissue heights. Box represents 25^th^ and 75^th^ percentiles, median in red, whiskers extend the most extreme data points, n = 4 for 48 h AEL, n = 14 for 72–88 h AEL, and n = 17 for 96 h AEL. Measured at 48 h AEL, 72–88 h AEL, and 96 h AEL, the notum thickness is 12.5 ± 2.17, 15.81 ± 2.46, and 20.19 ± 2.95 μm, meanwhile the pouch center thickness is 12.5 ± 2.17, 22.96 ± 1.86, and 32.2 ± 2.26 μm, (mean and one standard deviation). (iii) Schematic representing the positions images. (iv and v) the nuclei positions of the notum (iv) and pouch (v) prior to fold formation. (vi and vii) Notum (vi) and pouch (vii) at 96 h AEL. Scale bars, 10 μm. (G) Simulation with tissue height increase at 84 h AEL, all remaining parameters are same as (D). Bar 20 μm. (i) top view, (ii) cross-section view, (iii) apical indentation map, (vi) view from ventral tip, as a comparison to (Fi). See Video S4 and Figures S4 and S5.

To characterize the morphology of the BM structure, we acquire electron microscopy (EM) images of wing discs at pre-folding stages (72 h AEL) and at the end of third instar (120 h AEL) (Figures 5Ai and 5Aii) and quantify the thickness of the BM (Figure 5Aiii). The images reveal the BM is an approximately 0.1-μm thick uniform sheet in young discs. For older discs, the BM is a more complex structure with multiple layers and thickness in the range 0.4–0.6μm. Assuming a constant thickening rate of BM at the pouch center, the average thickness between 72 and 96 h AEL is then 0.2 μm. We thus define the BM in our model as a 0.2-μm thick elastic layer encapsulating the tissue (Figure 5B).

In the simulations, the BM grows with remodeling upon deformation. Each BM element grows in the orientation of its current deformation, at a rate set by the local remodeling half-life (Methods S1). This leads to the gradual relaxation of BM deformations, and an emergent, non-homogenous growth of the BM influenced by the growth of the cellular layer. For clarity, the remodeling is represented in a 2D schematic in Figure 5C. We then simulate the development of the wing disc with a series of BM stiffness, remodeling half-life, and apical viscous resistance coefficient parameters.

### Differential Planar Growth Rates of the Tissue Constrained by an Elastic Basement Membrane Drives Precise Fold Initiation

Upon definition of the elastic BM, we initially investigate apical-basal tissue stiffness heterogeneity ranges (Figures S4A–S4C), using the experimentally measured growth rates (Figure 4B). As we increase the apical viscous resistance, the tissue starts forming buckles (Figures S4A–S4C). For the cases with increased apical stiffness and increased stiffness on both surfaces, three apical indentations emerge (Figures S4A–S4Ci). Of the two scenarios, increased apical stiffness initiates a more dome-like pouch, proportional hinge fold indentations, and lower percentage deviations from the experimental fold positions (Figures S4A–S4Cii), better mimicking the *in vivo* fold pattern.

Simulation snapshots for a setup with 100 Pa apical and 25-Pa cell body stiffness demonstrate the emerging indentations and their depth as the development progresses (Figures 5Di–5Dvi; Video S3). In the apical indentation maps, we can see the emergence of all three folds, the curved pouch border marked by the HP fold (gray, then cyan) and the emergence of the LF (cyan) (Figure 5Dvii). The folds are concentrated to the central region of the tissue, close to the experimental fold positions (Figure 5Dviii), with a total deviation of 4%. We predict the initiation of the folds requires the BM to be an order of magnitude stiffer than the cellular layer, which indeed has been inferred to be the case (Keller et al., 2018). As long as the BM is dramatically stiffer than the cellular layer, this phenotype can be produced with a large range of stiffness and remodeling half-life parameter sets for the BM, relative changes in one compensating for the other (Figure S5A). Simulating the same parameter set as Figure 5D with uniform growth reveals numerous symmetric ripples on the apical surface (Figure 5E). This signifies the importance of planar differential growth in the precise selection of the number and position of the folds.

Video S3. Correct Fold Morphology Emerges with Planar Differential Growth Rates and an Explicit Definition of the Elastic BM, Related to Figure 5D Tissue growing from 48 to 84-h AEL, with experimental planar growth rates (Figure 4B) and an explicitly defined BM (yellow). Apical stiffness is 100 Pa (green), and stiffness for the rest of the cell body is 25 Pa (blue). Apical ECM effect modeled as a viscous resistance with external viscous resistance coefficient of 16,000 Pa s μm^−1^. BM stiffness is 1,600 Pa, and BM renewal half-life is 8 h. Scale bar is 20 μm. Simulation time depicted on the frames.

Our simulation also initiates an ectopic buckle on the pouch, which is not observed in live tissue (Figure 5Dvii, red line, Fi). To decipher what may be driving the resistance of the pouch region to such buckling, we turn our attention to tissue thickness. Our analysis reveals, through the 48 h of our interest, the wing disc increases its thickness in a non-uniform manner (Figure 5Fii), with the pouch region becoming relatively thicker than the rest of the tissue. The emergent tissue thickness increase during the simulations brings the pouch height to 17.3μm, and notum height to 13.3μm at 84 h AEL, from the initial uniform height of 12.5μm at 48 h AEL. This is well below the experimental observations (Figure 5Fii); therefore, the thickness increase should be an active growth input.

Implementing this growth in the tissue thickness prevents pouch surface buckling (Figures 5G and S4D, and Video S4), with a total fold position deviation of 10% (Figure S5A). Our simulations predict that within the mechanical context where the hinge folds are initiated, the relative thickness of the pouch region protects it from further buckling that would be otherwise induced by the compression.

Video S4. Differential Thickness Increase Confines the Folds to the Hinge Region, Related to Figure 5G Tissue growing from 48 to 84-h AEL, with experimental planar growth rates (Figure 4B), differential tissue thickness increase (Figure 5Fii, see STAR Methods), and an explicitly defined BM (yellow). Apical stiffness is 100 Pa (green), and stiffness for the rest of the cell body is 25 Pa (blue). Apical ECM effect modeled as a viscous resistance with external viscous resistance coefficient of 16,000 Pa s μm^−1^. BM stiffness is 1,600 Pa, and BM renewal half-life is 8 h. Scale bar is 20 μm. Simulation time depicted on the frames.

### Emergent Three-Fold Pattern Is Robust against Volume Variability

Throughout the analysis, number of nuclei is used as a surrogate for tissue growth. To identify the potential impact of cell volume variations, we generate volume variability maps from the clone analysis and obtain volume scaled growth maps (Figures S5Fi–S5Fiii). Simulations with the volume scaled growth maps can capture the main pattern of the three folds, but additional ectopic buckling along the anterior-posterior axis between HH and HP folds is seen (Figure S5Fiv). The core pattern of fold initiation with differential growth is robust against observed variations in cell volume, but approximating the tissue growth as a combination of the number of nuclei and observed volume does not improve model predictions. The observed volume of a clone is a combination of the cell’s preferred volume and its deformations, as opposed to the nuclei count and the definition of growth in the model. This disparity, combined with the challenges of accurate volume measurements in the complex pseudostratified wing disc epithelia, negates potential additional information a true volume measurement could provide. As such, in line with our parsimonious approach throughout the paper, we continue to approximate growth with number of nuclei and assume constant cell volume throughout development in further model tests.

### Early Growth Pattern Is Sufficient for Correct Fold Initiation

Our results so far demonstrate the importance of planar differential growth in defining fold positions. The simulations reveal the fold initiation starts by the end of the early growth phase (Figure S4E). It has been shown that wing discs dissected immediately prior to fold initiation are still able to initiate folds *ex vivo* upon blocking cell division (Sui et al., 2018). With these, we investigate if the early growth rates are sufficient to initiate the folds upon significant perturbation to growth thereafter. We run simulations where the early growth rates in Figure 4Bi are applied for the first 16 h (48 to 64 h AEL) as in the control case, and then the growth is continued in the simplest form, with the uniform rates. The early growth rates followed by uniform growth are sufficient for the emergence of *in vivo* mimicking fold morphology (Figure S6Ai). Similarly, WT growth up to 64 h AEL, followed by non-oriented growth at the experimental growth magnitudes, does not alter the emerging fold morphology (Figure S6Aii). Additionally reducing the growth rate to 50% of the experimentally measured rates results in a minimal discontinuity in the fold pattern, but the structure is preserved (Figure S6Aiii). When the simulations are run with control growth rates, but the accumulated forces are relaxed prior to the emergence of the folds, the emerging morphology is perturbed. The final topology has loose fold formation, and the pouch curve shrinks compared to the control simulations (Figure S6B). These results suggest that the early growth rates measured prior to initiation of the folds, and the corresponding force accumulation, are critical for correct tissue morphology.

### The Simulations Successfully Predict the Disrupted Fold Morphology upon Perturbations of Planar Differential Growth Patterns in Overgrowth Clones and *Wingless* Mutants

**Figure 6:**
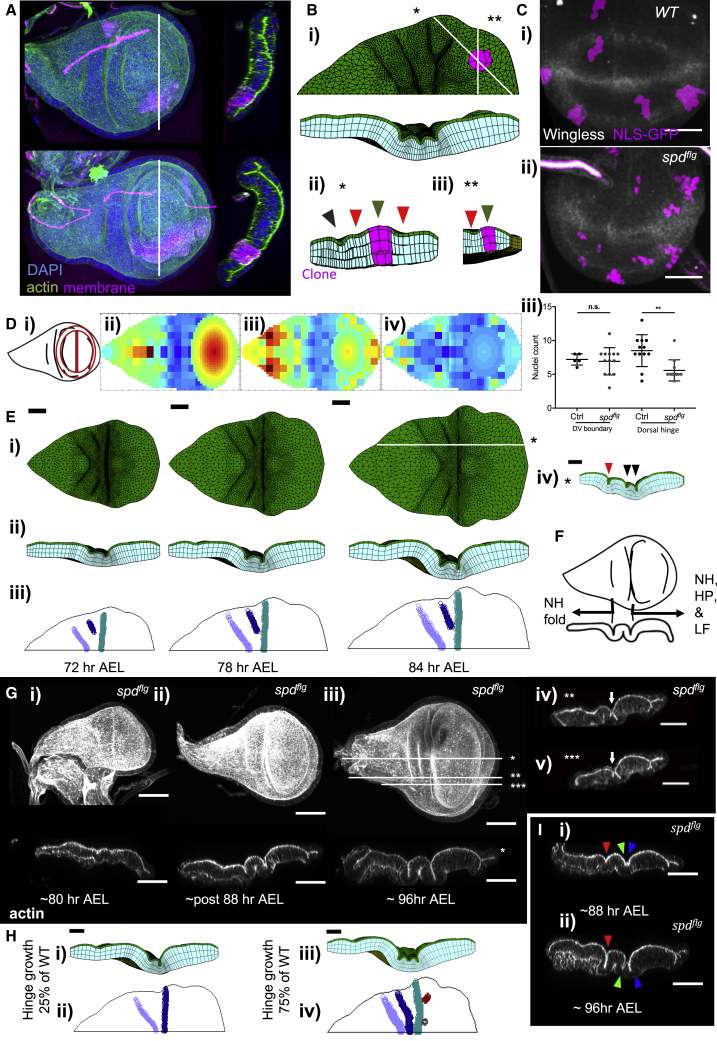
Simulations Successfully Predict the Disrupted Fold Morphology of Overgrowth Clones and *Wingless* Mutants upon Perturbations of Planar Differential Growth Patterns (A) Wing discs with insulin overgrowth clones (magenta, membrane labeled by CD8-mCherry) - 96 h AEL. Maximum projection of the top view, cross-sections through the whites lines. (B) Simulation snapshots for overgrowth clone at 75 h AEL, initial clone diameter is 4 μm (in line with wing disc cell diameters), growth is 3 times the WT without orientation, (see also Figure S6C). All physical properties same as Figure 5D. (i) Top panel: top view with the clone highlighted, bottom panel: cross-section from the DV axis midline. (ii and iii) the cross-section from the white lines in (i), (ii) is (^∗^), and (iii) is (^∗∗^). Green arrowheads point to apical bulging, red point to ectopic folds at the borders of the clone, gray points to the LF. (C) *spd*^*flg*^ mutant growth quantification with clone analysis, clones in magenta. Scale bar, 25 μm. (i) WT, (ii) *spd*^*flg*^ mutant. (iii) Quantification of clone size in the proximity of the wg expression band at the pouch DV boundary (n = 5 for WT, n = 13 for *spd*^*flg*^), and the wingless expression ring, the dorsal side of pouch only (n = 12 for WT, n = 12 for *spd*^*flg*^). Means and one standard deviations marked, ** depicts significant difference with p-value < 0.01 in a two tailed t test, n.s. marks not significant with p-value > 0.05. See also Figure S6D. (D) (i) Schematic marking the pattern of wingless expression in WT. (ii–iv) the reduction of growth in the *spd*^*flg*^ defined in between the experimental positions of HN fold and center of the pouch, excluding the pouch itself. Growth maps for 50% growth reduction as sampled from the extended maps (see Figure S4) through the simulation. (i) 48–64 h AEL, (ii) 64–80 h AEL, and (iii) 80–96 h AEL. All represented as corresponding growth increase in 24 h. Color coding scale same as Figure 4B. (E) Simulation with 50% reduced hinge growth. (i and ii) Simulation snapshots from (i) top and (ii) cross-section. (iii) Apical indentation maps. All simulation parameters except for the growth rates are same as Figure 5D. (iv) Lateral cross-section from the white line marked on (i) 84 h AEL, red arrowhead marks the NH fold, black arrowheads mark the laterally initiating folds, reminiscent of HH, HP folds, or the LF. Scale bars, 20 μm. (F) Schematic displaying the collapse of HH and HP folds and the LF. (G) the morphology of the mutant wing discs (i–iii) during the emergence of the folds. Scale bars, 50 μm. (iv and v) Cross-sections from the lateral side, demonstrating the emergence (white arrow) of lateral folds that collapse with the pouch side fold before midline. Of the white lines marked on (iii), (iv) is (^∗∗^) and (v) is (^∗∗∗^). (H) Simulation results for reduced hinge growth at (i and ii) 25% and (iii and iv) 75% of WT. All remaining simulation parameters same as Figure 5D. Panels, color coding, and scale bars are same as (E). (I) *spd*^*flg*^ mutant phenotypes with emergence of a residual small peak within the grove of the ventral side fold of the mutant, highlighting onefold is lost as a result of HH, and HP folds merging. NH, HH, and HP folds are marked in red, green, and blue arrowheads, respectively. See also Figure S6.

Next, we challenge our simulations with perturbations in tissue growth, both overgrowth and undergrowth. Given the sufficiency of early growth rates for patterning the tissue, we must select perturbations that are effective prior to the formation of any folds. First, we experimentally induce overgrowth clones via increasing insulin signaling at 48 h AEL, by expressing a constitutively active form of the insulin receptor. The clones induce apical and basal bulging of the tissue, with ectopic fold initiation at the clone boundaries (Figure 6A). We simulate overgrowth clones with a range of growth rates, and we can capture the same deformations (Figure 6B). The apical bulging is visible from 200% growth increase, ectopic folding can be observed at 300% and above (Figure S6C), which is in line with overgrowth magnitudes observed for other perturbations in the wing disc (Genevet et al., 2009).

Next, we test undergrowth. Again, this mutant must have an effect before the folds form. The expression of *wingless* (*wg*) localizes in two concentric rings encapsulating the pouch region from early third instar, before the folds start initiating. *spade*^*flag*^ (*spd*^*fg*^) mutations in *wg* lead to loss of *wg* expression at the inner ring, coinciding with the hinge. Consequently, the mutant has reduced growth in the hinge, and reduced size in the corresponding region of the adult wing (Neumann and Cohen, 1996, Rodríguez Dd et al., 2002). First, we carry out a clone growth rate analysis to demonstrate growth is reduced in *spd*^*fg*^ mutant during 2^nd^ instar stage (Figure 6C). We quantify the clone size in the region corresponding to the dorsal side of the inner ring that is lost in *spd*^*fg*^. This quantification reveals a 35% reduction in clone size for the mutant with respect to the wild type (Figure 6Ciii). *Wg* expression in the pouch DV boundary is preserved in the mutant. As an internal control, we quantify the clone size in this *wg* expression band and show that there is no reduction in growth at the DV boundary (Figure 6Ciii). These clearly demonstrate that the *spd*^*fg*^ mutant reduces growth specifically in the wing disc hinge during early wing disc development (48–72 h AEL), prior to fold initiation.

Next, to predict the wing disc phenotype of this mutation, we run simulations where hinge growth is reduced in the range 75% to 25% of WT. At 50% growth reduction (Figure 6D), the simulated mutant loses the three-fold morphology and displays only two folds at the midline of the disc (Figure 6E). On the lateral side, a third fold starts emerging and collapses on the pouch side fold before reaching the midline (Figure 6Eiv). It follows that the *spd*^*fg*^ mutant should be able to form the NH fold, while HH and PH folds and the LF will collapse into a single fold at the tissue midline (Video S5) (Figure 6F). To test our prediction, we examine *spd*^*fg*^ mutant wing discs (Figure 6G). The morphology of mutant wing discs at sequential developmental stages clearly demonstrates the emergence of a two-folded morphology (Figures 6Gi–iii) instead of three. Further, investigating the lateral cross-sections of the tissue at 96 h AEL shows that a third fold initiates at the lateral regions, yet collapses with the pouch side fold before reaching the tissue midline (Figures 6Giv and 6Gv), matching with the model predictions (Figure 6Div; Video S5). Simulations also reveal a dose dependent perturbation of the fold structure. At growth rates as low as 25% of WT, the third fold does not emerge from the lateral sides (Figures 6Hi and 6Hii), whereas at 75%, three folds form at the midline, albeit being more compact than the WT (Figures 6Hiii and 6Hiv). Then going back to the experiments, we could identify cases where small peaks emerged at the groove of the pouch side fold at tissue midline (Figure 6I). This further supports the hypothesis that the HH fold collapses with the HP fold and the LF in the *spd*^*fg*^ mutant. Depending on the level of growth perturbation, the loss of the HH fold can be observed gradually, again matching with the predictions of the simulations.

Video S5. Predictions of the Emergent Morphology for *spd*^*fg*^ Mutation of the *wingless*, Related to Figure 6 Tissue growing from 48 to 84-h AEL, with planar differential growth rates mimicking the *spd*^*fg*^ mutation of the *wingless*. The growth of the hinge is reduced by 50% for this particular simulation (Figure 6A). There is differential tissue thickness increase (Figure 5Fii, see STAR Methods). Explicitly defined BM (yellow) has stiffness of 1,600 Pa, and renewal half-life of 8 h. Apical stiffness is 100 Pa (green), and stiffness for the rest of the cell body is 25 Pa (blue). Apical ECM effect modeled as a viscous resistance with external viscous resistance coefficient of 16,000 Pa s μm^−1^. Scale bar is 20 μm. Simulation time depicted on the frames.

## Discussion

Here, we present a computational model of tissue growth and morphogenesis, incorporating spatial and temporal heterogeneity in growth rates and orientations, BM mechanics and remodeling, and physical property heterogeneities within different layers of the tissue. Coupled with the stiff apical surface and BM mechanics, we demonstrate that the planar differential growth rates are key in defining the positions of epithelial folds of the wing disc. Upon identifying early growth phases as sufficient for the initiation of folds, we make predictions on the emergent morphology upon perturbation of early growth rates. By changing only the planar differential growth rates *in silico*, we successfully predict overgrowth clone and *wingless* mutant morphologies *in vivo*. With our computational analysis, we propose a mechanism whereby planar differential growth rates define epithelial fold initiation positions.

In the BM mechanics, we identify, a large range of parameters will suffice as long as the BM is elastic and sufficiently stiff (8 times the apical stiffness and above). This observation could explain the robust folding of the wing disc against the high spatial and temporal variability in BM structure (Figure 5A). Our simulations also show that some form of viscous resistance on the apical surface is necessary. The apical ECM has a different composition (Ray et al., 2015) and likely independent dynamics from the BM (Keller et al., 2018). The composition of the lumen, and any possible tethering interactions with the peripodial layer (Gibson and Schubiger, 2000), would resist apical surface movement with fluid-like viscous mechanics. Currently, we do not have any means of acquiring this viscous resistance of the apical ECM, yet our modeling approach signifies its importance.

While our model is able to successfully generate the three-fold pattern, the timing of fold initiation in our simulations deviate somewhat from the experimental observations (65 h AEL in simulations compared to 76–80 h AEL (Sui et al., 2018) *in vivo*, Figure S4E). Temporal dynamics of tissue and BM physical properties could lead to the offset in our fold initiation timing. With its complex structural variability (Figure 5A), it is not possible to assume monotonically increasing or decreasing BM stiffness, without extensive further studies. The stiffness of the tissue itself is most likely altered through the same period, given that the residual tension of the tissue has been shown to reduce over time (Rauskolb et al., 2014). The fact that we can observe fold initiation in the simulations prior to the emergence of the folds in the experiments indicates the accumulation of cell mass due to planar differential growth is sufficient for fold initiation, yet the tissue may not have reached the enabling physical state before 76 h AEL.

At later stages of the simulations, the successfully initiated hinge folds do not progress into fully established folds (Figure S5B). Our simulations suggest that once the folds are initiated with planar differential growth rates, additional mechanisms should be activated to progress these indentations into folds. Indeed, cell shortening, alteration of the microtubule and actin networks, modification of both interaction with the BM through integrins and the BM structure itself through MMPs, have all been reported as necessary requirements for progression of wing disc folding, each fold requiring particular subsets of modifications (Shen et al., 2008, Sui et al., 2012, Sui et al., 2018). These mechanisms and the mechanics of the accumulated cell mass due to differential growth can act together in a sequence of events that are temporally difficult to separate, acting concurrently or consecutively. Our findings set the scene for further theoretical and experimental investigation on the feedback mechanisms between the morphology of the apical surface and the signaling pathways regulating local growth, cell shape, and BM interactions. In our model, we impose the growth patterns from experimental observation. Further analysis is needed in order to determine the coupling between this growth distribution and other genetic or chemical factors.

In conclusion, our results suggest planar differential growth as a mechanism for determining tissue fold positions, independent of active force generation. With wider implications, we suggest that the growth patterns giving tissues their final size can also regulate their architecture. We show that forces may not always result in instantaneous morphology changes, but can result in delayed morphogenesis. Stresses may accumulate early during development, even without any obvious changes in tissue morphology, but these may be critical for the precise sculpting of the tissue later in development.

## STAR★Methods

### Key Resources Table

**Table:** 

REAGENT or RESOURCE	SOURCE	IDENTIFIER
**Antibodies**		

Wingless MIgG1	DSHB	4D4; RRID: AB_528512
Donkey anti-mouse RRX	Jackson Immuno Research	715-295-151; RRID: AB_2340832

**Chemicals, Peptides, and Recombinant Proteins**		

DAPI	Sigma-Aldrich	D8417; RRID: AB_2307445
Phalloidin Alexa-647	Life Technologies	A22287
Phalloidin Alexa-555	Life Technologies	A34055
Hoechst	Sigma-Aldrich	B2261
Fluoromount G Slide mounting medium	Southern Biotech	0100-01
Bovine Serum Albumin	Sigma	A7030
Triton X-100	Sigma	T8787
16% w/v formaldehyde (Prediluted with PBS to 4% for fixation protocols)	TAAB Laboratories	F017/3
EM grade formaldehyde 36%	TAAB Laboratories Equipment Ltd	F003
EM grade glutaraldehyde 25%	TAAB Laboratories Equipment Ltd	G011
Osmium Tetroxide 2%	TAAB Laboratories Equipment Ltd	O005
Potassium ferricyanide	TAAB Laboratories Equipment Ltd	P018
Dodecenyl Succinic Anhydride	TAAB Laboratories Equipment Ltd	D027
2,4,6- Tri(Dimethylaminomethyl)	TAAB Laboratories Equipment Ltd	D032
Methyl Nadic Anhydride	TAAB Laboratories Equipment Ltd	M011
TAAB 812 resin	TAAB Laboratories Equipment Ltd	T023
Tannic Acid	TAAB Laboratories Equipment Ltd	T046
Lead Nitrate	TAAB Laboratories Equipment Ltd	L005
Potassium Ferricyanide	Sigma-Aldrich	P8131
Sodium citrate	Sigma-Aldrich	S4641

**Experimental Models: Organisms/Strains**		

*w;; actin-FRT-CD2-FRT-Gal4, UAS-GFP*^*NLS*^	Neufeld et al., 1998	N/A
hsFLP	Bloomington *Drosophila* Stock Centre	BDSC8862
*Yw*	Bloomington *Drosophila* Stock Centre	
*wingless*^spd-fg^	Bloomington *Drosophila* Stock Centre	BDSC1005
*yw*hsFLP; *wg*^*spd-fg*^; S-T	Y.Mao	N/A
hsFLP; UAS-InR^A1325D^/CyO; MKRS/TM6B	N. Tapon	N/A
*w; actin-FRT-y-FRT-Gal4-UAS-CD8-mCherry/CyO; arm-GFP/TM6*	Y.Mao	N/A

**Software and Algorithms**		

Microsoft Excel 16	Microsoft	N/A
GraphPad Prism 7	GraphPad Software	N/A
Matlab 2016	Mathworks	N/A
ImageJ 1.51w	(Schneider et al., 2012) https://imagej.nih.gov/ij/docs/install/osx.html	N/A
Triangle - A two-dimensional quality mesh generator and Delaunay triangulator	(Shewchuk, 2005) https://www.cs.cmu.edu/∼quake/triangle.html	N/A
Tissue morphogenesis FE simulation software	Bespoke model code developed in C++ for this study.	N/A

### Lead Contact and Materials Availability

Further information and requests for resources and reagents should be directed to and will be fulfilled by the Lead Contact, Yanlan Mao (y.mao@ucl.ac.uk). All unique and/or stable reagents and the custom code generated for the manuscript are available from the Lead Contact upon request.

### Experimental Model and Subject Details

#### *Drosophila* melanogaster

Fly stocks were raised in non-crowded conditions on standard cornmeal molasses fly food medium at 25°C, unless otherwise indicated. Briefly, the fly food consisted of, per 1L, 10g agar, 15g sucrose, 33g glucose, 35g years, 15g maize meal, 10g wheat germ, 30g treacle, 7.22g soya flour, 1g nipagin, 5ml propionic acid. Male and female larvae were dissected at a range of developmental stages from 48hr AEL to 120hr AEL for experiments.

Strains used are listed in Key Resources Table, and include: For clone generation, tissue shape, and morphology measurements: hsFLP;; (BDSC8862) and w;; Act<CD2<GAL4, UAS-GFP (Neufeld et al., 1998). For wildtype height measurements: yellow white (yw;;) (BDSC). For *wingless* (*wg*) mutant analysis: ; *wg* ^spd-fg^; (BDSC1005), *wg* clones *yw*hsFLP; *wg*^*spd-fg*^; S-T. For overgrowth clones with perturbation of the insulin pathway: hsFLP; UAS-InR^A1325D^/CyO; MKRS/TM6B and w; Act<y<Gal4, UAS-CD8-mCherry/CyO; arm-GFP/TM6.

### Method Details

#### Clone Generation

To generate heat shock flip-out GFP clones of the correct density for growth rate analysis, the following regimes were used: for growth rates at 48–72h, 56-80h and 64–88h, heat shock was performed at 48h, 54hr or 64hr AEL respectively, for 12-20 min and dissected 24h later. For growth rates at 72–96 h, heat shock was performed at 72h for 10 min and wing discs dissected 24h later. All heat shocks were carried out at 37°C. To investigate spontaneous expression of GFP, we carried out non-heat shock controls (n=22 wing discs). Spontaneous GFP expression was indeed observed in larval epidermis, within the trachea and myoepithelial cells in the proximity of the wing disc. No such spontaneous expression is observed upon inspection of the wing disc columnar epithelium. For induction of insulin overgrowth clones marked by CD8-mCherry, heat shock was performed at 48hr AEL for 10 min at 37°C, followed by a 48hr growth before dissection. For the induction of flip-out GFP clones for growth rate analysis of the *wingless* mutant, the heat shock was performed at 48hr AEL for 10 min at 37°C and grown for 24 hrs before dissection.

#### Immunostaining and Imaging

Larval wing imaginal discs were dissected and stained as per the procedure described in (Gaul et al., 1992). In brief, wing discs were dissected at the appropriate age in ice cold PBS for up to 15 min and fixed in 4% formaldehyde in PBS, at room temperature, for 30 min.

For *wingless* mutants, fixed discs were repeatedly washed within a 40 min period in 0.3% PBT, followed by repeated washes with 0.5% BSA, 0.3% PBT for a further 40 min. Primary antibody, mouse anti-Wingless was prepared in 0.5% BSA, 0.3% PBT at 1:100 concentration and incubated overnight at 4^o^C. Washes were repeated as prior to primary antibody incubation. Secondary antibody, goat anti-mouse RRX (Jackson ImmunoResearch) (1:500), Alexa fluor 647-Phalloidin (Cell Signalling and Life Technologies) (1:20) and Hoechst (Sigma-Aldrich) (1:500) were prepared in 0.5% BSA, 0.3% PBT and incubated for 1 h at room temperature. Wing discs were washed repeatedly for 1 h in 0.3% PBT, prior to rinsing in PBS.

For *yw* larva used for height measurements and flip-out clone larva used for growth rate analysis, dissected and fixed wing discs were washed in 0.3% PBT repetitively for 20 min, then immediately incubated with Alexa fluor 647-Phalloidin (Cell Signalling and Life Technologies) (1:20) and Hoechst (Sigma-Aldrich) (1:500) in 0.3% PBT for 15 min at room temperature. Wing discs were washed repetitively for 30-40 min, and then rinsed with PBS.

Fixed and stained wing discs were mounted in fluoromount G Slide mounting medium (Southern Biotech) for imaging.

Wing discs were imaged on a Leica SP5 and SP8 inverted confocal microscope with a 40X oil objective at 1-2X zoom, 0.341 μm depth resolution and 512 by 512 or 1024 by 1024 pixel resolution.

#### Electron Microscopy

Wing discs were fixed in 2% formaldehyde/ 1.5% glutaraldehyde in PBS for 30 min prior to being flat, sandwich-embedded in 2.8% low melting point agarose dissolved in PBS. Once set, asymmetric cubes of agarose were cut out containing the wing discs, and they were secondarily fixed for 1 h in 1% osmium tetroxide/1.5% potassium ferricyanide at 4°C. Further fixation and contrast enhancement was achieved with, 1% tannic acid for 45 min. Samples were then dehydrated in increasing concentrations of ethanol solutions and embedded in Epon resin (TAAB 812). The 70nm ultrathin resin sections were cut with a diamond knife (Diatome) using an ultramicrotome (UC7; Leica) and sections were collected on formvar-coated slot grids and stained with lead citrate. Discs were imaged using a 120kV transmission electron microscope (Tecnai T12; FEI) equipped with a ccd camera (Morada; Olympus SIS).

#### The Model Definition

A detailed definition of the computational model, simulation results analysis methods and a table providing the parameter definitions together with the values used in the simulations are provided in the Methods S1, Methodology of the Computational Model.

### Quantification and Statistical Analysis

#### Tissue Dimension Measurements

The tissue size is measured from maximum projection images. DV length is defined as the longest axis from ventral tip of the pouch to the dorsal tip of the notum. AP length is measured to be the longest axis of the tissue perpendicular to the measured DV axis. The number of discs measured for each stage are: 4 discs at 48hr AEL; 32 discs for early stages with no fold initiation; 38 discs for DV and 32 discs for AP for middle stages with some fold initiation; 22 discs for DV contour length, 31 discs for DV length and 17 discs for AP length for 96 h AEL discs. Fold positions are measured at the longest axis in tissue midline, corresponding to the axis of DV length measurement. Each fold position is normalized to DV length, dorsal tip being 0 and ventral tip 1. The NH fold position is averaged from 19 wing discs, HH fold from 26 and HP fold from 16 discs.

#### Growth Rate Analysis

The clone position, aspect ratio, and orientation were calculated with automated segmentation and ellipse fitting, number of nuclei in each clone was counted manually, all using ImageJ (Schneider et al., 2012). To convert the growth information of each clone to spatio-temporal maps of tissue growth, we go through age classification of the wing disc, alignment of wing morphology to average, binning the clone positions on a 2D projection of the tissue, and averaging data points, followed by overlaying the pouch growth rates from the literature (Mao et al., 2013), to generate the growth maps of Figure 4B. Next, the data points on the growth maps are intra- or extra-polated to cover the empty spaces of the map grid, to allow for the maps to be smoothly read during the simulations (Figures S3D and S3E).

For each clone, the manually counted number of nuclei determine the growth rate, the fitted ellipse determines the growth orientation and aspect ratio (Figure 4Aii). The centre of the fitted ellipse defines the position of the measured growth as normalised to the tissue bounding box. Assuming symmetry in the anterior-posterior axis, all single clone measurements are binned on one half of the bounding box (Figure 4Aiii).

The wing discs are divided into three age groups depending on their morphology. The first indication of folds on wing discs occurs between 72-80 h AEL (Sui et al., 2018). Therefore the wing discs with no visible fold initiation, except for minor actin accumulation on the fold region, corresponding to up to 80hr AEL age (Figure S3Ai) are classified into the “early phase”. The wing discs with at least one, mostly two to three initiated folds, but not reached to fully folded morphology, corresponding to the range 80-88hr AEL age (Figures S3Aii–S3Aiv) are classified into mid-phase and finally, wing discs with all three folds formed, corresponding to 96 h AEL age are classified into “late phase”. The age definitions are not clear-cut at all times, therefore we will refer to disc growth periods as early, mid, and late phase throughout the manuscript, referring to the above morphological characterisation (Figure S3A).

Upon division of the data points into age groups, where the wing disc has any markers for initiation of the first (HH) fold, the HH fold position of the individual disc is aligned to the average HH fold position, and the position of the clones updated (Figure S3B). This alignment step is not applicable to wing-discs of the earliest growth phase, where no folds are visible. Next, all the clones for a selected time point are binned on a 20 by 10 grid, according to their normalised planar positions within the bounding box of the tissue (Figure 4Aiii). Any grid bin with less than two data points is treated as empty. The growth data from the clones in each bin are averaged, Gaussian average is applied to number of nuclei, and the orientation angles, the orientation of the long axis of the fitted ellipse, are defined to be within π/2 degrees of each other before taking a Gaussian average. The aspect ratios are averaged with geometric average.

The growth rates of the pouch region of the wing disc have previously been characterised in high detail by Mao et al. (2013). We overlay these measurements onto the generated growth and orientation maps by defining the position of the HP fold as the dorsal tip of the pouch and using the pouch sizes measured in this study (Figure S3C). Finally, with the assumed anterior-posterior symmetry, the resulting growth map is reflected to the remaining half, and our final spatial-temporal growth profiles to be utilised in our simulations are obtained (Figures 4B, S3Aiii, and S3E).

The simulation requires a complete map, without gaps, such that each element can read its growth rate at each time step. Therefore, we fill the empty points of the grid by interpolating the existing measurements. Starting from the centre of the grid and moving out radially (Figure S3Di), once an empty grid point is detected, all the populated points within its eight immediate neighbors are averaged to fill the grid point (Figure S3Dii). The order of filling is of significance, as once a point is filled with averaging the neighbors, it will be counted as a populated point in following iterations. This allows us to fill the grid points at all regions, and we obtain the maps in Figure S3E. Of note, the corner points are not necessarily sampled in the simulation, as the emergent simulation tissue shape is similar to that of the experiments, nevertheless, the map should cover a slightly larger area then the immediate experimental boundary to ensure continuity. For an example of the region sampled throughout the simulations, see Figure 6D. While reading the growth rates from these maps, each element of the simulation takes its centre point normalised to tissue bounding box, reads the closest four corner values from the growth and orientation maps, and interpolates the actual growth rate/orientation to apply depending on its distance from each of the four corners.

Growth in apical-basal axis is calculated from measurement of tissue thickness (Figures 5Fii–5Fvii and S5D). The growth rate is directly calculated form the height increase, yet one complexity here became the pseudostratification of the tissue. As the tissue grows, the nuclei become pseudostratified, first in the pouch, followed by the notum (Figures 5Fiii–5Fvii and S5D). Coinciding with the relative pouch thickness increase, the pseudostratification is visible in the pouch region as early as the initiation of the HH fold as an apical indentation (Figure 5Fv), while notum nuclei are still organized in a single layer (Figure 5Fiv). As the development progresses, the pseudostratification can be seen everywhere, the extent being significantly higher in the pouch (Figures 5Fvi and 5vii). The measurements demonstrate tissue thickness can increase without pseudostratification (Figure 5Fiv), indicating addition of material to tissue height independent of cell division, i.e., the nuclei count from which we derive our planar growth rates. On the other hand, the pouch region of the tissue increases in height faster, to a greater extent, and pseudostratification is more predominant in this region (Figures 5Fiii and 5Fvi), which should influence our definition of planar growth. To account for this difference, we allowed for tissue height increase to the level of the notum thickening, without altering the planar growth rates, reflecting the cell height increase independent of nuclei stratification. The additional height increase observed for the pouch region, the difference between the notum and pouch height increases, is reduced from the planar growth rates, “using up” the increase in nuclei numbers.

#### Clonal Volume Measurement

We quantify the volume variability of the cells through an analysis analogous to growth rate analysis. The total volume of each clone is approximated by the maximum projected clone area and the height the clone spans in the z-stack. Then combining this data with the number of nuclei in the clone, we obtain the average cell volume in each clone. Normalising the measured cell volume to the average of the sample set, and following the same spatial and temporal classification of clones as in the growth rate analysis, we generate the volume variability maps (Figure S5F, top panels). Then we scale our growth maps with these volume maps, and obtained the volume scaled growth maps (Figure S5F, bottom panels).

Prism 7 was used for statistical analysis (Figure 6C). Two-tailed t test was used with the exact n values used for each of the experiments. The following statistical significance cut off was applied:

^∗^ p < 0.05,

^∗∗^ p < 0.01,

^∗∗∗^p < 0.001,

^∗∗∗∗^p < 0.0001.

### Data and Code Availability

The authors declare that the data supporting the findings of this study and custom code generated for the manuscript are available from the corresponding author, Yanlan Mao (y.mao@ucl.ac.uk), upon reasonable request.

## Supplemental Information

Document S1. Methods S1, Figures S1–S6, and Tables S1–S3

Document S2. Article plus Supplemental Information
