# Supplementary material for: Planar Differential Growth Rates Initiate Precise Fold Positions in Complex Epithelia
Source: Dev Cell. 2019 Nov 4;51(3):299–312.e4. doi: 10.1016/j.devcel.2019.09.009 (PMC6838681; doi:10.1016/j.devcel.2019.09.009)
Supplement: Document S1. Methods S1, Figures S1–S6, and Tables S1–S3 [file mmc1.pdf]

Developmental Cell, Volume *51*

Supplemental Information

Planar Differential Growth Rates Initiate

Precise Fold Positions in Complex Epithelia

Melda Tozluoǧlu, Maria Duda, Natalie J. Kirkland, Ricardo Barrientos, Jemima J. Burden, José J. Muñoz, and Yanlan Mao

Index

Supplementary Figure Legends

Supplementary Figure 1: related to Figure 2
Supplementary Figure 2: related to Figure 3
Supplementary Figure 3: related to Figure 4
Supplementary Figure 4: related to Figure 5
Supplementary Figure 5: related to Figure 5
Supplementary Figure 6: related to Figure 6

Method S1: Methodology for the computational model, related to STAR Methods

Supplementary Figures

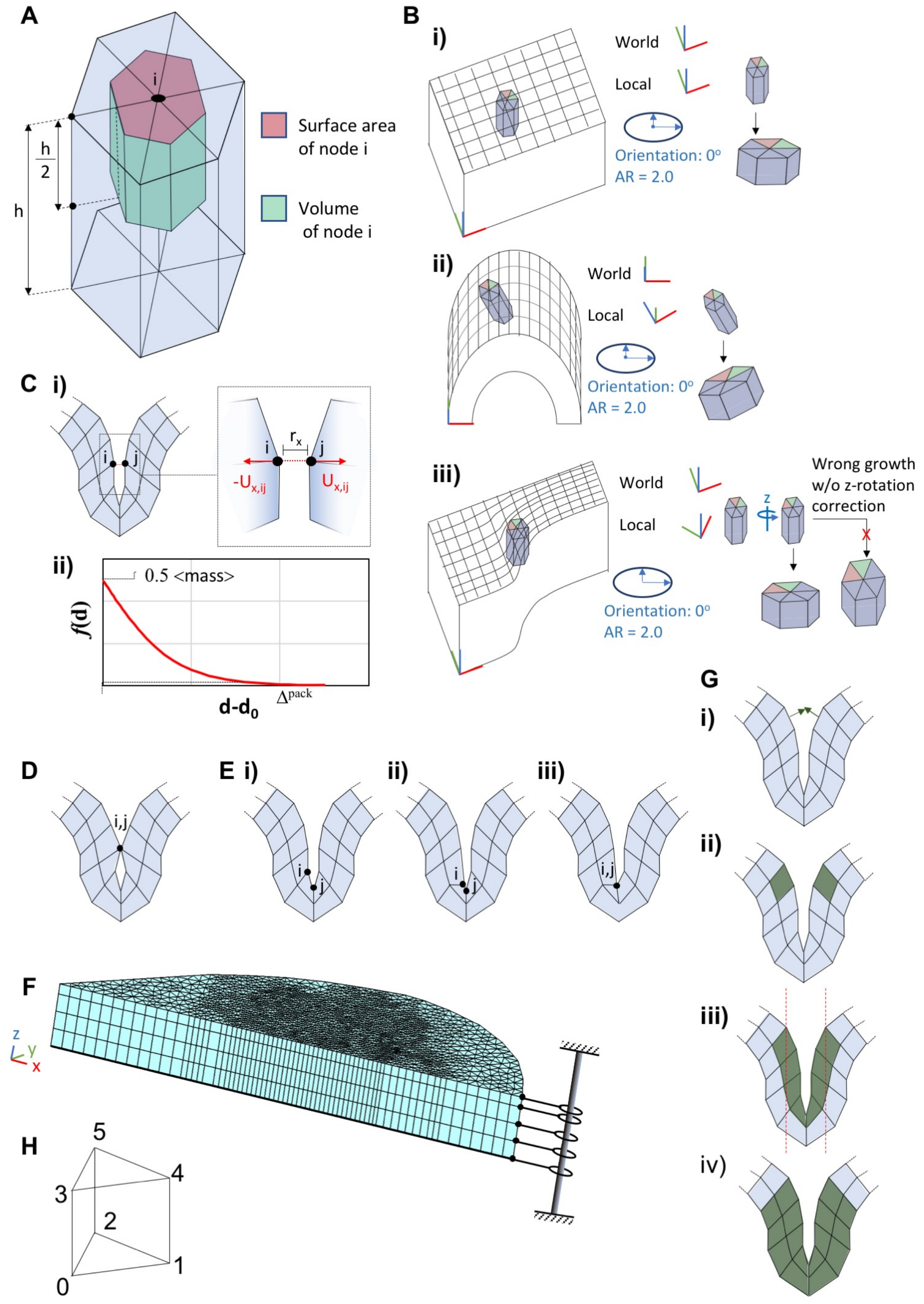

Supplementary Figure 1: Legend on next page.

Supplementary Figure 1: Related to figure 2. Modelling methodology details. A) Schematic for the externally exposed surface area and volume allocation to nodes. Node i is shared among six triangular prism elements, the total surface area highlighted in red, volume on green. B) Schematics for the oriented growth and related rotational corrections. A growth of doubling in volume, parallel to x-axis, with aspect ratio of 2 is demonstrated for all cases. All coordinate axes display x in red, y in green and z in blue. In the simulations, x is aligned with the DV axis, y with AP axis and z with AB axis of the tissue. i) Simple scenario where the world and local coordinates are aligned. ii) The elements have a rigid body rotation that deviates the local z-axis from the world coordinates, x & y are aligned. The growth follows local coordinate system, and the rigid body rotation is not accounted for. iii) The case where due to deformation in the tissue, the elements have gone through a rigid body rotation around the z-axis. The growth orientation is corrected for the rotation around z, and growth on x-y plane is applied in world coordinates. The wrong emergent grown shape in the case when this rotation was ignored is shown for comparison. C) Schematic for the hard wall potential applied to ensure volume exclusion. i) packing forces between nodes i and j in x-axis. The dashed box is enlarged on the inset, the calculated potential is applied in the opposite directions on both nodes. ii) The packing potential with distance displayed, the parameters defining the potential function are marked. D) Schematic displaying the adhesion of nodes i and j, the initial configuration same as Ci, the nodes are carried to the mid-point and their degrees of freedom bound. E) Schematic for node collapse on elements with nodes approaching within a small threshold distance of each other, implemented to limit element flipping. i) Node configuration outside collapse limit, ii) nodes moved within the collapse limit of each other due to viscoelastic system forces. iii) Configuration after node collapse. In D and E, the schematics are for demonstration purposes only and distances are not to scale. F) A sample simulated initial mesh, displaying the symmetricity assumption and showing the simulated half. Schematic added on the simulation mesh to demonstrate the no-bending boundary condition at circumference. G) Schematic demonstrating the algorithm to detect fold initiation with element surface normals. The detection is carried out on elements with exposed surfaces on either apical or basal surfaces, apical surface is utilised in demonstration. i) two normals on elements (green arrows) are within the vicinity of each other and the angle between the normals is wider than the selected threshold. ii) Two elements are assigned to be on fold initiation regions. iii) All elements that have their apical surfaces within the bounding box of the identified element couple are marked to be on fold initiation surface. For fold identification on the basal surface, the bounding box will check for basal surfaces of the remaining elements. iv) The fold initiation region is extended to cover the whole tissue thickness. H) The numbering of prism nodes in finite element formulations.

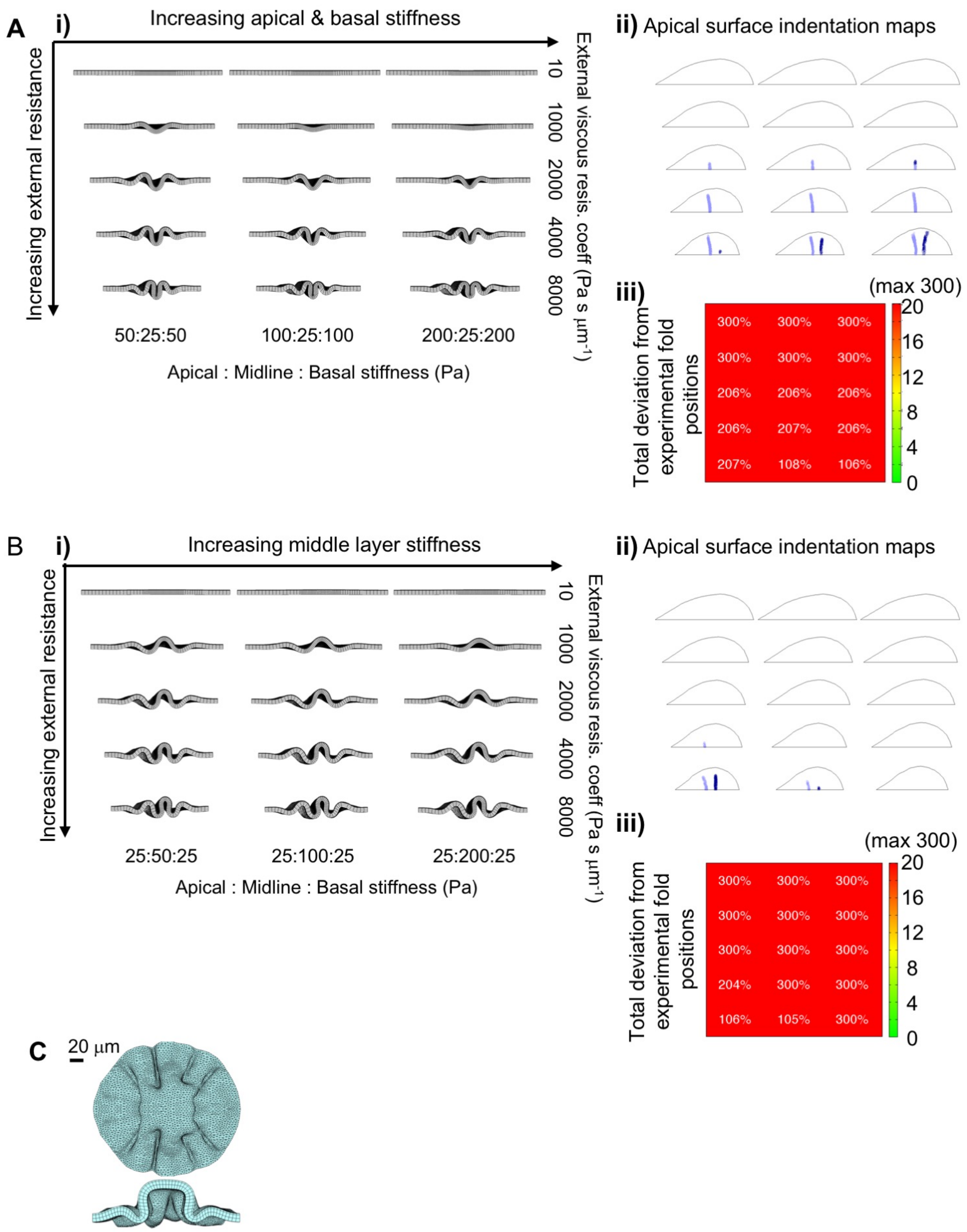

Supplementary Figure 2: Legend on next page.

Supplementary Figure 2: Related to Figure 3. Alternative relative stiffness states of the tissue. Tissue morphology with, A) increasing relative stiffness of apical and basal surfaces and external viscous resistance; B) increasing relative stiffness of tissue midline, and external viscous resistance. Each panel demonstrates simulation results for a tissue growing from 48 hour AEL to 96 hours AEL, with uniform in-plane growth rates, as stated in Figure 3A. Images are taken the cross-section of the tissue midline at 96 hours AEL, ventral tip on the right. Row and Column organisation same as Figure 3A. Both in A and B, ii) Apical indentation maps automatically identified from the curvature of facing surfaces, each continuous folding region is marked in a single colour. iii) Fold position deviations, calculated as sum of percentage deviation from each experimental fold at the tissue centre. Both i & ii calculated at same time points of (i), row column organisation is same as in (i). C) Simulation with same parameters as Figure 3B, on a symmetric, circular initial mesh. Snapshot is from 96 hours AEL, sagittal view as central cross-section, and dorsal tip to the right. Scale bar 20 μm.

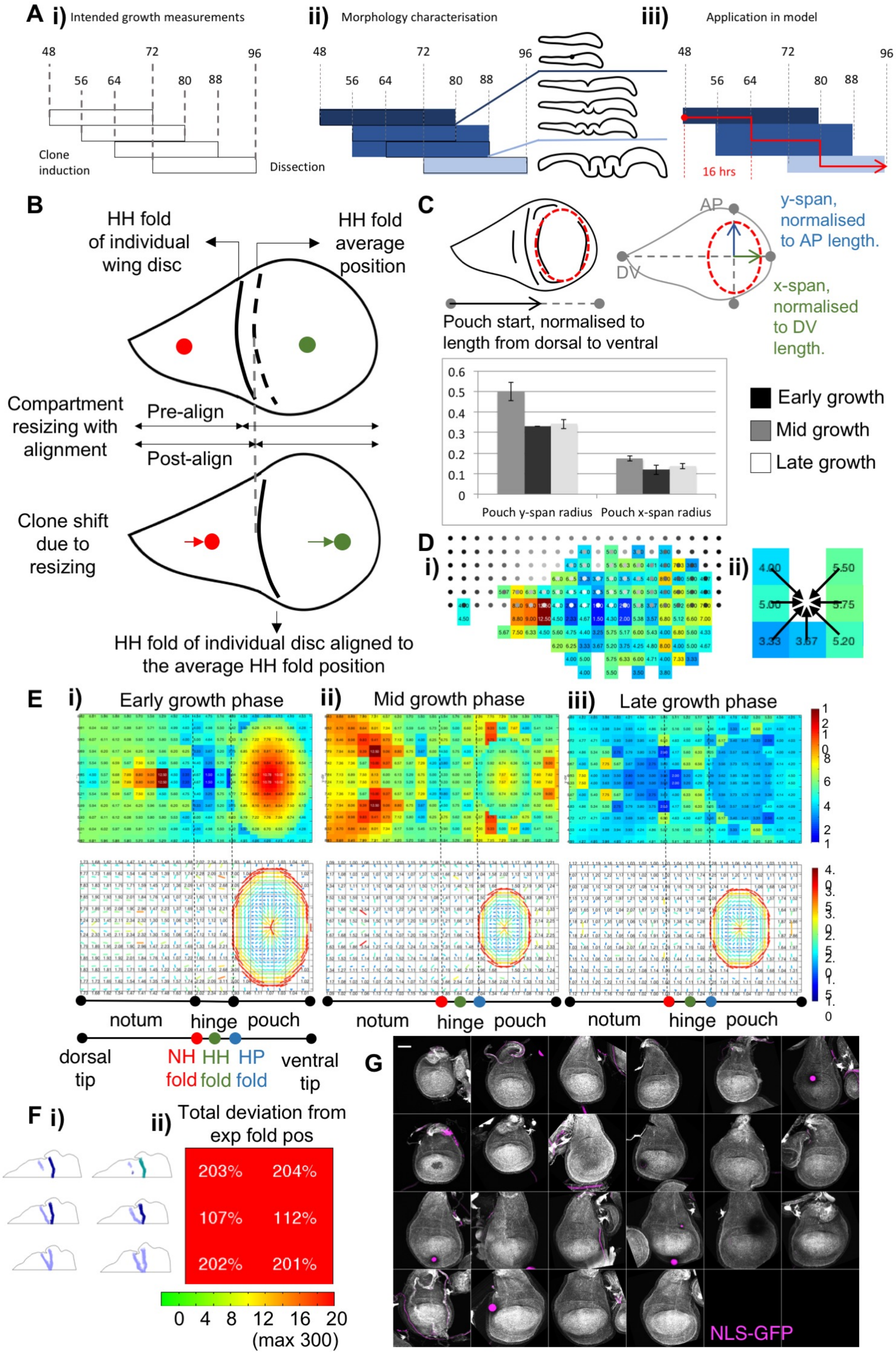

Supplementary Figure 3: Legend on next page.

Supplementary Figure 3: Related to Figure 4. Details of the growth rate analysis methodology. A) Definition of the growth phases. i) The targeted experimental timings. ii) The morphological staging of the dissected wing discs according to the folding stage. iii) The timing for the application of the growth phases in the simulations. B) Schematic representing the alignment of the growth rate measurements for a given time period. HH fold position (dashed line) is calculated as the average of all the wing-discs utilised in growth rate measurements for the selected time period. The HH position of each individual wing disc (solid line) is aligned to the average position. The two sides of the fold are rescaled to fit within the average size of the tissue, moving the data points (red on notum side, and green on pouch side) in the process. C) Measurements for the size of the pouch at different time periods within 48 to 96 hours AEL. Top schematics demonstrate how the pouch position and size are normalised. Plot demonstrates means with whiskers on one standard deviation. D) The order in which the grid points are checked, and filled as necessary. i) Order goes from light to dark shades of dots on grid points. ii) Once an empty grid point (with no experimental data points) is reached, the existing data in immediate neighbours are averaged to cover the empty point. The order of sampling for empty grid points is of significance as filled regions contribute to the filling of their neighbours, thus enabling us to fill the extended patches of empty regions on the grid. E) The extended version of growth maps demonstrated in Figure 4B. Colour bars on the right hand side of the panes are valid for all. The measured fold positions, and the corresponding tissue compartments are marked on the heatmaps, the positions are measured for 72-88 hours AEL in i&ii, for 96 hours AEL in iii. F) i) Apical indentation maps and ii) fold position deviations of simulations with experimental growth rate, as presented in Figure 4C. G) Non-heat shock controls, NLF-GFP in magenta. No spontaneous expression is observed in wing disc columnar epithelium. Scale bars 50 μm

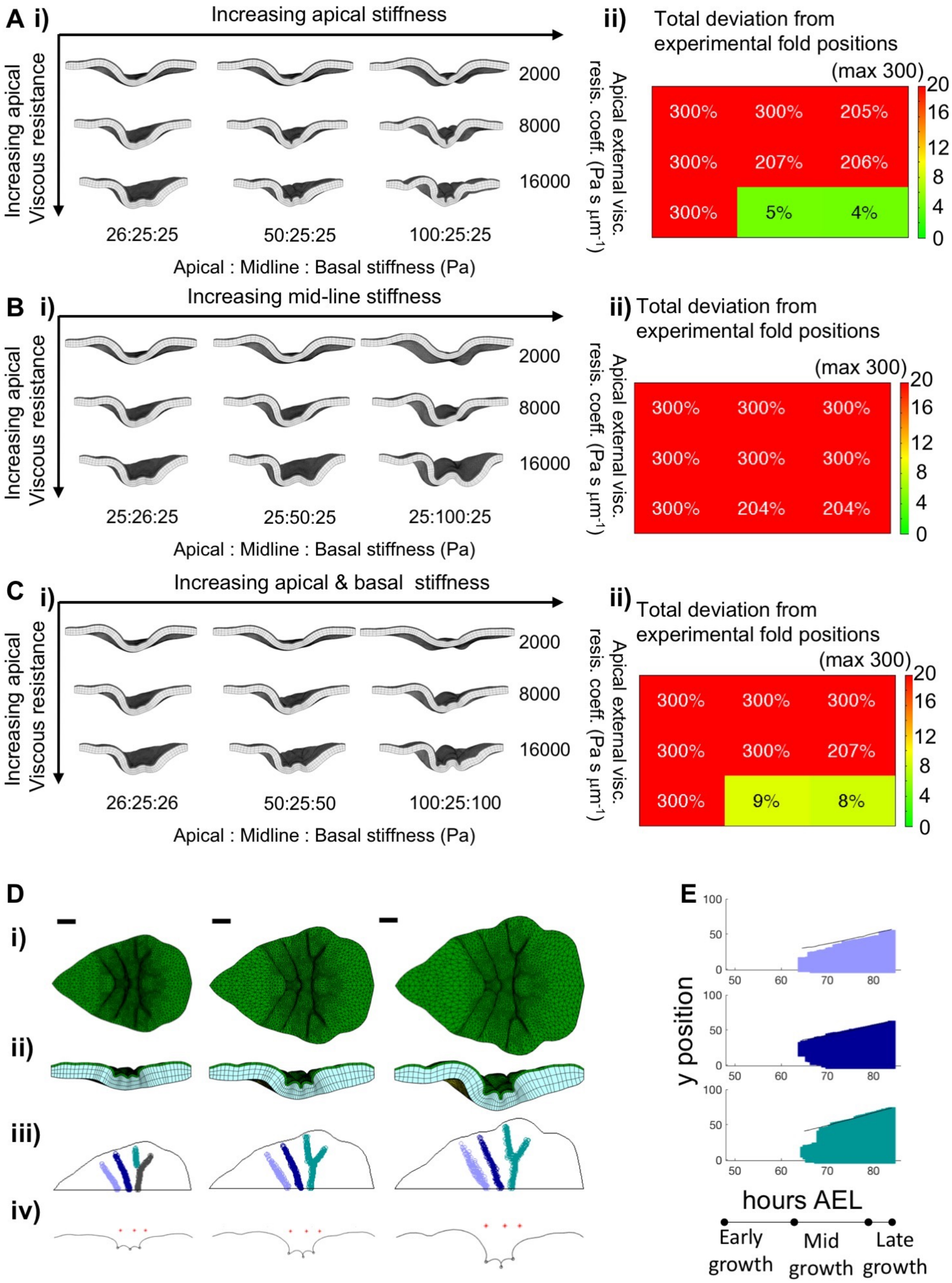

Supplementary Figure 4: Legend on next page.

Supplementary Figure 4: Related to Figure 5. A) Simulations with apical surface is stiffer than the rest of the cell body. i) The effects of apical viscous resistance coefficient and tissue stiffness heterogeneity on emergent morphology, columns: increasing apical viscous resistance coefficient, rows: increasing relative stiffness of apical surface. Simulation in lower right corner is detailed in Figure 5D. ii) Fold position deviations, calculated as sum of percentage deviation from each experimental fold at the tissue centre, row column organisation is same as in (i). The tissue has explicit BM definition at 1600 Pa stiffness and renewal half-life of 8 hour, basal viscous resistance coefficient is 10 Pa s μm^{-1}. B) Simulations with tissue midline stiffer than the rest of the tissue, grid organisation and all remaining simulation parameters are same as (A). C) Simulations with tissue apical and basal surfaces stiffer than the rest of the cell body, grid organisation and all remaining simulation parameters are same as (A). D) Timeline of z-growth added simulation in Figure 5G and Movie 5. Snapshots are from 72, 78, and 84 hours AEL, respectively. Scale bar is 20 μm. i) top view, ii) cross-section view, iii) apical indentation maps. iv) The positions of the folds on the tissue cross section apical surface profile, the red stars mark the experimental fold positions measured for 72-88 hours AEL (Fig. 1Ci). E) Kymographs of apical indentations in time. Y position of all nodes falling into the three major folds at 84 hours AEL are plotted in time. Colour coding same as (Diii) at 84 hours AEL.

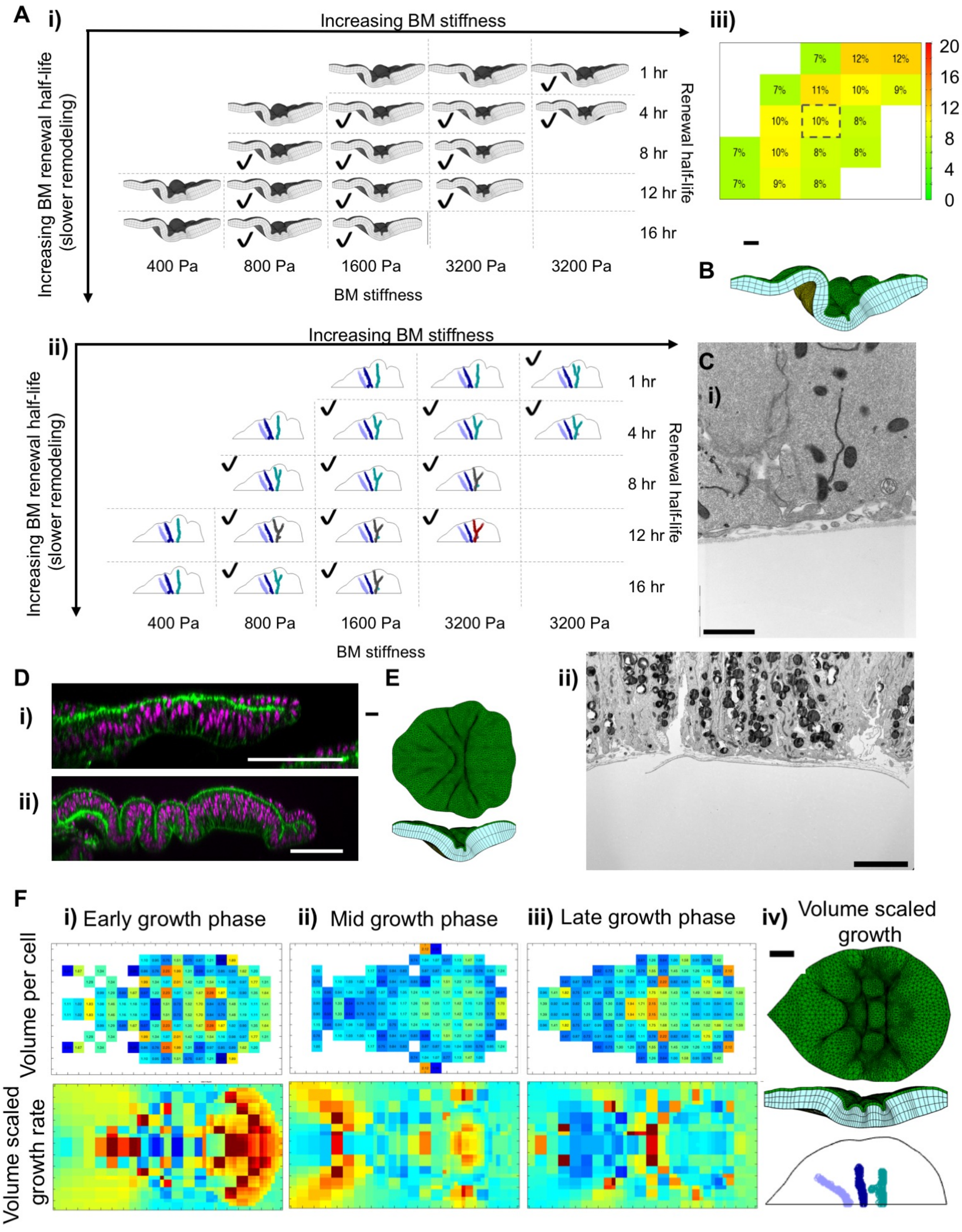

Supplementary Figure 5: Legend on next page.

Supplementary Figure 5: Related to Figure 5. A) The effects of BM stiffness and BM renewal half-life on emergent morphology. Rows, increasing BM stiffness, columns increasing renewal half-life (slower remodelling). i) Sagittal views displayed for each parameter combination at 84 hr AEL. ii) apical indentation maps. Acceptable parameter combinations marked with a green tick in (i & ii). Failing simulations form a forked HH fold, merging with the NH fold at tissue midline. iii) Percentage deviation of fold positions from the experimental positions measured at tissue midline at 36 hr AEL. The simulation presented in Figure 5G is marked by the dashed rectangle. B) The late stages of the simulation in Figure 5G, at 96 hours AEL. The initiated folds do not successfully progress into a fully folded morphology. The tissue goes through large scale buckling, the HH fold is opened up and hinge sinks well below the notum in z. Scale bar, 20 μm. C) The larger field view images of EM images (Figure 5A), i) 72 hr AEL, scale bar 1 μm, ii) 120 hr AEL, scale bar 5 μm. D) The larger field of view images where the pseudostratification images are taken from i) Figure 5Fiv-v, ii) Figure 5Fvi-vii. Scale bars 50 μm. E) Simulation on a circular initial tissue shape, with parameters same as Figure 5G. Scale bar 20 μm. F) i-iii)Normalised cell volume maps in three growth phases (top) and the growth rates scaled with the cell volume (bottom). iv) Simulation with volume scaled growth rates, snapshots are from 78 hr AEL, top panel: simulation snapshot from top view, middle: simulation snapshot from cross-section of the midline, bottom: fold initiation map of the simulation. Scale bar is 20 μm. The physical properties and boundary conditions of simulations in E/G-H are the same as presented on Figure 5D.

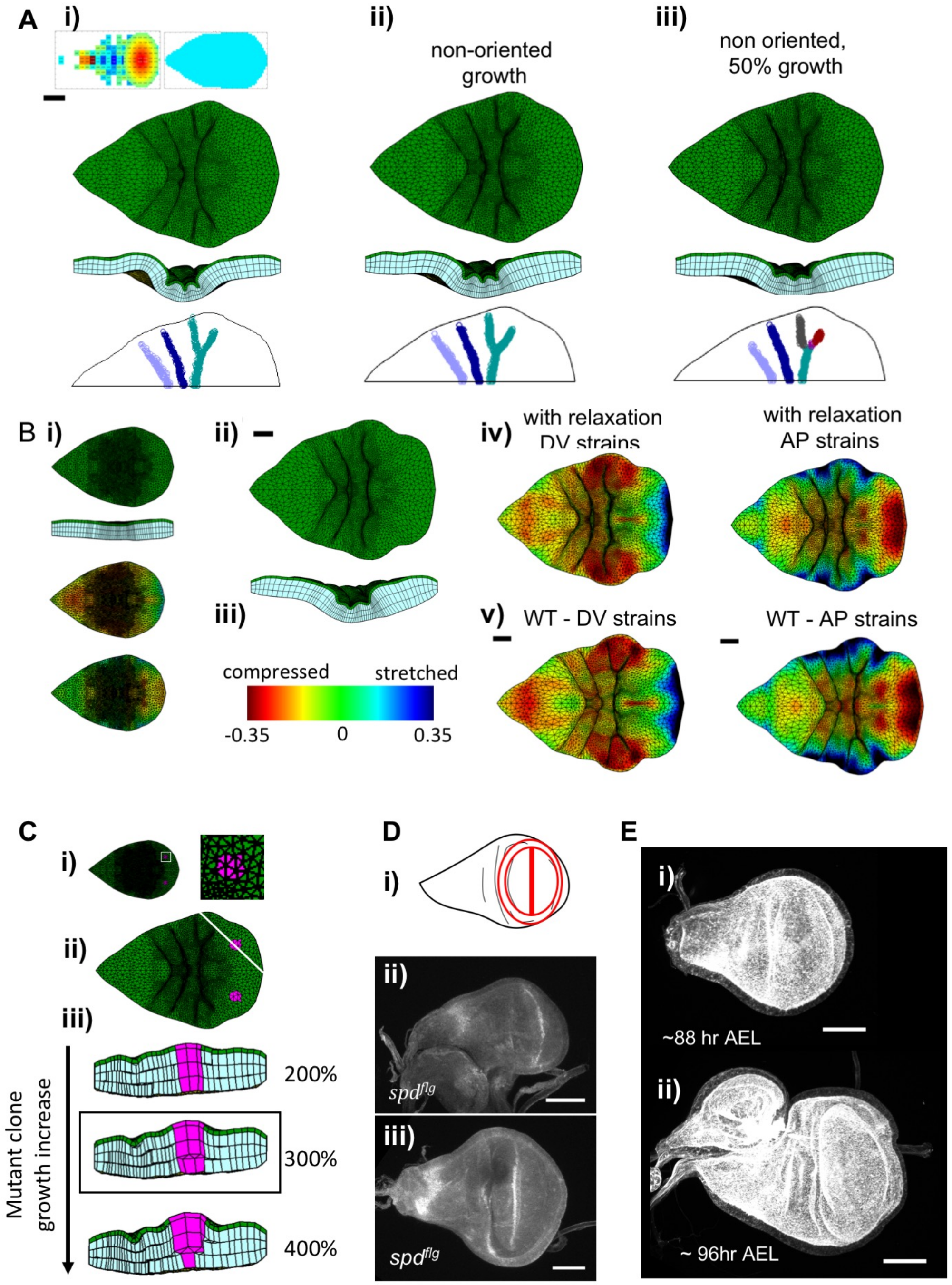

Supplementary Figure 6: Legend on next page.

Supplementary Figure 6: Related to Figure 6. The differential growth in early growth phases and related force accumulation is necessary and sufficient for correct morphology in overgrowth clones, wild type and mutant wing discs. A) i) Early growth rates and orientations applied for the initial 16 hours (48 to 64 hours AEL) of simulation, continued by uniform growth. Top panel: The growth maps applied, colour coding same as Figure 4B. Middle panel: Simulation snapshots from 84 hours AEL, top and sagittal view, scale bar 20 μm. Bottom panel: Apical indentations map at tissue at 84 hours AEL, scale same as simulation snapshots. ii) Early growth rates and orientations applied for the initial 16 hours (48 to 64 hours AEL) of simulation, continued by experimental growth rates without any orientation. Top panel: Simulation snapshots from 84 hours AEL, top and sagittal view. Bottom panel: Apical indentations map at tissue at 84 hours AEL, scale same as simulation snapshots. iii) Same as (ii), but the growth rates are also reduced to 50 percent of experimentally measured after the initial 16 hours. All simulation physical parameters are same as 5D. B) Simulation with experimental growth rates, with all accumulated forces relaxed at 58 hours AEL (10 hours into simulation). i) Top panels: Tissue morphology at 58 hours AEL, immediately prior to relaxation of forces. Middle panel: Strains accumulated in DV orientation, and bottom panel: strains on AP orientation, colour coding in (iii). ii) Simulation snapshots at 84 hours AEL, following the relaxation of forces at 58 hr AEL. iv) Strains of (ii), left: strains accumulated in DV orientation, right: strains on AP orientation, colour coding in (iii). v) Accumulated strains in wild type simulation, in Fig. 5G, panel structure and colour coding same as (iv). C) The range of tested overgrowth in clones, i) snapshot at the onset of simulation (48 hr AEL), with the induced clone (magenta) within white rectangle enlarged on the right. Clone diameter approximately 4 micrometers. ii) Simulation snapshot, top view from 75 hr AEL, for 300 percent growth in clone (corresponding to Fig. 6D). The while line marks the line of cross-section represented in (iii). iii) Simulation snapshots demonstrating ectopic fold emergence as the overgrowth is increased. The setup demonstrated in Figure 6 B is boxed. Simulation snapshots from 75 hr AEL unless stated otherwise on the image, all physical properties same as Fig. 5D. D) i) Schematic marking the pattern of *wingless* expression in wild type wing discs. The inner ring appears prior to fold formation in early third instar, followed by the outer ring in late third instar. ii) *Wingless* staining in spd^{fg}, prior to formation of folds, the stage where the inner ring should have appeared. iii) Wingless staining in late third instar mutant wing disc, showing the outer ring of expression, and lacking the inner ring. Scale bars 50 μm. E) The top views maximum projection images of the experimental spd^{fg} mutants shown in Figure 6I, Scale bars 50 μm

Method S1: Methodology for the computational model, related to STAR Methods

1 Introduction

This methods section describes our finite element model of tissue morphogenesis. In our model, the tissue is treated as a continuous material. The discretisation is based on 6-node prism elements, that can grow and vary in size from subcellular to multicellular (Fig. 2A-C, S1A). The driving force behind the shape change of the tissue is this growth of the elements. Emergent nodal positions, thus the tissue morphology, is governed by the balance of the elastic forces and external viscous resistance to movement. The discrete form of balance equations give rise to a system of non-linear equations, that are linearised and solved numerically with a Newton-Raphson method.

The stress-strain relationship of the tissue is represented by a Neo-Hookean material model. The sub-compartments of the tissue utilise different set of parameters, but satisfy the same governing equations, for example the basement membrane has relatively higher stiffness. When applied, the external viscous resistance is proportional to the exposed surface area and velocity. The parameters utilised through the manuscript are summarised in Supplementary Table 3.

The initial geometry is defined by tessellation of the contour of a wing disc at 48 hours AEL using the Trianlge software (Shewchuk, 2005). The contour is scaled to the average dimensions (Fig. 1C, 2E). We assume anterior-posterior symmetry and simulate half of the tissue. As such, the simulations are run with fixed y-position (along AP axis) for all nodes at the dorsal-ventral plane of symmetry, that is at the midline of the tissue. The outer boundary conditions at the circumference of the tissue limit bending, such that all nodes on the same column at the boundary have the same x & y coordinates (Fig. S1F). During simulations, the tissue surface can form adhesions. Volume exclusion is ensured by a hard-wall potential and element flipping is avoided by collapsing nodes on single elements when the edge of the element is below a set threshold (Fig. S1C-E).

2 Modelling methodology

2.1 Equilibrium equation and finite element discretisation

The tissue is modelled as a deformable body occupying a time varying domain Ω_t. We denote by $\boldsymbol{x}(t)$ the current positions of the material point that is initially at position $\boldsymbol{X}$. After neglecting body loads (gravity) and inertial terms, the equilibrium equations of Ω_t are given by Cauchy's equations and appropriate boundary conditions:

$$\begin{aligned} \nabla \cdot \boldsymbol{\sigma} &= \boldsymbol{0},\ \forall \boldsymbol{x} \in int(\Omega_t) \\ \boldsymbol{\sigma n} &= \bar{\boldsymbol{t}},\ \forall \boldsymbol{x} \in \Gamma_n \\ \boldsymbol{x} &= \bar{\boldsymbol{x}}\ \forall \boldsymbol{x} \in \Gamma_x, \end{aligned} \tag{1}$$

with $\bar{\boldsymbol{t}}$ and $\bar{\boldsymbol{x}}$ prescribed loads and positions at boundaries Γ_n and Γ_x, respectively, which will specified later, and $\boldsymbol{n}$ the external normal.

The finite element formulation is obtained by a standard construction of the equivalent weak form (Bonet and Wood, 2008), which reads,

$$\int_{\Omega_t} \boldsymbol{d}(\delta\boldsymbol{v}) : \boldsymbol{\sigma} dV_t = \int_{\Gamma_n} \delta\boldsymbol{v} \cdot \bar{\boldsymbol{t}} dS_t, \forall \delta\boldsymbol{v} \in H_0 \tag{2}$$

The vector $\delta\boldsymbol{v}$ is a test function belonging to appropriate Hilbert space H_0 of functions with bounded integrals, and the strain tensor $\boldsymbol{d}(\delta\boldsymbol{v}) = \frac{1}{2}\left(\nabla_x \delta\boldsymbol{v} + (\nabla_x \delta\boldsymbol{v})^T\right)$ is a measure of the body deformation.

The finite element discretisation is achieved by introducing an interpolation of the positions $\boldsymbol{x}(t) \approx N_j(\boldsymbol{x})\boldsymbol{x}_j(t)$ and the test functions $\delta\boldsymbol{v} \approx N_i(\boldsymbol{x})\delta\boldsymbol{v}_i$ with a set of complete shape functions $N_i(\boldsymbol{x})$ (Supplementary Table 1) and where $\boldsymbol{x}_j(t)$ are time varying nodal positions. In our case, we use six noded elements forming a triangular prism aligned along the apical-basal axis (see Figure S1H), so that positions are interpolated linearly along each element height, and bi-liniearly at each element cross-section. Then within each element, a numerical approximation is carried out with six Gauss points quadrature (Supplementary Table 2).

The imposition of the discretised version of the weak form in (2) leads to the following system of equations (see Bonet and Wood, 2008 for a detailed derivation):

$$\boldsymbol{g}(\boldsymbol{x}, t) \equiv \boldsymbol{g}^{elast}(\boldsymbol{x}, t) - \boldsymbol{g}^{ext}(\boldsymbol{x}, t) = \boldsymbol{0} \tag{3}$$

where for each node i, the elastic and external residual contributions are given by,

$$\boldsymbol{g}_i^{elast} = \int_{\Omega_t} \boldsymbol{\sigma} \nabla_x N_i dV_t \tag{4}$$

$$\boldsymbol{g}_i^{ext} = \int_{\Omega_t} N_i \bar{\boldsymbol{t}} dS_t \tag{5}$$

Here, and in the subsequent derivations, we use $\boldsymbol{x}$ to represent $\boldsymbol{x}(t)$ for clarity. This nodal contributions are computed element-wise and assembled in the standard manner in the finite element context (Bonet and Wood, 2008).

As it will be described below, the stress tensor $\boldsymbol{\sigma}$ follows a non-linear constitutive law that varies along time, while the external forces in $\boldsymbol{g}^{ext}$ includes time dependent viscous forces. As a result, and due to the presence of large deformations, the set of non-linear equations in (3) is discretised in time using a backward Euler implicit scheme and solved iteratively at each time-step with a Newton-Raphson process in order to achieve quadratic convergence. This process requires the linearisation of the residual $\boldsymbol{g}(\boldsymbol{x}_n, t_n)$ at each time-step t_n. At iteration k, the new iterative changes of the displacements $\delta\boldsymbol{x}$ are found by solving the following linear equations:

$$\boldsymbol{g}(\boldsymbol{x}_n^k, t_n) + \boldsymbol{K}\delta\boldsymbol{x} = \boldsymbol{0} \ \rightarrow \ \boldsymbol{x}_n^{k+1} = \boldsymbol{x}_n^k + \delta\boldsymbol{x}. \tag{6}$$

The Jacobian $\boldsymbol{K} = \frac{\partial \boldsymbol{g}}{\partial \boldsymbol{x}}$ is also updated at each iteration from $\boldsymbol{x}_n^k$ and $\boldsymbol{x}_{n-1}$. Its expression will be specified below.

2.2 Deformation gradient decomposition and elastic constitutive law

The elastic constitutive law requires the computation of the total deformation gradient $\boldsymbol{F} = \frac{\partial \boldsymbol{x}}{\partial \boldsymbol{X}}$, which is decomposed into elastic and growth components,

$$\boldsymbol{F} = \boldsymbol{F}^e \boldsymbol{F}^g \tag{7}$$

The stress tensor $\boldsymbol{\sigma}$ is assumed to follow a compressible Neo-Hookean non-linear constitutive law that solely depends on the elastic component $\boldsymbol{F}^e$ through an elastic density function (Bonet and Wood, 2008)

$$W(\boldsymbol{C}^e) = \frac{\mu}{2}(trace(\boldsymbol{C}^e) - 3) - \mu \ln J^e + \frac{\lambda}{2}(\ln J^e)^2 \tag{8}$$

with λ and μ constant material parameters, $\boldsymbol{C}^e = \boldsymbol{F}^{eT}\boldsymbol{F}^e$ the right Cauchy-Green strain tensor, and $J^e = \det(\boldsymbol{F}^e) = \sqrt{\det(\boldsymbol{C}^e)}$. The Cauchy stress is then given by

$$\boldsymbol{\sigma} = J^{e-1}\boldsymbol{F}^e \boldsymbol{S} \boldsymbol{F}^{eT} \tag{9}$$

with $\boldsymbol{S} = 2\frac{\partial W(\boldsymbol{C}^e)}{\partial \boldsymbol{C}^e}$ the second Piola-Kirchhoff stress tensor:

$$\boldsymbol{S}^e = \mu(\mathbf{I} - \mathbf{C}^{-1}) + \lambda(lnJ^e)\mathbf{C}^{-1} \tag{10}$$

Note that the computation of the stresses from the current deformation $\boldsymbol{F}$ depends on the growth component $\boldsymbol{F}^g$, which as will shown below is time dependent.

From the expression of $\boldsymbol{g}_i^{elas}$ in equation (4) and the definition of the elasticity function and stresses through equations (8)-(9), the following contribution to the Jacobian, coupling nodes i and j can be derived

$$\mathbf{K}_{ij}^{elast} = \frac{\partial \boldsymbol{g}_i^{elast}}{\partial \boldsymbol{x}_j} = \int_V \boldsymbol{B}_i^T \boldsymbol{F}\{\mathcal{C}\}\boldsymbol{F}^T \boldsymbol{B}_j dV + \mathbf{I}\int_V \nabla_x N_i^T \boldsymbol{\sigma} \nabla_x N_j dV. \tag{11}$$

with $\boldsymbol{B}_i$ a deformation matrix that allows to compute the deformation rate as $\boldsymbol{d}(\delta\boldsymbol{x}) = \boldsymbol{B}_i \delta \boldsymbol{x}_i$, and $\{\mathcal{C}\}$ the matrix notation of the fourth order elasticity tensor $\mathcal{C}$,

$$\mathcal{C}_{ijkl} = \lambda \boldsymbol{C}_{ij}^{-1}\boldsymbol{C}_{kl}^{-1} + 2\left(\mu - \lambda ln(J)\right)\mathcal{I}_{ijkl} \tag{12}$$

where $\mathcal{I}_{ijkl}$ is given by

$$\mathcal{I}_{ijkl} = \frac{1}{2}\left[\boldsymbol{C}_{ik}^{-1}\boldsymbol{C}_{jl}^{-1} + \boldsymbol{C}_{il}^{-1}\boldsymbol{C}_{jk}^{-1}\right]. \tag{13}$$

2.3 External force contribution

We assume that the external forces at the boundary Γ_n correspond to a viscous loading due to friction with the external environment. The nodal drag forces are consequently given by

$$\boldsymbol{g}_i^{ext} = \int_{\Gamma_n} \eta_{ext} N_i \boldsymbol{v} dS_t$$

with η_{ext} the external viscous resistance coefficient, and $\boldsymbol{v}$ the velocity at the boundary, the boundary domain being the externally exposed apical and basal surfaces of the tissue..

This resistance is defined on a nodal basis and is heterogeneous within different regions of the tissue, having most significant influence on the dynamics of the apical surface (Supplementary Table 3). Both the velocity and the boundary Γ_n are time-dependent. In order to reduce the computations and the linearisation needed for the Newton-Rapshon solution, the nodal contribution is approximated at time t_n as

$$\boldsymbol{g}_{i,n}^{ext} = \int_{\Gamma_n} \eta_{ext} N_i \boldsymbol{v} dS_t \approx \eta_{ext} A_{n-1,i} \frac{\boldsymbol{x}_n - \boldsymbol{x}_{n-1}}{\Delta t}$$

with $\Delta t = t_n - t_{n-1}$ the time-step size, and $A_{n-1,i}$ the area attributed to node i at time t_{n-1}, which is computed as,

$$A_{n-1,i} = \sum_{e=1:n_{owner}} \frac{A_{n-1,e}}{n_{e,surf}} \tag{14}$$

Here, n_{owner} is the number of elements connected to node i, $A_{n-1,e}$ is the exposed surface area of interest on element e at time t_{n-1}, and $n_{e,surf}$ is the number of nodes that element e has on its exposed surface of interest (Fig. S1A). For instance, for prisms of 6 nodes, the number of nodes associated with the apical surface would be 3. From the expression of $\boldsymbol{g}_i^{ext}$, the following contribution of the external forces to the Jacobian can be deduced:

$$\boldsymbol{K}_{ij}^{ext} = \frac{\eta_{ext} A_{n-1,i}}{\Delta t} \mathbf{I}.$$

2.4 Calculation of growth

The growth rates are input to the simulations in the form of the experimental growth maps of Figure 4. To obtain the local growth rate of a single element, first the relative position of the element centre in the xy-plane bounding box of the tissue is calculated. Then the growth rate, $[r_x, r_y, r_z]$, and the growth orientation angle, θ_g, is interpolated from the nearest corners of the input growth map grid (Figure 2 for experimental measurements). This growth is then incorporated into the element by a multiplicative decomposition in equation (7): $\boldsymbol{F} = \boldsymbol{F}^e \boldsymbol{F}^g$ (see Fig. 2B). Consequently, $\boldsymbol{F}^g$ must be updated at each time step Δt, depending on the input growth rates, growth orientation, and the current rigid body rotations of the element according to,

$$\boldsymbol{F}_{t+\Delta t}^g = \boldsymbol{R}_{er} \boldsymbol{R}_{gr} \begin{bmatrix} e^{r_x \Delta t} & 0 & 0 \\ 0 & e^{r_y \Delta t} & 0 \\ 0 & 0 & e^{r_z \Delta t} \end{bmatrix} \boldsymbol{R}_{gr}^T \boldsymbol{R}_{er}^T \boldsymbol{F}_t^g. \tag{15}$$

Here the growth rates are in the local coordinates of the element, $\boldsymbol{R}_{gr}$ is the rotation matrix for the growth orientation angle (θ_g) and $\boldsymbol{R}_{er}$ is the rotation matrix associated with the current elemental rotation in the plane of the tissue. $\boldsymbol{R}_{gr}$ is simply the rotation around the z-axis with the input growth orientation angle θ_g.

This growth orientation angle θ_g in simulation inputs is calculated from the maximum projection of the experimental images on the xy-plane. To match this methodology, the current rotation of the element around the z-axis is corrected in order to ensure that the orientation of the growth follows the xy-plane of the tissue. At the beginning of the simulations, the local coordinate system of each element is aligned with the world coordinate

system. During the simulation, the local coordinate system of the element could deviate from the world coordinates, due to rigid body rotations, imposed by the deformations of the surrounding tissue (Fig. S1B). Any rotation around the z-axis, changing the xy-plane of the element, should be accounted for, so that the element will continue growing on the desired orientation in the world coordinates. On the other hand, the tilt of the z-axis itself should be ignored; an element with tilted apical-basal (AB) axis should not start elongating in the AB direction (Fig. S1B). To obtain the rotation matrix $\boldsymbol{R}_{er}$ first the current rigid body rotation of the element is calculated from the deformation gradient via single value decomposition such that:

$$\boldsymbol{F} = \boldsymbol{V}\boldsymbol{P}\boldsymbol{U}^{T}. \tag{16}$$

Then the rotation matrix corresponding to the rigid body rotation of the deformed element can be obtained from:

$$\boldsymbol{R}_{rigid} = \boldsymbol{V}\boldsymbol{U}^{T} \tag{17}$$

and the angle of rotation around the z axis is extracted from the calculated rigid body rotation matrix from:

$$\theta_z = \arctan\left(\boldsymbol{R}_{rigid}\left(0,1\right), \boldsymbol{R}_{rigid}\left(0,0\right)\right) \tag{18}$$

where

$$\boldsymbol{R}_{rigid} = \boldsymbol{R}_x\left(\theta_x\right)\boldsymbol{R}_y\left(\theta_y\right)\boldsymbol{R}_z\left(\theta_z\right). \tag{19}$$

The elemental rotation matrix $\boldsymbol{R}_{er}$ corrects for the rotation in z, as such, $\boldsymbol{R}_{er}$ is rotation around z axis by $-\theta_z$.

2.5 Calculation of remodelling

While the cellular elements of the tissue grow with specified growth rates and orientations, the basal membrane (BM) grows by remodelling. The application of remodelling follows the logic of equation (15), with the growth increment and related rotations defined from deformations, rather than an input growth profile. As such, the rigid body rotation correction, $\boldsymbol{R}_{er}$, is the identity matrix for BM remodelling, and the equivalent of growth orientation, $\boldsymbol{R}_{gr}$, is obtained through the elastic deformation orientation. In the generalised definition, the remodelling growth at each time step is obtained via Eigen value decomposition of the Cauchy-Green strain matrix $\boldsymbol{E} = \frac{1}{2}\left(\boldsymbol{C} - \boldsymbol{I}\right)$ of the element, and the deformations on the principal axes are calculated via equation,

$$F_{kk} = \sqrt{2e_k + 1} \tag{20}$$

Here, F_{kk} is the current deformation along the principal axis k, (such that a 50% stretch will give a value of 1.5), and e_k is the kth Eigen value of $\boldsymbol{E}$. In the specific case of basement membrane remodelling, as the BM is stretched there is no evidence that the BM should be getting thinner. On the contrary, BM does get thicker with age as demonstrated in our quantification of EM images of wing disc BMs at 72 and 120 hr AEL (Figure 5A). Moreover, as the remodelling in the simulation is based on the strains on the elements, while the BM is deformed and the new BM is allocated, the BM thickness should not be

reduced with remodelling. As such, remodelling is limited to plane of the tissue. BM is not remodelled in the apical-basal axis, only the x & y dimensions of the strain matrix are included in the decomposition. Here, the range of k in equations (20) - (22) are limited to $k = 1 : 2$ in 2D, excluding local z coordinates of the element, which is aligned with apical-basal axis. Similarly, the Eigen value decomposition is also carried out on the x-y plane of the Cauchy-Green strain matrix $\boldsymbol{E}$. The deformation after the relaxation to be observed within the current time step Δt is then calculated from the given remodelling half-life, $t_{1/2}$ as follows:

$$F_{kk}^{t+\Delta t} = \left(F_{kk}^{t} - 1\right)\left(\frac{1}{2}\right)^{\Delta t/t_{1/2}} \tag{21}$$

The calculated new deformation is then converted to a growth increment, with the orientation of the growth defined by the eigenvectors matrix $\boldsymbol{V}_{Eigen}$ to give the remodelling deformation gradient.

$$\boldsymbol{F}^{r} = \boldsymbol{V}_{eigen} \begin{bmatrix} F_{11}^{t+\Delta t}/F_{11}^{t} & 0 & 0 \\ 0 & F_{22}^{t+\Delta t}/F_{22}^{t} & 0 \\ 0 & 0 & 1 \end{bmatrix} \boldsymbol{V}_{eigen}^{T} \tag{22}$$

The remodelling serves to mimic BM remodelling carried out by the cells as the tissue grows, adding or removing material from the BM layer as needed. Therefore, remodelling of BM is done without volume conservation (determinant of $\boldsymbol{F}_r$ can deviate from unity). At each time step, the remodelling growth increment $\boldsymbol{F}_r$ is added to total growth deformation gradient $\boldsymbol{F}^g$,

$$\boldsymbol{F}^{g} = \boldsymbol{F}^{r}\boldsymbol{F}^{g'} \tag{23}$$

where $\boldsymbol{F}^{g'}$ is the growth increment of the current step prior to remodelling as calculated through equation (15). For a simplified visual representation of the emergent remodelling, the relaxation of deformation is represented in a 2D schematic in Figure 5C, where upon change of the current shape of an element (blue square, Fig. 5Ci-ii), the preferred shape (red dashed square) resulting from application of remodelling on reference shape gradually changes and aligns with the current shape (Fig 5Cii-iii), relaxing the strains in the process (Fig. 5Civ).

2.6 Node-node interactions

Nodes can interact with each other via a packing hard-wall potential to simulate tissue self-contact, or through adhesions. When the two surfaces of the tissue are in close vicinity, they form adhesions, that is, nodes are joined together and are collapsed to a single node. Packing forces are used instead for cases where adhesion between the nodes would cause the shared element to flip. In cases where the nodes of an element are approaching too close to each other, such as the case of a highly constrained apical surface at a fold, the nodes of the element are also collapsed, in order to avoid element flipping.

2.6.1 Calculation of packing hard-wall potential

The hard wall potential is defined to simulate contact and ensure volume exclusion as the elements move too close to each other (Fig. S1C). The potential is applied on a nodal

basis. The threshold of repulsion force application, Δ^{pack}, is dynamic in the simulations, scaling to the average side length of an element in the vicinity of potential node-node interaction. The threshold in the simulations throughout the manuscript is defined to be 40% of the average local side length, calculated on a 10 by 5 grid on the tissue xy bounding box. The parameters of the hard-wall potential are selected numerically in order to ensure volume exclusion and stability, not necessitating a biological basis, the values are presented in Supplementary Table 3.

The magnitude $f(d)$ of the applied packing force is a function of the distance d between nodes, and is calculated with an inverse logic function:

$$f\left(d\right) = \frac{\langle mass \rangle}{1 + e^{-k(d-d_0)}} \tag{24}$$

Here, the amplitude $\langle mass \rangle$ is defined such that the force will scale with the average mass of the packing node couple. The mass itself is taken proportional to the volume associated to each node. The slope of the curve in the force profile will tend to zero as the distance between nodes approaches the packing threshold Δ^{pack}. Parameter k of the sigmoid function in (24) is defined by the saturation term s^{sat} and the packing threshold Δ^{pack} as

$$-k = \frac{2\, s^{sat}}{\Delta^{pack}} \tag{25}$$

The packing threshold Δ^{pack} is dependent on the current average side lengths of mesh elements. The sigmoid saturation is set to 5, as this is the approximate saturation distance of the standard logistic function. The distance is shifted with distance d_0 to move the mid point of the function to approximately 60% of the packing threshold distance. The forces between each pair of nodes i and j is computed from $f(d)$ as,

$$\boldsymbol{f}_i(d) = f(d)\,\boldsymbol{e}_i\ ,\ \ \boldsymbol{f}_j(d) = f(d)\,\boldsymbol{e}_j = -f(d)\,\boldsymbol{e}_i = -\boldsymbol{f}_i \tag{26}$$

with $d = ||\boldsymbol{x}_i - \boldsymbol{x}_j||$ and $\boldsymbol{e}_i = \left(\boldsymbol{x}_i - \boldsymbol{x}_j\right)/d$. The forces $\boldsymbol{f}_i$ and $\boldsymbol{f}_j$ are added to the global residual vector $\boldsymbol{g}$ in (3), and the term

$$\frac{\partial \boldsymbol{f}_i}{\partial \boldsymbol{x}_j} = \frac{f(d)}{d}\left(\mathbf{I} - \boldsymbol{e}_i\boldsymbol{e}_j^T\right) + f'(d)\boldsymbol{e}_i\boldsymbol{e}_j^T,$$

with $f'(d) = -2\,\langle mass \rangle\, s^{sat} f(d)(1 - f(d))/\Delta^{pack}$, is also added to the corresponding term $\boldsymbol{K}_{ij}$ in the Jacobian during the Newton-Raphson solution process.

2.6.2 Calculation of node binding for node adhesion, elemental collapse and boundary conditions

Nodes within a close vicinity can bind to each other (Fig. S1D). This can be due to adhesion of two surfaces in the case of nodes that are not shared among any elements, or due to the collapse of an elemental surface. Adhesion is defined by moving both nodes to the mid-point, and collapsing their degrees of freedom. The two nodes are assigned master and slave status arbitrarily. All the driving and drag forces of the slave node are carried on to the master node, and the Jacobian is updated accordingly. Upon obtaining the displacements, the displacement of the slave node is updated with the displacement

of the master node. This is equivalent to a master-slave treatment of the nodal constraint $\boldsymbol{x}_{slave} = \boldsymbol{x}_{master}$ (see for instance (Muñoz, J.J. and Jelenić, 2004).

The distance threshold for adhesion is calculated the same manner as the hard wall potential. The threshold for element collapse is stricter than adhesion; defined on an elemental basis, as a distance below 10% of the initial reference length between the two potentially collapsing nodes, as opposed to being proportional to the current average side length of the system (Fig. S2E). The residual nodal forces $\boldsymbol{g}$ and Jacobian $\boldsymbol{K}$ are then modified as follows:

$$\boldsymbol{K}_{bound} = \boldsymbol{N}^T\boldsymbol{K}\boldsymbol{N} + \bar{\boldsymbol{I}} \tag{27}$$

$$\boldsymbol{g}_{bound} = \boldsymbol{N}^{\boldsymbol{T}}\boldsymbol{g} \tag{28}$$

$$\boldsymbol{N}_{ij} = \begin{cases} 1 \quad if \quad i = j \text{ and } i \text{ is not a slave} \\ 1 \quad if \qquad i \text{ is slave to } j \\ 0 \qquad\qquad \text{elsewhere} \end{cases} \tag{29}$$

$$\bar{\boldsymbol{I}}_{ij} = \begin{cases} 1 \quad if \quad i = j \text{ and } i \text{ is a slave} \\ 0 \qquad\qquad \text{elsewhere} \end{cases} \tag{30}$$

The same master slave definition is used for no-bending boundary condition. At the circumference, the basal node of each column of nodes is assigned as the master of all the remaining nodes of the column, and the degrees of freedom in x and y directions of slaves are fixed on the master (Fig. S1F).

2.7 A beginners guide to implementation of the linearised form of elastic stresses and the stiffness matrix

2.7.1 Nodal definition and shape functions of a prism

For a triangular prism, the number of nodes, n, is 6, the numbering starting from the bottom three nodes (Fig. S1F). The finite element modelling discretisation using Lagrangian interpolation on a nodes can be carried through shape functions N. These shape functions and their derivatives in the parametric coordinates η, ζ and ν are given in Table 1 for a six point discretisation of the prism element. The numerical calculation for nodal forces ($\boldsymbol{g}$) and the derivatives of the forces with respect to nodal movements ($\boldsymbol{K}$) are carried out at six Gauss points, with the parametric coordinates and weights as given in Table 2.

2.7.2 Calculation of deformation gradient $\boldsymbol{F}$

The deformation gradient can be represented in the form of derivatives of current and reference coordinates in the parametric coordinates:

$$\boldsymbol{F} = \frac{\partial \boldsymbol{x}}{\partial \boldsymbol{X}} = \frac{\partial \boldsymbol{x}}{\partial \boldsymbol{\xi}} \left(\frac{\partial \boldsymbol{X}}{\partial \boldsymbol{\xi}} \right)^{-1} \tag{31}$$

By the definition of shape functions $\boldsymbol{x} = \sum_{i=1}^{n} N_i x_i$, $\partial \boldsymbol{x}/\partial \boldsymbol{\xi}$ can be obtained from the shape function derivatives with n=6 for the current prism definition and the nodal data in Table 1:

$\lambda = 1 - \xi - \eta$ $\alpha = (1-\zeta)/2$ $b = (1+\zeta)/2$				
Node	Shape function. N	Shape function derivative wrt ξ $\frac{\partial N}{\xi}$	Shape function derivative wrt ζ $\frac{\partial N}{\zeta}$	Shape function. derivative wrt η $\frac{\partial N}{\eta}$
1	$\lambda\alpha$	$-\alpha$	$-\alpha$	$\frac{-\lambda}{2}$
2	$\xi\alpha$	α	0	$\frac{-\xi}{2}$
3	$\eta\alpha$	0	α	$\frac{-\nu}{2}$
4	λb	$-b$	$-b$	$\frac{\lambda}{2}$
5	ξb	b	0	$\frac{\xi}{2}$
6	ηb	0	b	$\frac{\eta}{2}$

Supplementary Table 1: Shape functions and derivatives of prism element. Related to STAR methods

Gauss point	ξ	ζ	η	weight
1	$\frac{1}{6}$	$\frac{1}{6}$	$\frac{1}{\sqrt{3}}$	$\frac{1}{6}$
2	$\frac{2}{3}$	$\frac{1}{6}$	$\frac{1}{\sqrt{3}}$	$\frac{1}{6}$
3	$\frac{1}{6}$	$\frac{2}{3}$	$\frac{1}{\sqrt{3}}$	$\frac{1}{6}$
4	$\frac{1}{6}$	$\frac{1}{6}$	$-\frac{1}{\sqrt{3}}$	$\frac{1}{6}$
5	$\frac{2}{3}$	$\frac{1}{6}$	$-\frac{1}{\sqrt{3}}$	$\frac{1}{6}$
6	$\frac{1}{6}$	$\frac{2}{3}$	$-\frac{1}{\sqrt{3}}$	$\frac{1}{6}$

Supplementary Table 2: Gauss points used in calculation. Related to STAR methods

$$\frac{\partial \boldsymbol{x}}{\partial \boldsymbol{\xi}} = \left(\begin{bmatrix} Shape \\ Function \\ Derivatives \end{bmatrix} \boldsymbol{x} \right)^T = \left(\begin{bmatrix} \frac{\partial N_1}{\partial \xi} & \frac{\partial N_2}{\partial \xi} & ... & \frac{\partial N_6}{\partial \xi} \\ \frac{\partial N_1}{\partial \eta} & \frac{\partial N_2}{\partial \eta} & ... & \frac{\partial N_6}{\partial \eta} \\ \frac{\partial N_1}{\partial \zeta} & \frac{\partial N_2}{\partial \zeta} & ... & \frac{\partial N_6}{\partial \zeta} \end{bmatrix} \begin{bmatrix} x_1 & y_1 & z_1 \\ x_2 & y_2 & z_2 \\ . & . & . \\ . & . & . \\ . & . & . \\ x_6 & y_6 & z_6 \end{bmatrix} \right)^T \tag{32}$$

and similarly,

$$\frac{\partial \boldsymbol{X}}{\partial \boldsymbol{\xi}} = \left(\begin{bmatrix} Shape \\ Function \\ Derivatives \end{bmatrix} \boldsymbol{X} \right)^T \tag{33}$$

2.7.3 Calculation of nodal elastic forces

After obtaining $\boldsymbol{F^e}$ through the calculation in Section 2.7.2, the elemental Cauchy stress can be calculated via equation (9), through the right Cauch-Green strain tensor and the second Piola-Kirshoff stress tensor $\boldsymbol{S}^e$ in (10). Once the elemental elastic stresses are obtained, these can be mapped to the nodal forces through equation (34),

$$\boldsymbol{g^e_{elemental}} = \iiint \boldsymbol{B}^T \boldsymbol{\sigma^e} |\boldsymbol{F}| \left| \frac{\partial \boldsymbol{X}}{\partial \boldsymbol{\xi}} \right| d\xi d\eta d\zeta \tag{34}$$

Here, matrix $\boldsymbol{B} = [\boldsymbol{B}_1 \ldots \boldsymbol{B}_n]$ is the deformation matrix, where each matrix $\boldsymbol{B}_i$ is given by (Hughes, 2008):

$$\boldsymbol{B}_i = \begin{bmatrix} N_{i,x} & 0 & 0 \\ 0 & N_{i,y} & 0 \\ 0 & 0 & N_{i,z} \\ N_{i,y} & N_{i,x} & 0 \\ N_{i,y} & 0 & N_{i,x} \\ 0 & N_{i,z} & N_{i,x} \end{bmatrix} \tag{35}$$

and with $N_{i,x}$ being the short hand for $\partial N_i/\partial x$. Note that these derivatives are computed using the Jacobian $\partial \boldsymbol{x}/\partial \boldsymbol{\xi}$ in 32,

$$\nabla_x N_i = \left(\frac{\partial \boldsymbol{x}}{\partial \boldsymbol{\xi}} \right)^{-T} \nabla_\xi N_i$$

where $\nabla_x N_i$ is the nodal shape function derivative array as in $\nabla_x N_i = [N_{i,x}\ N_{i,y}\ N_{i,z}]^T$ and $\nabla_\xi N = [N_{i,\xi}\ N_{i,\zeta}\ N_{i,\zeta}]^T$.

2.7.4 Calculation of the stiffness matrix ($\mathbf{K}^{elast}$)

The stiffness matrix $\boldsymbol{K^{elast}}$, elastic part of the system Jacobian, is calculated in two parts, as given in the integral form in equation (11). The first part is carried out through a series of nested loops for the nodal contributions of node pair a & b. For any $\boldsymbol{K}_{ab,ik}$,

the ik component of the nodal contributions for the node pair ab of the on the element, the summation should be carried out over j,l & IJKL. The volume integration is included by the multiplication by determinants of the deformation gradient and reference shape position derivatives with respect to parametric coordinates.

$$[\mathbf{K}^e_{ab}]^1_{ik} = \iiint \sum_j \sum_l \sum_I \sum_J \sum_K \sum_L F^e_{iI} F^e_{jJ} F^e_{kK} F^e_{lL} \mathcal{C}_{IJKL} \nabla_x N^b_l \nabla_x N^a_j \, |\boldsymbol{F^g}| \left| \frac{\partial \boldsymbol{X}}{\partial \boldsymbol{\xi}} \right| d\xi d\eta d\zeta. \tag{36}$$

Here, $\nabla_x N^b$ is the nodal shape function derivative array as in $\nabla_x \boldsymbol{N}^b = [N_{b,\xi} \; N_{b,\zeta} \; N_{b,\nu}]^T$ and $\mathcal{C}$ is the Lagrangian elasticity tensor obtained from equation (12).

The second part of the integral is calculated from the $\nabla_x N^a$ and $\nabla_x N^b$ as defined above, the elemental stresses from equation (9) and the volume integration:

$$[\mathbf{K}^e_{ab}]^2_{ik} = \iiint \nabla_x N^{aT} \sigma^e \nabla_x N^b \, |\boldsymbol{F}| \left| \frac{\partial \boldsymbol{X}}{\partial \boldsymbol{\xi}} \right| d\xi d\eta d\zeta. \tag{37}$$

2.8 A pseudo algorithm of the simulation procedure

```
Initiate simulation mesh
    Set up system symmetry
    Set up tissue bounding box and relative element positions
    Set up tissue compartments (ECM, apical actomyosin layer)
    Assign tissue physical properties
    Set up boundary conditions
    Calculate shape function derivatives
    Calculate nodal masses and exposed external surfaces
    Induce mutant clones (if applicable)
Set time, t = 0.
While t < t^{final}:
    Reset all system forces
    Update elemental rotation matrices R_{er}.
    Update bounding box and relative element positions.
    Calculate F^g increments from experimental growth maps or other
    specified input (Section 2.4).
    Calculate ECM remodelling increments F^r (Section 2.5).
    Update total growth F^g of elements (Section 2.4).
    Update nodal exposed surfaces (Equation (14)).
    Detect packing nodes (Section 2.6.1).
    Update node adhesion (Section 2.6.2).
    Update elemental collapse to avoid flipping (Section 2.6.2).
    Solve for positions via implicit Newton-Raphson numerical integration:
       Initiate x_n^k = x_{n-1} for k=1.
       While the iteration displacements (δx) have not converged to zero:
          Reset matrices (g = 0 , K = I).
          Calculate nodal displacements dx(t) = x_n^k - x_{n-1}.
          Calculate elastic terms g^{elast}_{elemental} and K^{elast}_{elemental}:
             Interpolated at 6 Gauss points for each element:
                Calculate F (Section 2.7.2).
```

```
            Extract F^e (Equation (7)).
            Calculate σ (Equation (9)).
            Calculate g^elast_elemental (Section 2.7.3).
            Calculate K^elast_elemental (Section 2.7.4).
         Add each g^elast_elemental to system residual g.
         Add each K^elast_elemental to system Jacobian K.
      Calculate external viscous resistance terms:
         Calculate and add g^ext to system residual g (Section 2.3).
         Calculate and add K^ext to the system Jacobian K (Section 2.3).
      Calculate packing:
         Calculate and add packing forces to g (Section 2.6.1).
         Calculate and add packing related terms to K (Section 2.6.1 ).
      Update fixed degrees of freedom and node binding (Section 2.6.2).
      Solve for δx from g and K (Equation (6)).
      Update x_n^{k+1} = x_n^k + δx
      k = k + 1
      Check if system converged with norm(δx) < 1E-8
   t = t + Δt
```

3 Analysis of simulation results

3.1 Automated fold detection and construction of apical indentation maps

Fold initiation is automatically detected by the curvature of the facing regions on the apical surface. This is done by calculating the surface normal of all elements exposed on the apical surface. If the surface normals within the vicinity of each other face in opposite directions (dot product being negative), the elements are defined to be on a folding curve. All the elements lying in between the two elements are also included on the fold surface (Fig. S1G). The threshold distance for identifying curved regions is selected as 3 μm. The threshold is selected to ensure detection of fold initiation that is clearly visible when the morphology is visualised, but it does not assign fold initiation identity to elements at opposing sides of a possible curve peak. For the looser folds of the uniform growth rate simulations, a more generous threshold of 6 μm is used. In the apical indentation maps, these automatically identified indentations are marked on the tissue outline with each continuous indentation given in a single colour (for example Fig. 3Aii).

3.2 Simulation fold position scoring

The positions of the continuous folds that are detected and that reach the midline are aligned with the experimental fold positions (Fig, 1Cii), so that the minimum deviation is calculated. If the simulation produces less than 3 folds, each missing fold is counted as 100% deviation. The deviation score is calculated as sum of percentage deviation from each experimental fold at the tissue centre, maximum total deviation being 300% The score does not penalise for additional folds, such as those observed with uniform growth of tissues with explicit BM definition (Figure 5E). The deviation score also does not check the fold morphology, such as the high hinge folds with uniform growth rates that reach taller than the pouch region (Fig. 3Ai). Therefore each low deviation value should be examined against both additional folds and non-biological fold morphology.

Parameter	Description	Value / Range	Reference
Tissue viscoelasticity			
E_{cells}	Cellular layer Young's modulus	25 Pa	Simulated for range
E_{actin}	Actin rich apical layer Young's modulus	26 - 200 Pa	Simulated for range
η_{ext}	External viscous resistance coefficient	10 - 16000 $Pa\ s\ \mu m^{-1}$	Simulated for range
ν	Poisson ratio of the tissue	0.29 $Pa\ s\ \mu m^{-1}$	Schluck et al, 2013
Basement membrane			
h_{BM}	The thickness of basement membrane	$0.2\mu m$	Calculated from measurements in this study
$t_{1/2}$	Basement membrane remodelling half-life	1 - 16 hr	Simulated for range
E_{BM}	Basement membrane Young's modulus	400 - 3200 Pa	Simulated for range
Node-node interactions			
Δ^{pack}	Packing hard-wall potential cut-off distance	40 % of local average mesh side length	selected for numerical stability selected for numerical stability
d_0	Packing hard-wall potential sigmoid shift	60 % of Δ^{pack}	selected for numerical stability
s^{sat}	Packing hard-wall potential sigmoid saturation term	5	standard logistic function
$\Delta^{collapse}$	The distance threshold below which nodes of an element are collapsed	10 % of equivalent reference shape edge length	selected for numerical stability
$\Delta^{adhesion}$	The distance threshold below which two nodes adhere	Δ^{pack}	selected for numerical stability
Tissue dimensions	The width, length and height of the tissue	see Fig. 1	Measurements in this study
Growth rates	Spatio-temporal growth rates defined by the growth maps	see Fig. 4	Measurements in this study

Supplementary Table 3: Parameters of the simulations. Related to STAR methods
