# Supplementary material for: Planar Differential Growth Rates Initiate Precise Fold Positions in Complex Epithelia
Source: Dev Cell. 2019 Nov 4;51(3):299–312.e4. doi: 10.1016/j.devcel.2019.09.009 (PMC6838681; doi:10.1016/j.devcel.2019.09.009)
Supplement: Document S2. Article plus Supplemental Information [file mmc7.pdf]

Article

Developmental Cell

Planar Differential Growth Rates Initiate Precise Fold Positions in Complex Epithelia

Graphical Abstract

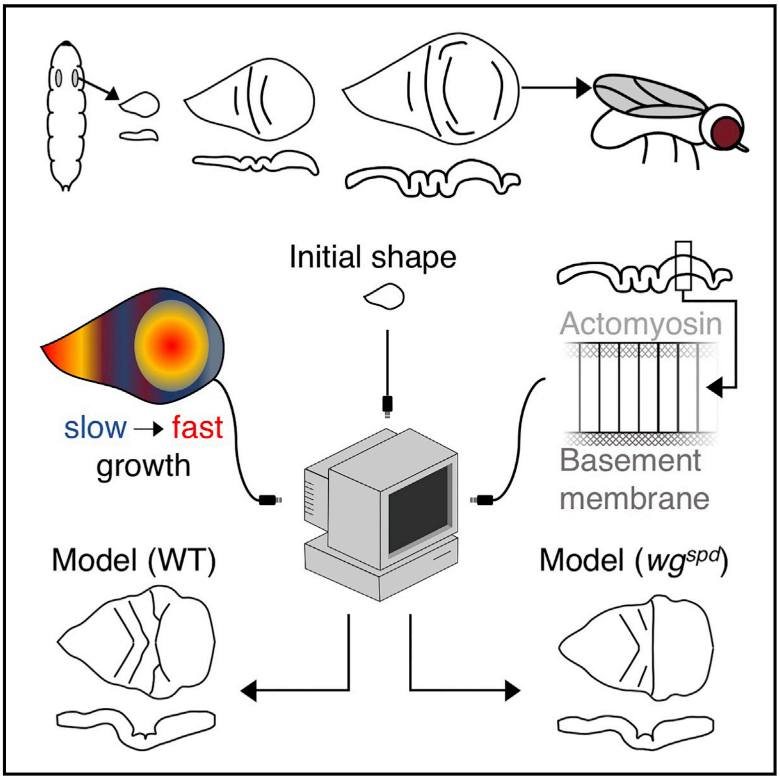

Authors

Melda Tozluoğlu, Maria Duda, Natalie J. Kirkland, Ricardo Barrientos, Jemima J. Burden, José J. Muñoz, Yanlan Mao

Correspondence

y.mao@ucl.ac.uk

In Brief

Like an origami crane forming from the folds of a paper, the precise positioning of multiple folds in epithelia is key for the final shape of a tissue. Tozluoğlu et al. present a computational model that explains how precise fold positioning can be predicted by heterogeneous growth in epithelia.

Highlights

- *Drosophila* wing discs grow with spatial and temporal heterogeneity
- This differential growth determines the positions of epithelial folds
- Constriction from the basement membrane is necessary for correct fold initiation
- Our computational model correctly predicts the shape of growth mutants

Tozluoğlu et al., 2019, Developmental Cell *51*, 299–312
November 4, 2019 © 2019 The Author(s). Published by Elsevier Inc.
https://doi.org/10.1016/j.devcel.2019.09.009

CellPress

Developmental Cell
Article

CellPress

Planar Differential Growth Rates Initiate Precise Fold Positions in Complex Epithelia

Melda Tozluoğlu,[1] Maria Duda,[1] Natalie J. Kirkland,[1] Ricardo Barrientos,[1] Jemima J. Burden,[1] José J. Muñoz,[2] and Yanlan Mao[1,3,4,5,*]
[1]MRC Laboratory for Molecular Cell Biology, University College London, London WC1E 6BT, UK
[2]Mathematical and Computational Modeling (LaCàN), Universitat Politècnica de Catalunya, Barcelona, Spain
[3]Institute for the Physics of Living Systems, University College London, London WC1E 6BT, UK
[4]College of Information and Control, Nanjing University of Information Science and Technology, Nanjing, Jiangsu 210044, China
[5]Lead Contact
*Correspondence: y.mao@ucl.ac.uk
https://doi.org/10.1016/j.devcel.2019.09.009

SUMMARY

Tissue folding is a fundamental process that shapes epithelia into complex 3D organs. The initial positioning of folds is the foundation for the emergence of correct tissue morphology. Mechanisms forming individual folds have been studied, but the precise positioning of folds in complex, multi-folded epithelia is less well-understood. We present a computational model of morphogenesis, encompassing local differential growth and tissue mechanics, to investigate tissue fold positioning. We use the *Drosophila* wing disc as our model system and show that there is spatial-temporal heterogeneity in its planar growth rates. This differential growth, especially at the early stages of development, is the main driver for fold positioning. Increased apical layer stiffness and confinement by the basement membrane drive fold formation but influence positioning to a lesser degree. The model successfully predicts the *in vivo* morphology of overgrowth clones and *wingless* mutants via perturbations solely on planar differential growth *in silico*.

INTRODUCTION

Epithelial folding is a fundamental morphological process that is encountered abundantly during the development of multiple organisms. It is used to sculpt organs from flat epithelial sheets into complex structures such as tubular, undulated, and branched tissues (Nelson, 2016). Folds may function as a means of compartmentalization, surface area increase to facilitate material exchange, or may emerge as a side effect of pathology, such as overgrowth in cancer (Gutzman et al., 2008; Hruban et al., 2000; Nelson, 2016).

Possibly the most extensively studied driver of folding is apical constriction via accumulation of non-muscle myosin II (Dawes-Hoang, et al., 2005; Granholm and Baker, 1970; Lecuit and Lenne, 2007; Lewis, 1947; Polyakov et al., 2014). Basal relaxation, lateral constriction (Štorgel et al., 2016; Sui et al., 2012, 2018; Wang et al., 2016; Wen et al., 2017) and cell shortening (Conte et al., 2012; Gutzman et al., 2008; Sherrard et al., 2010), constriction of the regions surrounding the prospective fold (Kondo and Hayashi, 2013; Monier et al., 2015; Röper, 2012), and constriction of supporting structures by other cells (Hughes et al., 2018), have all been demonstrated as potential folding mechanisms. Other force generation mechanisms, such as cell rounding in mitosis, adhesion shifts, or basal extrusions can also induce folds (Kondo and Hayashi, 2013, 2015; Monier et al., 2015; Wang et al., 2012). In all these scenarios, what defines the position of the prospective fold is a biochemical signaling mechanism responsible for selecting the cell population to actively generate the forces.

Beyond cellular forces, differential growth between tissue layers can induce patterns of buckling such as those of the gut (Savin et al., 2011), dental epithelium (Marin-Riera et al., 2018), brain (Tallinen et al., 2014), lungs (Kim et al., 2015), and the rippled edges of plant leaves (Dervaux and Ben Amar, 2011; Liang and Mahadevan, 2009; Marder et al., 2003). External structures, such as the extracellular matrix (ECM), can provide sufficient confinement to growing tissue to induce folding (Diaz-de-la-Loza et al., 2018). Uniform growth and constriction will induce folds in predictable patterns following the physical rules of buckling (Karzbrun et al., 2018; Pocivavsek et al., 2008; Shyer et al., 2013; Wang and Zhao, 2015). The patterns can then be refined by the overall shape of the tissue (Tallinen et al., 2016) or further local perturbations such as constriction by smooth muscles (Kim et al., 2015), adhesive forces, and ECM alterations (Sui et al., 2012).

While shaping a tissue, various mechanisms are likely to occur in parallel, such that once a fold is initiated in a selected position, a combination of modifications of the confinement and active force generation can help its progression. A key question is, how are the initial positions of the folds defined to achieve the precise tissue morphology (Nelson, 2016)?

As an emergent mechanical phenomenon, fold position selection is likely to depend on a combination of the forces accumulating in the growing tissue, the dynamics of surrounding structures, and the inherent properties of the tissue such as stiffness or shape prior to folding. None of these factors are trivial to investigate independently in an experimental system—how would one eliminate the influence of the shape of a tissue on its form? Therefore, the topology of folding morphogenesis is a problem particularly suitable for computational exploration.

This is an open access article under the CC BY license (http://creativecommons.org/licenses/by/4.0/).

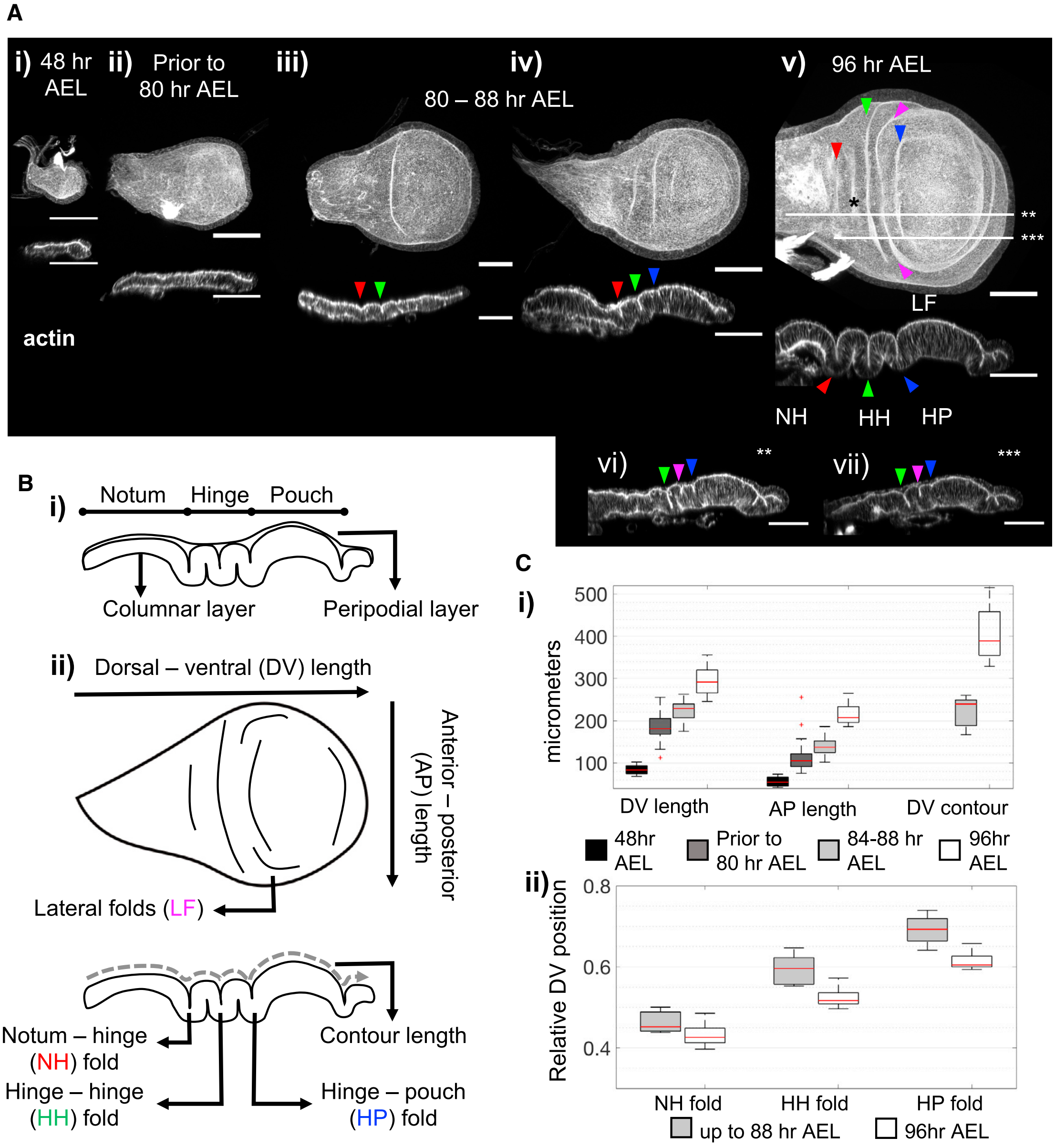

Figure 1. Characterization of Wing Imaginal Disc Morphology

(A) (i–v) The morphology changes between 48 and 96 h AEL. Maximum projection images, top and cross-section from DV axis midline views. Arrowheads point to HN, HH, HP, and LF in red, green, blue, and magenta, respectively. Scale bars are 50 μm. Due to the projection, basal folds are visible on the top view, example marked by black star on (v). (vi and vii) Lateral cross-sections along lines marked with white stars on (v).

(B) Schematic of the wing disc structure. (i) Domains are labeled, the thin peripodial layer is hardly visible on the experimental images. (ii) Top and cross-section with developmental axes and fold names labeled.

(C) (i) Wing disc size during fold formation, developmental age progresses from black to white, see STAR Methods for n. At 48 h AEL, the mean AP and DV lengths are 56 and 84 μm, respectively. Prior to 80 h AEL, 114 and 185 μm; at 88 h AEL, 128 and 222 μm. At 96 h AEL, 214 and 294 μm, the apical contour length on the DV axis is 402 μm. (ii) Fold positions normalized to DV length, error bars represent one standard deviation. Up to 88 h AEL, the positions are 0.48, 0.58, and 0.66 for NH, HH, and HP folds respectively. At 96 h AEL, they are 0.43, 0.52, and 0.61. 72–88 h AEL, NH fold n = 22, HH fold n = 29, HP fold n = 18. 96 h AEL, n = 14 for all folds. Boxes represent 25th and 75th percentiles, medians in red, whiskers extend the most extreme data points that are not outliers, outliers plotted with red plus.

Drosophila melanogaster is an established model system for studying morphogenesis. The wing imaginal disc of *Drosophila* forms three distinct folds, perpendicular to the dorsal-ventral axis. These major folds are highly reproducible in their number and positions, marking the boundaries between the notum, hinge, and pouch regions of the wing disc (Figure 1). There is evidence that basal relaxation, lateral constriction, and stiffness changes within the cell compartments play roles in the generation of the folds (Sui et al., 2012, 2018; Wang et al., 2016). However, what determines their positions and drives the

initiation of these folds is an open question. This makes the wing disc an ideal experimental system to investigate general mechanisms that control the position of folds in complex epithelia, a problem that has been under-investigated but critical in determining the final functional architecture of the tissue.

Here, we investigate the minimum set of requirements for initiating the correct topology of the complex multi-folded wing disc epithelia. Our search allows us to postulate planar differential growth as a mechanism for fold initiation. We measure the differential planar growth rates of wing imaginal disc development with high spatial resolution. Utilizing a computational approach, and from experimental measurements, we demonstrate that the differential growth in the plane of the tissue, especially in the early stages, under the compression of the ECM, drives the initiation of three folds from the apical surface. When challenged with mutants, our model can successfully recapitulate the morphology of overgrowth clones. We also predict that a reduction of early growth in the hinge region, prior to any folds being visible, can affect the number and position of folds that form later. We experimentally validate this prediction against a *wingless* mutant, which has reduced proliferation specifically in the hinge region (Neumann and Cohen, 1996). Our simulations show that the alterations of planar growth rates are sufficient to explain the observed fold perturbations in overgrowth and undergrowth mutants.

RESULTS

Characterization of Wing Imaginal Disc Fold Morphology

The wing imaginal disc is a monolayered epithelial sac. The peripodial layer, positioned as the top layer throughout this paper, is formed of squamous cells. The bottom layer is the columnar layer, which forms the folds by the end of third instar (Figures 1Aiv, 1Av, and 1B). The apical surfaces of both layers face each other toward the lumen, and the basal surfaces face outwards. Both apical and basal surfaces harbor ECM of different compositions (Pastor-Pareja and Xu, 2011; Ray et al., 2015).

We focus our attention on the columnar layer and characterize the two-day period from 48 h AEL, when the tissue is flat (Figure 1Ai), to 96 h AEL, when the columnar layer has formed three folds at the hinge region (Figures 1Av and 1B). From dorsal to ventral tips, these folds are termed notum-hinge (NH), hinge-hinge (HH), and hinge-pouch (HP) folds (Figure 1Bii). Between the HH and HP folds, the tissue forms additional lateral folds (LF) that do not reach the midline (Figures 1Avi, 1Avii, and 1Bii). There are multiple smaller folds at the ventral tip of the wing disc, where the tissue loops connect the columnar layer to the peripodial layer. These smaller folds are beyond the scope of the current work, as the largely unknown dynamics of the peripodial layer are not included in the current model.

In our analysis, we segment the development into three morphological stages (Figures 1A and S3A): (1) The early stage before initiation of folds, ending prior to approximately 80 h AEL. The tissue is relatively flat, and folds do not start forming (Figures 1Aii and 1Ci). (2) The intermediate stage where the folds are starting to initiate on the apical surface, with the possibility that the HH fold has fully formed, ending by 88 h AEL (Figures 1Aiii, 1Aiv, and 1Ci). (3) The stage where all folds are established, ending approximately 96 h AEL (Figures 1Av and 1Avi). The folds are formed at reproducible, precise positions as normalized to the tissue DV length (Figure 1Cii).

We match the initial state of our simulations to the tissue size and shape at 48 h AEL and start simulations using simple growth rates derived from the changes in dimensions of the wing disc as described above (Figure 1Ci). Step by step, we add in external confinement with apical ECM and BM, physical property heterogeneities, and fine growth patterns to characterize the requirements of fold initiation in the wing disc.

The Computational Model

For the purposes of identifying the mechanisms driving wing disc folding, we develop a finite element (FE) model (Bonet and Wood, 2008) of tissue morphogenesis. The goal of this model is to realistically capture the mechanical behavior of the tissue, then simulate its shape dynamics for different tissue physical properties and growth scenarios. The dynamics of the tissue emerge by balancing the elastic forces upon deformation and viscous resistances against the shape changes at each time step.

In our model, the tissue is defined as a non-homogeneous continuous material. It is composed of a set of elastic FEs in the shape of triangular prisms. These prism elements are independent of the cells in the tissue and can have sizes varying from subcellular to multicellular scales (Figures 2A–2C and S1A). These elements have fixed "reference shapes," which represent the initial shape of the element (Figure 2B, red wireframe). The growth is defined in the form of a deformation relative to the element's reference shape. The shape resulting from the combination of growth and the reference shape becomes the element's current grown or "preferred" shape (Figure 2B, blue wireframe). The total deformation of each element is calculated with respect to its reference shape at each time step. This total deformation is then decomposed into two components, the expected deformation due to growth of the element and the remaining elastic deformation (Rodriguez et al., 1994; Taber, 1995) (Figure 2B). Elements resist the elastic deformation with reactive forces (Figure 2C). The dynamics of morphogenesis then is governed by the forces depending on the viscoelastic properties and growth of the tissue. The relationship between the elastic deformations and resistance forces is modeled as a Neo-Hookean material, and a viscous resistance from the environment is applied on the exposed surfaces. In our approach, we extend the existing decomposition methodology to include oriented growth that follows the plane of the tissue (Figures 2D and S1B). In addition to active growth, the model allows for remodeling of tissue sub-compartments, such as the BM. In remodeling, the growth of an element is slowly updated such that the preferred shape slowly approaches to its current (deformed) shape, relaxing the tissue strains in the process.

The model starts with the preferred and current shapes of each element being equal to the element's reference shape. As the tissue grows in time, a mismatch between the preferred and current shape emerges, creating elastic deformations and generating consequent reactive forces. Balancing these elastic forces against deformation with the viscous resistance to movement at each time step, the new current shape of each element, therefore the whole tissue is obtained.

Further details of the modeling procedures, meshing (Shewchuk, 2005), boundary conditions (Muñoz and Jelenić, 2004),

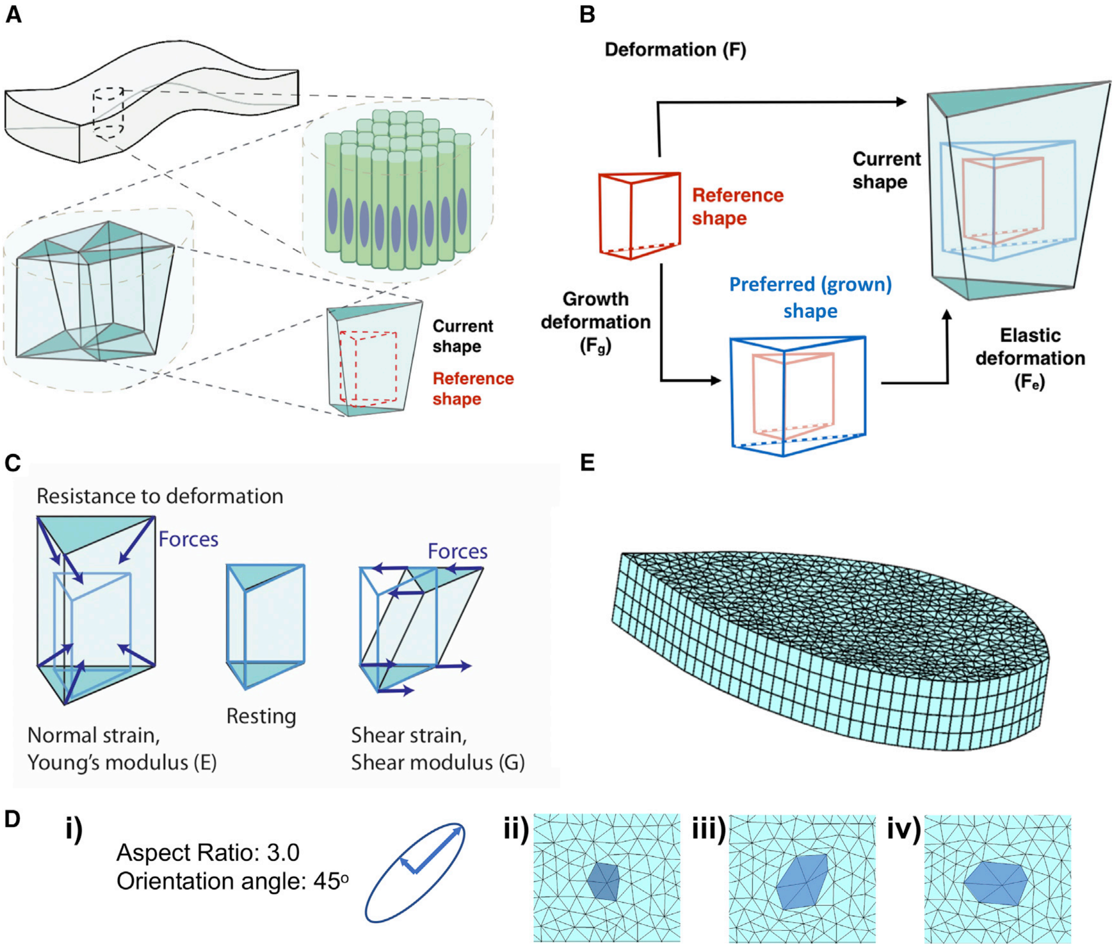

Figure 2. The Computational Model Design

(A) Schematic describing the finite elements.

(B) Schematic of growth methodology in the model, red wire plot, the reference shape of the element; blue wire plot, the preferred shape of the element.

(C) Elastic forces generated by elements upon deformation.

(D) Simulation of clone (dark blue) growth surrounded by non-growing tissue (i) the oriented growth input (ii) Initial state of a tissue fragment, (iii) after application of growth in (i). (iv) Tissue in (ii) after growing with the same rates at an orientation angle of zero degrees.

(E) Initial mesh of the simulations, at 48 h AEL. See also Figure S1.

linearization (Hughes, 2000), a pseudo code of the simulations and a table summarizing all utilized model parameters can be found in Methods S1, Methodology for the Computational Model.

Resistances from Apical-Basal Surface Confinement Are Essential for Folding

We start our simulations with uniform, constant growth rates that would bring the tissue AP and DV contour lengths from their sizes at 48 to 96 h AEL, in a 48-h time window (0.028 h^{-1} in AP and 0.033 h^{-1} in DV). Simulations with uniform growth on a tissue with minimal external resistance and homogenous physical properties do not form any folds (Figure 3A, top-left corner). This result reinforces that the tissue must have external factors driving compression.

During morphogenesis, both apical ECM and BM can exert resistances to tissue growth and movement and contribute to correct tissue architecture (Diaz-de-la-Loza et al., 2018; Harnezo et al., 2015). Initially, we represent this as a viscous external resistance on the apical and basal surfaces. As the viscous resistance is increased, the tissue starts forming ripples with compact, regular folds, initiating from the center and distributing toward the dorsal and ventral edges (Figure 3Ai, first column). To assess the fold initiation, we generate apical indentation maps (Figure 3Aii), then calculate the total deviation in fold positions as a percentage of the DV length, each missing fold deviation contributing 100% to the sum (Figure 3Aiii). Within the tested range with uniform tissue physical properties (Figures 3Ai–3Aiii, first columns), the tissue can form only two folds. The peaks of these folds are also higher than the apical surface of the pouch and notum regions, which is not the case for the wing disc.

Next, we consider physical property heterogeneities as a source of breaking symmetry. It is likely that the imaginal disc epithelium has heterogeneities, especially along its apical-basal axis. The dense actomyosin mesh on the apical surface, the actin

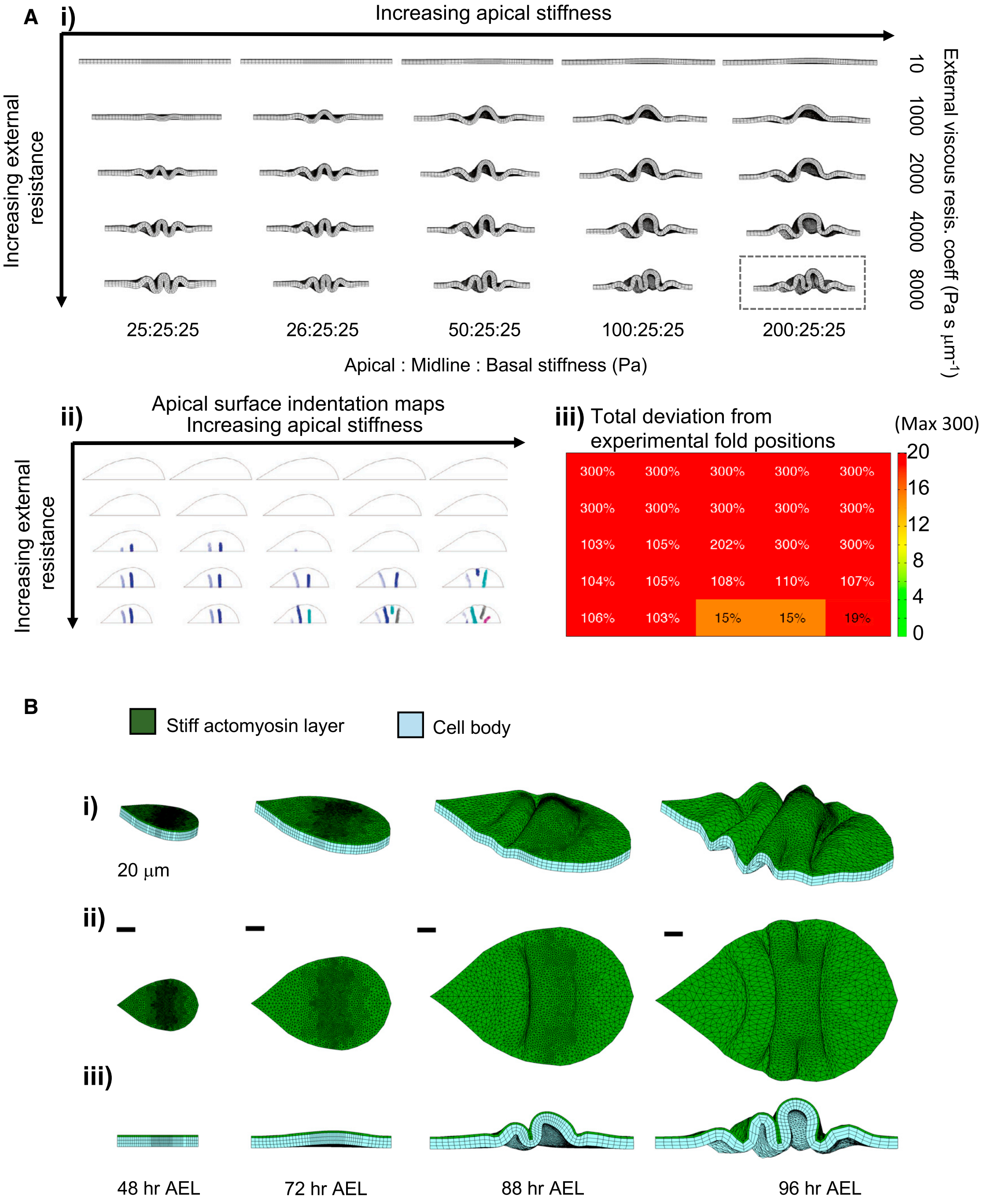

Figure 3. Relative Increase in Apical Stiffness and External Resistance to Tissue Growth Are Essential for Fold Formation

(A) (i) Simulations from 48 to 96 h AEL, with uniform planar growth rates. Images show the cross-section from the DV axis midline at 96 h AEL, ventral tip on the right. Columns, increasing external viscous resistance; rows, increasing apical stiffness relative to the rest of the tissue. Poisson ratio is taken to be 0.29 for all simulations (Schluck et al., 2013). (ii) Apical indentation maps. (iii) Fold position deviation scores. The grid organization is same in (i–iii).

(B) Simulation boxed in (Ai). (i) Orthogonal perspective view, (ii) top view, (iii) cross-section from the DV axis midline. Scale bars are 20 μm. See Video S1 and Figure S2.

of the basal surface, or the accumulation of the cell nuclei in the middle zone could all result in higher stiffness than the rest of the cell (Farhadifar et al., 2007; Meyer et al., 2011; Sui et al., 2018). To account for all these possibilities, we simulate wing disc growth with an increased stiffness on the apical surface (Figure 3A), on apical and basal surfaces (Figure S2A), or on the midline (Figure S2B). Of the tested cases, only stiffness increase on the apical surface can generate three folds; with

15%–19% deviation from the correct fold positions at tissue midline (Figures 3Ai–3Aiii bottom rows, B; Video S1). However, none of the cases generate folds similar to the experimental morphology (Figure 1Av).

With this analysis, we conclude that external resistance to growth is essential for buckling the tissue and that increased apical stiffness can induce correct number of folds. However, defining growth rates as uniform and the BM as a simple viscous resistance is not sufficient to induce folds in the correct positions and shapes. Therefore, we construct detailed maps of tissue growth at fine spatial and temporal resolutions to improve the implemented growth rates.

Wing Imaginal Disc Planar Growth Patterns Show Spatial and Temporal Heterogeneity

We experimentally measure the local growth rates via clonal analysis. By inducing sufficiently sparse single cell clones at different ages AEL, we quantify the local growth rates and generate spatial growth maps (Figure 4A). The extent of growth is defined by the number of nuclei in each clone; the orientation and aspect ratio of growth are defined by the ellipse fitted to the clone shape (Figure 4Aii). This analysis results in coupled growth rate heatmaps and orientation maps, in three-time windows as identified from the morphological quantifications (Figures 4B, S3A, and S3E; STAR Methods, Growth Rate Analysis).

The maps reveal that the wing disc harbor high spatial and temporal variability in its planar growth patterns. Specifically, notum and pouch regions have significantly higher growth than the hinge region at early stages (Figures 4B and S3E). Similar to the previous observations for the pouch region (Mao et al., 2013), the overall growth rates of the tissue are reduced as the tissue ages.

Next, we implement these measured growth rates in our simulations and model the 48 hours of development (48–96 h AEL). Here, the measured planar growth rates of three morphological age groups are applied sequentially in three equal (16 h) time windows (Figure S3Aiii), and growth is assumed to be constant through each window. With apical and basal confinement from viscous resistances, the experimental growth patterns result in folding patterns distinct from those of the uniform growth (Figures 4C and 4D; Video S2). Some indications of apical indentations emerge around the hinge region (Figure 4Dii, red arrows), and two lateral apical indentations that merge at tissue midline in later stages form (Figure 4Diii). None of the tested cases can initiate three distinct folds. This suggests that additional to the spatial-temporal variability in growth patterns, the BM should also be modeled in finer detail.

Characterization and Explicit Definition of the Basement Membrane of Wing Discs

To characterize the morphology of the BM structure, we acquire electron microscopy (EM) images of wing discs at pre-folding stages (72 h AEL) and at the end of third instar (120 h AEL) (Figures 5Ai and 5Aii) and quantify the thickness of the BM (Figure 5Aiii). The images reveal the BM is an approximately 0.1-μm thick uniform sheet in young discs. For older discs, the BM is a more complex structure with multiple layers and thickness in the range 0.4–0.6μm. Assuming a constant thickening rate of BM at the pouch center, the average thickness between 72 and 96 h AEL is then 0.2 μm. We thus define the BM in our model as a 0.2-μm thick elastic layer encapsulating the tissue (Figure 5B).

In the simulations, the BM grows with remodeling upon deformation. Each BM element grows in the orientation of its current deformation, at a rate set by the local remodeling half-life (Methods S1). This leads to the gradual relaxation of BM deformations, and an emergent, non-homogenous growth of the BM influenced by the growth of the cellular layer. For clarity, the remodeling is represented in a 2D schematic in Figure 5C. We then simulate the development of the wing disc with a series of BM stiffness, remodeling half-life, and apical viscous resistance coefficient parameters.

Differential Planar Growth Rates of the Tissue Constrained by an Elastic Basement Membrane Drives Precise Fold Initiation

Upon definition of the elastic BM, we initially investigate apical-basal tissue stiffness heterogeneity ranges (Figures S4A–S4C), using the experimentally measured growth rates (Figure 4B). As we increase the apical viscous resistance, the tissue starts forming buckles (Figures S4A–S4C). For the cases with increased apical stiffness and increased stiffness on both surfaces, three apical indentations emerge (Figures S4A–S4Ci). Of the two scenarios, increased apical stiffness initiates a more dome-like pouch, proportional hinge fold indentations, and lower percentage deviations from the experimental fold positions (Figures S4A–S4Cii), better mimicking the *in vivo* fold pattern.

Simulation snapshots for a setup with 100 Pa apical and 25-Pa cell body stiffness demonstrate the emerging indentations and their depth as the development progresses (Figures 5Di–5Dvi; Video S3). In the apical indentation maps, we can see the emergence of all three folds, the curved pouch border marked by the HP fold (gray, then cyan) and the emergence of the LF (cyan) (Figure 5Dvii). The folds are concentrated to the central region of the tissue, close to the experimental fold positions (Figure 5Dviii), with a total deviation of 4%. We predict the initiation of the folds requires the BM to be an order of magnitude stiffer than the cellular layer, which indeed has been inferred to be the case (Keller et al., 2018). As long as the BM is dramatically stiffer than the cellular layer, this phenotype can be produced with a large range of stiffness and remodeling half-life parameter sets for the BM, relative changes in one compensating for the other (Figure S5A). Simulating the same parameter set as Figure 5D with uniform growth reveals numerous symmetric ripples on the apical surface (Figure 5E). This signifies the importance of planar differential growth in the precise selection of the number and position of the folds.

Our simulation also initiates an ectopic buckle on the pouch, which is not observed in live tissue (Figure 5Dvii, red line, Fi). To decipher what may be driving the resistance of the pouch region to such buckling, we turn our attention to tissue thickness. Our analysis reveals, through the 48 h of our interest, the wing disc increases its thickness in a non-uniform manner (Figure 5Fii), with the pouch region becoming relatively thicker than the rest of the tissue. The emergent tissue thickness increase during the simulations brings the pouch height to 17.3μm, and notum height to 13.3μm at 84 h AEL, from the initial uniform height of 12.5μm at

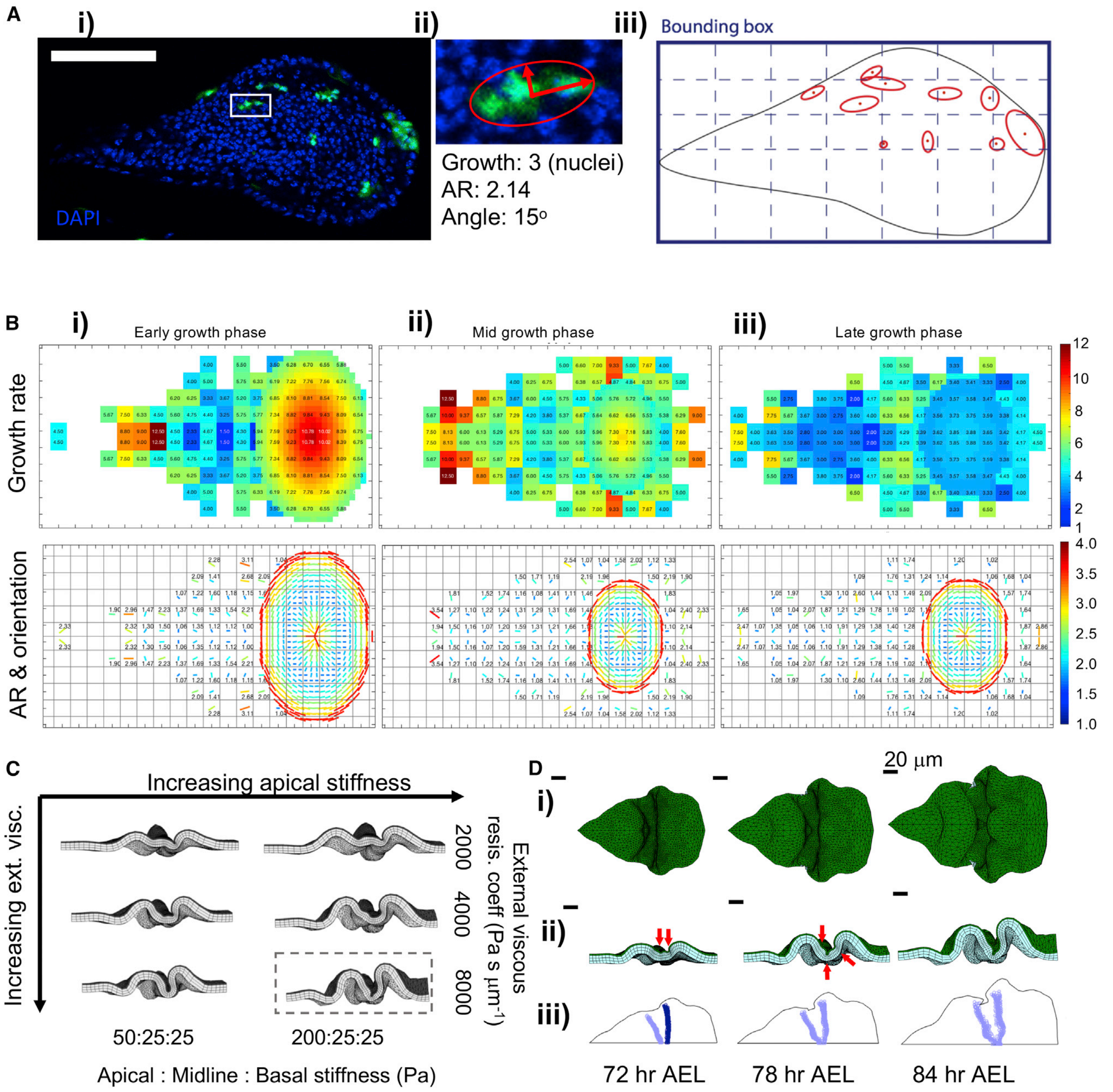

Figure 4. Wing Imaginal Disc Planar Growth Patterns Show Spatial and Temporal Heterogeneity

(A) (i) 72 h AEL wing disc with sparse single cell clones (green), scale bar 50 µm. (ii) Clone marked by the white box in (i) with fitted ellipse (red); AR, aspect ratio. (iii) Schematic showing fitted ellipses of all clones in (i), mapped on to a grid on the bounding box of the projected image.

(B) Top panels, growth maps. Bottom panels, growth orientation maps. The major axis of the average fitted ellipse are shown with lines. The length and color of the line represent the AR, orientation of the line represents growth orientation angle. (i) 48–80 h AEL, (ii) 56–88 h AEL, (iii) 72–96 h AEL.

(C) Simulations with growth maps in (B). Increasing apical stiffness on rows, increasing external viscous resistance coefficient in columns.

(D) Snapshots for the boxed simulation (C). (i) top, (ii) cross-section. Emerging indentations marked by red arrows. (iii) Apical indentation maps. See Video S2; Figure S3.

48 h AEL. This is well below the experimental observations (Figure 5Fii); therefore, the thickness increase should be an active growth input.

Implementing this growth in the tissue thickness prevents pouch surface buckling (Figures 5G and S4D, and Video S4), with a total fold position deviation of 10% (Figure S5A). Our simulations predict that within the mechanical context where the hinge folds are initiated, the relative thickness of the pouch region protects it from further buckling that would be otherwise induced by the compression.

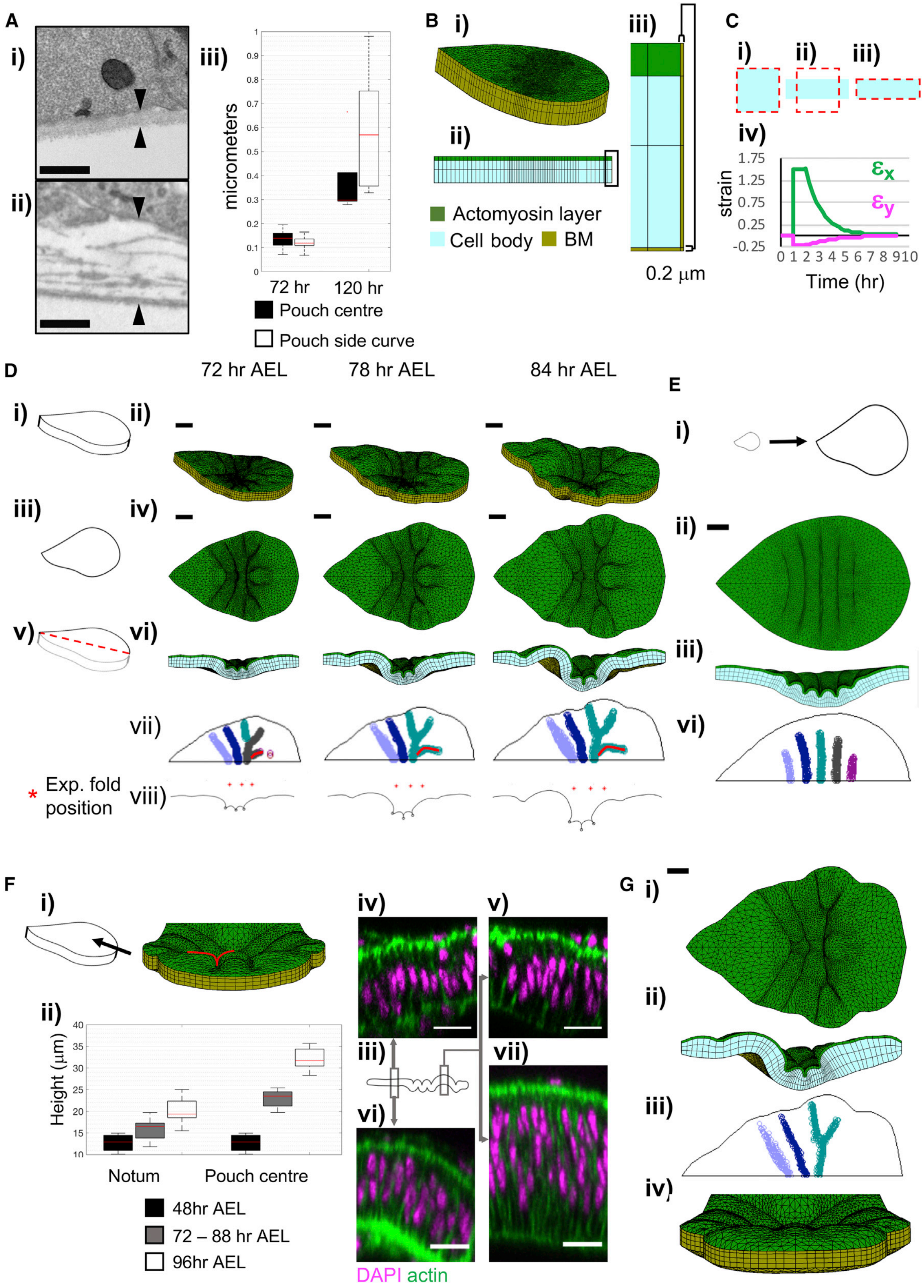

Figure 5. An Explicitly Defined BM and Planar Differential Growth Rates Enable Emergence of *In Vivo* Mimetic Fold Morphology

(A) EM images of the wing disc BM below the pouch, (i) 72 h AEL, and (ii) 120 h AEL. Arrowheads mark the thickness measurement. Scale bars are 0.5 μm. See Figure S5C. (iii) BM thickness below the pouch. Box represents 25th and 75th percentiles, median in red, whiskers extend the most extreme data points.

(legend continued on next page)

Emergent Three-Fold Pattern Is Robust against Volume Variability

Throughout the analysis, number of nuclei is used as a surrogate for tissue growth. To identify the potential impact of cell volume variations, we generate volume variability maps from the clone analysis and obtain volume scaled growth maps (Figures S5Fi–S5Fiii). Simulations with the volume scaled growth maps can capture the main pattern of the three folds, but additional ectopic buckling along the anterior-posterior axis between HH and HP folds is seen (Figure S5Fiv). The core pattern of fold initiation with differential growth is robust against observed variations in cell volume, but approximating the tissue growth as a combination of the number of nuclei and observed volume does not improve model predictions. The observed volume of a clone is a combination of the cell's preferred volume and its deformations, as opposed to the nuclei count and the definition of growth in the model. This disparity, combined with the challenges of accurate volume measurements in the complex pseudostratified wing disc epithelia, negates potential additional information a true volume measurement could provide. As such, in line with our parsimonious approach throughout the paper, we continue to approximate growth with number of nuclei and assume constant cell volume throughout development in further model tests.

Early Growth Pattern Is Sufficient for Correct Fold Initiation

Our results so far demonstrate the importance of planar differential growth in defining fold positions. The simulations reveal the fold initiation starts by the end of the early growth phase (Figure S4E). It has been shown that wing discs dissected immediately prior to fold initiation are still able to initiate folds *ex vivo* upon blocking cell division (Sui et al., 2018). With these, we investigate if the early growth rates are sufficient to initiate the folds upon significant perturbation to growth thereafter. We run simulations where the early growth rates in Figure 4Bi are applied for the first 16 h (48 to 64 h AEL) as in the control case, and then the growth is continued in the simplest form, with the uniform rates. The early growth rates followed by uniform growth are sufficient for the emergence of *in vivo* mimicking fold morphology (Figure S6Ai). Similarly, WT growth up to 64 h AEL, followed by non-oriented growth at the experimental growth magnitudes, does not alter the emerging fold morphology (Figure S6Aii). Additionally reducing the growth rate to 50% of the experimentally measured rates results in a minimal discontinuity in the fold pattern, but the structure is preserved (Figure S6Aiii). When the simulations are run with control growth rates, but the accumulated forces are relaxed prior to the emergence of the folds, the emerging morphology is perturbed. The final topology has loose fold formation, and the pouch curve shrinks compared to the control simulations (Figure S6B). These results suggest that the early growth rates measured prior to initiation of the folds, and the corresponding force accumulation, are critical for correct tissue morphology.

The Simulations Successfully Predict the Disrupted Fold Morphology upon Perturbations of Planar Differential Growth Patterns in Overgrowth Clones and *Wingless* Mutants

Next, we challenge our simulations with perturbations in tissue growth, both overgrowth and undergrowth. Given the sufficiency of early growth rates for patterning the tissue, we must select perturbations that are effective prior to the formation of any folds. First, we experimentally induce overgrowth clones via increasing insulin signaling at 48 h AEL, by expressing a constitutively active form of the insulin receptor. The clones induce apical and basal bulging of the tissue, with ectopic fold initiation at the clone boundaries (Figure 6A). We simulate overgrowth clones with a range of growth rates, and we can capture the same deformations (Figure 6B). The apical bulging is visible from 200% growth increase, ectopic folding can be observed at 300% and above (Figure S6C), which is in line with overgrowth magnitudes observed for other perturbations in the wing disc (Genevet et al., 2009).

Next, we test undergrowth. Again, this mutant must have an effect before the folds form. The expression of *wingless* (*wg*) localizes in two concentric rings encapsulating the pouch region from early third instar, before the folds start initiating. *spade^{flag}* (*spd^{fg}*) mutations in *wg* lead to loss of *wg* expression at the inner ring, coinciding with the hinge. Consequently, the mutant has

(B) (i and ii) Initial simulation mesh with the BM, (iii) close up of the boxed area in (ii).

(C) Remodeling exemplified with single 2D element, (i–iii) blue quadrilateral is the current shape; dashed red lines depict the preferred shape with remodeling imposed on the reference shape. (i) Initial state, (ii) immediately after deformation, corners fixed on the x direction. (iii) Same element after area conserving remodeling. (iv) Green strains against time. Element deformed at t = 1 h, remodeling activated at t = 2 h, with half-life of 1 h.

(D) Snapshots from simulation with explicit BM definition. Apical stiffness is 100 Pa, cell body stiffness is 25 Pa, BM stiffness is 1,600 Pa with renewal half-life of 8 h, apical viscous resistance coefficient is 16,000 Pa s μm^{-1}, and basal is 10 Pa s μm^{-1} (Video S3). (i–vi) Schematics representing the views and the simulation snapshots. (i and ii) orthogonal perspective, (iii and iv) top, (v and vi) cross-section from the DV axis midline. All snapshots at the same scale, bars are 20 μm. (vii) Apical indentation maps. Ectopic pouch fold is marked with red line. (viii) the apical surface contour of the DV midline cross-section with computational fold positions (black circles). Experimental fold positions for 72–88 h AEL (Figure 1Ci) are in red stars.

(E) Simulation results with uniform growth on the tissue plane, simulation parameters same as (D), growth rate same as Figure 3A. (i) Schematic to scale, representing uniform growth from 48 to 96 h AEL. (ii–iv) top and cross-section views and the apical indentation map at 84 h AEL. Scale bar, 20 μm.

(F) Close up view of tissue pouch from ventral tip showing the ectopic folding (red line). (i) Schematic representing the view and simulation snapshot at 84 h AEL. (ii) Experimental tissue heights. Box represents 25th and 75th percentiles, median in red, whiskers extend the most extreme data points, n = 4 for 48 h AEL, n = 14 for 72–88 h AEL, and n = 17 for 96 h AEL. Measured at 48 h AEL, 72–88 h AEL, and 96 h AEL, the notum thickness is 12.5 ± 2.17, 15.81 ± 2.46, and 20.19 ± 2.95 μm, meanwhile the pouch center thickness is 12.5 ± 2.17, 22.96 ± 1.86, and 32.2 ± 2.26 μm, (mean and one standard deviation). (iii) Schematic representing the positions images. (iv and v) the nuclei positions of the notum (iv) and pouch (v) prior to fold formation. (vi and vii) Notum (vi) and pouch (vii) at 96 h AEL. Scale bars, 10 μm.

(G) Simulation with tissue height increase at 84 h AEL, all remaining parameters are same as (D). Bar 20 μm. (i) top view, (ii) cross-section view, (iii) apical indentation map, (vi) view from ventral tip, as a comparison to (Fi). See Video S4 and Figures S4 and S5.

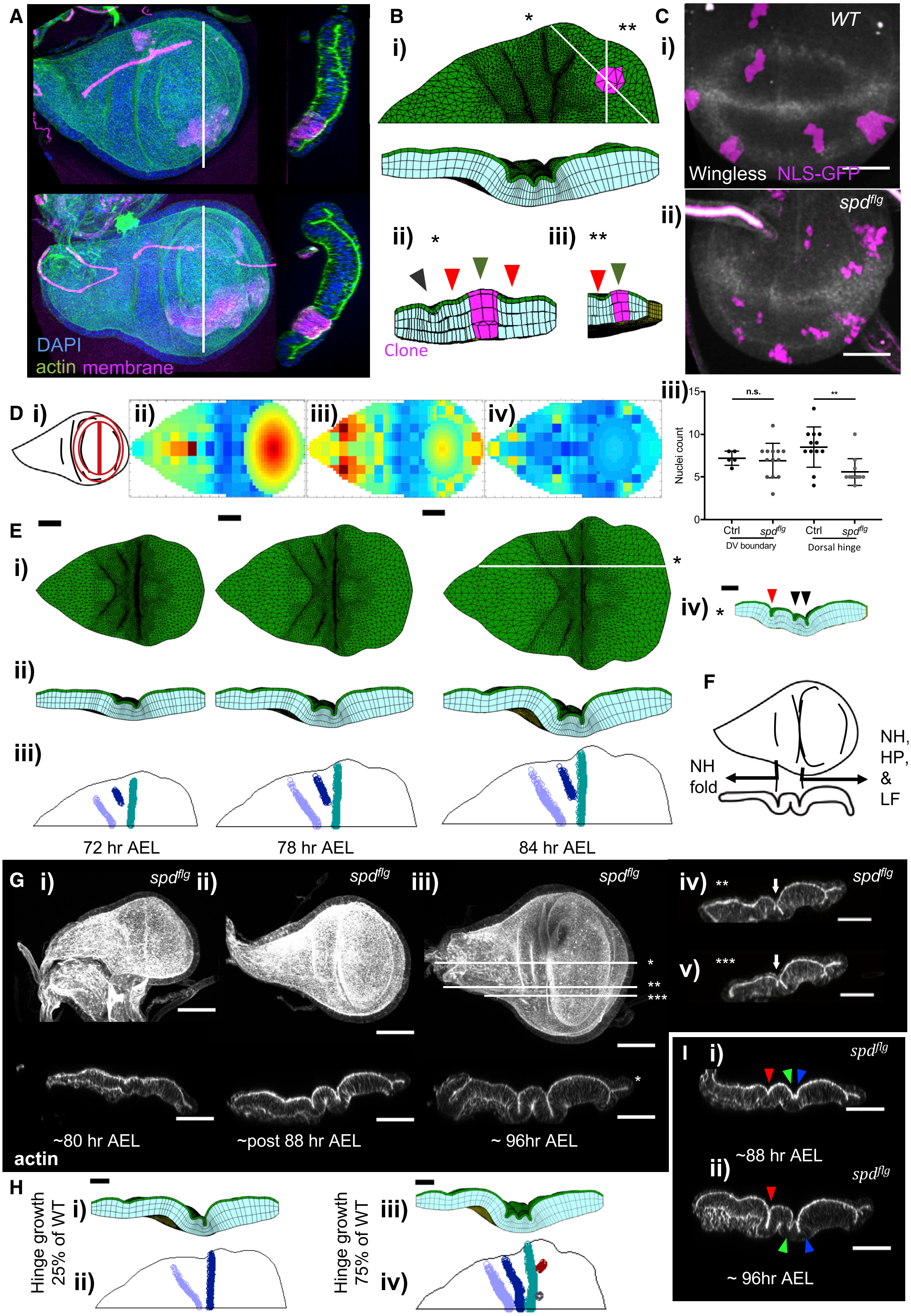

(legend on next page)

reduced growth in the hinge, and reduced size in the corresponding region of the adult wing (Neumann and Cohen, 1996; Rodríguez Dd et al., 2002). First, we carry out a clone growth rate analysis to demonstrate growth is reduced in *spd*^{fg} mutant during 2nd instar stage (Figure 6C). We quantify the clone size in the region corresponding to the dorsal side of the inner ring that is lost in *spd*^{fg}. This quantification reveals a 35% reduction in clone size for the mutant with respect to the wild type (Figure 6Ciii). *Wg* expression in the pouch DV boundary is preserved in the mutant. As an internal control, we quantify the clone size in this *wg* expression band and show that there is no reduction in growth at the DV boundary (Figure 6Ciii). These clearly demonstrate that the *spd*^{fg} mutant reduces growth specifically in the wing disc hinge during early wing disc development (48–72 h AEL), prior to fold initiation.

Next, to predict the wing disc phenotype of this mutation, we run simulations where hinge growth is reduced in the range 75% to 25% of WT. At 50% growth reduction (Figure 6D), the simulated mutant loses the three-fold morphology and displays only two folds at the midline of the disc (Figure 6E). On the lateral side, a third fold starts emerging and collapses on the pouch side fold before reaching the midline (Figure 6Eiv). It follows that the *spd*^{fg} mutant should be able to form the NH fold, while HH and PH folds and the LF will collapse into a single fold at the tissue midline (Video S5) (Figure 6F). To test our prediction, we examine *spd*^{fg} mutant wing discs (Figure 6G). The morphology of mutant wing discs at sequential developmental stages clearly demonstrates the emergence of a two-folded morphology (Figures 6Gi–iii) instead of three. Further, investigating the lateral cross-sections of the tissue at 96 h AEL shows that a third fold initiates at the lateral regions, yet collapses with the pouch side fold before reaching the tissue midline (Figures 6Giv and 6Gv), matching with the model predictions (Figure 6Div; Video S5). Simulations also reveal a dose dependent perturbation of the fold structure. At growth rates as low as 25% of WT, the third fold does not emerge from the lateral sides (Figures 6Hi and 6Hii), whereas at 75%, three folds form at the midline, albeit being more compact than the WT (Figures 6Hiii and 6Hiv). Then going back to the experiments, we could identify cases where small peaks emerged at the groove of the pouch side fold at tissue midline (Figure 6I). This further supports the hypothesis that the HH fold collapses with the HP fold and the LF in the *spd*^{fg} mutant. Depending on the level of growth perturbation, the loss of the HH fold can be observed gradually, again matching with the predictions of the simulations.

DISCUSSION

Here, we present a computational model of tissue growth and morphogenesis, incorporating spatial and temporal heterogeneity in growth rates and orientations, BM mechanics and remodeling, and physical property heterogeneities within different layers of the tissue. Coupled with the stiff apical surface and BM mechanics, we demonstrate that the planar differential growth rates are key in defining the positions of epithelial folds of the wing disc. Upon identifying early growth phases as sufficient for the initiation of folds, we make predictions on the emergent morphology upon perturbation of early growth rates. By changing only the planar differential growth rates *in silico*, we successfully predict overgrowth clone and *wingless* mutant morphologies *in vivo*. With our computational analysis, we propose a mechanism whereby planar differential growth rates define epithelial fold initiation positions.

In the BM mechanics, we identify, a large range of parameters will suffice as long as the BM is elastic and sufficiently stiff (8 times the apical stiffness and above). This observation could explain the robust folding of the wing disc against the high spatial and temporal variability in BM structure (Figure 5A). Our simulations also show that some form of viscous resistance on the apical surface is necessary. The apical ECM has

Figure 6. Simulations Successfully Predict the Disrupted Fold Morphology of Overgrowth Clones and *Wingless* Mutants upon Perturbations of Planar Differential Growth Patterns

(A) Wing discs with insulin overgrowth clones (magenta, membrane labeled by CD8-mCherry) - 96 h AEL. Maximum projection of the top view, cross-sections through the whites lines.

(B) Simulation snapshots for overgrowth clone at 75 h AEL, initial clone diameter is 4 μm (in line with wing disc cell diameters), growth is 3 times the WT without orientation, (see also Figure S6C). All physical properties same as Figure 5D. (i) Top panel: top view with the clone highlighted, bottom panel: cross-section from the DV axis midline. (ii and iii) the cross-section from the white lines in (i), (ii) is (*), and (iii) is (**). Green arrowheads point to apical bulging, red point to ectopic folds at the borders of the clone, gray points to the LF.

(C) *spd*^{flg} mutant growth quantification with clone analysis, clones in magenta. Scale bar, 25 μm. (i) WT, (ii) *spd*^{flg} mutant. (iii) Quantification of clone size in the proximity of the wg expression band at the pouch DV boundary (n = 5 for WT, n = 13 for *spd*^{flg}), and the wingless expression ring, the dorsal side of pouch only (n = 12 for WT, n = 12 for *spd*^{flg}). Means and one standard deviations marked, ** depicts significant difference with p-value < 0.01 in a two tailed t test, n.s. marks not significant with p-value > 0.05. See also Figure S6D.

(D) (i) Schematic marking the pattern of wingless expression in WT. (ii–iv) the reduction of growth in the *spd*^{flg} defined in between the experimental positions of HN fold and center of the pouch, excluding the pouch itself. Growth maps for 50% growth reduction as sampled from the extended maps (see Figure S4) through the simulation. (i) 48–64 h AEL, (ii) 64–80 h AEL, and (iii) 80–96 h AEL. All represented as corresponding growth increase in 24 h. Color coding scale same as Figure 4B.

(E) Simulation with 50% reduced hinge growth. (i and ii) Simulation snapshots from (i) top and (ii) cross-section. (iii) Apical indentation maps. All simulation parameters except for the growth rates are same as Figure 5D. (iv) Lateral cross-section from the white line marked on (i) 84 h AEL, red arrowhead marks the NH fold, black arrowheads mark the laterally initiating folds, reminiscent of HH, HP folds, or the LF. Scale bars, 20 μm.

(F) Schematic displaying the collapse of HH and HP folds and the LF.

(G) the morphology of the mutant wing discs (i–iii) during the emergence of the folds. Scale bars, 50 μm. (iv and v) Cross-sections from the lateral side, demonstrating the emergence (white arrow) of lateral folds that collapse with the pouch side fold before midline. Of the white lines marked on (iii), (iv) is (**) and (v) is (***).

(H) Simulation results for reduced hinge growth at (i and ii) 25% and (iii and iv) 75% of WT. All remaining simulation parameters same as Figure 5D. Panels, color coding, and scale bars are same as (E).

(I) *spd*^{flg} mutant phenotypes with emergence of a residual small peak within the grove of the ventral side fold of the mutant, highlighting onefold is lost as a result of HH, and HP folds merging. NH, HH, and HP folds are marked in red, green, and blue arrowheads, respectively. See also Figure S6.

a different composition (Ray et al., 2015) and likely independent dynamics from the BM (Keller et al., 2018). The composition of the lumen, and any possible tethering interactions with the peripodial layer (Gibson and Schubiger, 2000), would resist apical surface movement with fluid-like viscous mechanics. Currently, we do not have any means of acquiring this viscous resistance of the apical ECM, yet our modeling approach signifies its importance.

While our model is able to successfully generate the three-fold pattern, the timing of fold initiation in our simulations deviate somewhat from the experimental observations (65 h AEL in simulations compared to 76–80 h AEL (Sui et al., 2018) *in vivo*, Figure S4E). Temporal dynamics of tissue and BM physical properties could lead to the offset in our fold initiation timing. With its complex structural variability (Figure 5A), it is not possible to assume monotonically increasing or decreasing BM stiffness, without extensive further studies. The stiffness of the tissue itself is most likely altered through the same period, given that the residual tension of the tissue has been shown to reduce over time (Rauskolb et al., 2014). The fact that we can observe fold initiation in the simulations prior to the emergence of the folds in the experiments indicates the accumulation of cell mass due to planar differential growth is sufficient for fold initiation, yet the tissue may not have reached the enabling physical state before 76 h AEL.

At later stages of the simulations, the successfully initiated hinge folds do not progress into fully established folds (Figure S5B). Our simulations suggest that once the folds are initiated with planar differential growth rates, additional mechanisms should be activated to progress these indentations into folds. Indeed, cell shortening, alteration of the microtubule and actin networks, modification of both interaction with the BM through integrins and the BM structure itself through MMPs, have all been reported as necessary requirements for progression of wing disc folding, each fold requiring particular subsets of modifications (Shen et al., 2008; Sui et al., 2012, 2018). These mechanisms and the mechanics of the accumulated cell mass due to differential growth can act together in a sequence of events that are temporally difficult to separate, acting concurrently or consecutively. Our findings set the scene for further theoretical and experimental investigation on the feedback mechanisms between the morphology of the apical surface and the signaling pathways regulating local growth, cell shape, and BM interactions. In our model, we impose the growth patterns from experimental observation. Further analysis is needed in order to determine the coupling between this growth distribution and other genetic or chemical factors.

In conclusion, our results suggest planar differential growth as a mechanism for determining tissue fold positions, independent of active force generation. With wider implications, we suggest that the growth patterns giving tissues their final size can also regulate their architecture. We show that forces may not always result in instantaneous morphology changes, but can result in delayed morphogenesis. Stresses may accumulate early during development, even without any obvious changes in tissue morphology, but these may be critical for the precise sculpting of the tissue later in development.

STAR★METHODS

Detailed methods are provided in the online version of this paper and include the following:

- KEY RESOURCES TABLE
- LEAD CONTACT AND MATERIALS AVAILABILITY
- EXPERIMENTAL MODEL AND SUBJECT DETAILS
  - *Drosophila* melanogaster
- METHOD DETAILS
  - Clone Generation
  - Immunostaining and Imaging
  - Electron Microscopy
  - The Model Definition
- QUANTIFICATION AND STATISTICAL ANALYSIS
  - Tissue Dimension Measurements
  - Growth Rate Analysis
  - Clonal Volume Measurement
- DATA AND CODE AVAILABILITY

SUPPLEMENTAL INFORMATION

Supplemental Information can be found online at https://doi.org/10.1016/j.devcel.2019.09.009.

ACKNOWLEDGMENTS

We thank all the authors and Erik Sahai, Robert Tetley, and Alejandra Guzman Herrera for revision of this manuscript. M.T. is funded by a Sir Henry Wellcome Fellowship (103095). M.D. is funded by a Marie Skłodowska-Curie Horizon 2020 Individual Fellowship (MRTGS). N.J.K. is funded by a MRC PhD studentship. R.B. is funded by CoMPLEX doctoral training program. J.J.M. is funded by Spanish Ministry of Economy and Competitiveness (DPI2016-74929-R) and Generalitat de Catalunya (2017 SGR 1278). Y.M. is funded by a MRC fellowship MR/L009056/1, a UCL Excellence Fellowship, a NSFC International Young Scientist fellowship 31650110472, a Lister Institute Research Prize Fellowship, and EMBO Young Investigator Programme. This work was also supported by MRC funding to the MRC LMCB University Unit at UCL, award code MC_U12266B.

AUTHOR CONTRIBUTIONS

M.T., J.J.M., and Y.M. conceptualized and designed the work. M.T. created the modeling software, carried out computational experiments, and analyzed the data. M.D. carried out tissue staging, growth rate, and tissue size experiments and analyzed the data. N.J.K. carried out tissue staging, wingless mutant, and tissue height experiments and analyzed the data. R.B. and J.J.B. carried out the EM experiments. All authors contributed to writing the manuscript.

DECLARATION OF INTERESTS

The authors declare no competing interests.

Received: February 6, 2019
Revised: July 10, 2019
Accepted: September 11, 2019
Published: October 10, 2019

REFERENCES

Bonet, J., and Wood, R.D. (2008). Nonlinear Continuum Mechanics for Finite Element Analysis (Cambridge University Press).

Conte, V., Ulrich, F., Baum, B., Muñoz, J., Veldhuis, J., Brodland, W., and Miodownik, M. (2012). A biomechanical analysis of ventral furrow formation in the Drosophila melanogaster embryo. PLoS One *7*, e34473.

Dawes-Hoang, R.E., Parmar, K.M., Christiansen, A.E., Phelps, C.B., Brand, A.H., and Wieschaus, E.F. (2005). Folded gastrulation, cell shape change and the control of myosin localization. Development *132*, 4165–4178.

Dervaux, J., and Ben Amar, M. (2011). Buckling condensation in constrained growth. J. Mech. Phys. Solids *59*, 538–560.

Diaz-de-la-Loza, M.-D.-C.D., Ray, R.P., Ganguly, P.S., Alt, S., Davis, J.R., Hoppe, A., Tapon, N., Salbreux, G., and Thompson, B.J. (2018). Apical and basal matrix remodeling control epithelial morphogenesis. Dev. Cell *46*, 23–39.

Farhadifar, R., Röper, J.C., Aigouy, B., Eaton, S., and Jülicher, F. (2007). The influence of cell mechanics, cell-cell interactions, and proliferation on epithelial packing. Curr. Biol. *17*, 2095–2104.

Gaul, U., Mardon, G., and Rubin, G.M. (1992). A putative Ras GTPase activating protein acts as a negative regulator of signaling by the Sevenless receptor tyrosine kinase. Cell *68*, 1007–1019.

Genevet, A., Polesello, C., Blight, K., Robertson, F., Collinson, L.M., Pichaud, F., and Tapon, N. (2009). The Hippo pathway regulates apical-domain size independently of its growth-control function. J. Cell Sci. *122*, 2360–2370.

Gibson, M.C., and Schubiger, G. (2000). Peripodial cells regulate proliferation and patterning of Drosophila imaginal discs. Cell *103*, 343–350.

Granholm, N.H., and Baker, J.R. (1970). Cytoplasmic microtubules and the mechanism of avian gastrulation. Dev. Biol. *23*, 563–584.

Gutzman, J.H., Graeden, E.G., Lowery, L.A., Holley, H.S., and Sive, H. (2008). Formation of the zebrafish midbrain-hindbrain boundary constriction requires laminin-dependent basal constriction. Mech. Dev. *125*, 974–983.

Hannezo, E., Dong, B., Recho, P., Joanny, J.-F.F., and Hayashi, S. (2015). Cortical instability drives periodic supracellular actin pattern formation in epithelial tubes. Proc. Natl. Acad. Sci. USA *112*, 8620–8625.

Hruban, R.H., Goggins, M., Parsons, J., and Kern, S.E. (2000). Progression model for pancreatic cancer. Clin. Cancer Res. *6*, 2969–2972.

Hughes, T.J.R. (2000). The Finite Element Method: Linear Static and Dynamic Finite Element Analysis (Mineola, NY: Dover Publications).

Hughes, A.J., Miyazaki, H., Coyle, M.C., Zhang, J., Laurie, M.T., Chu, D., Vavrušová, Z., Schneider, R.A., Klein, O.D., and Gartner, Z.J. (2018). Engineered tissue folding by mechanical compaction of the mesenchyme. Dev. Cell *44*, 165–178.

Karzbrun, E., Kshirsagar, A., Cohen, S.R., Hanna, J.H., and Reiner, O. (2018). Human brain organoids on a chip reveal the physics of folding. Nat. Phys. *14*, 515–522.

Keller, A., Lanfranconi, F., and Aegerter, C.M. (2018). The influence of geometry on the elastic properties of the Drosophila wing disc. Phys. Stat. Mech. Appl. *510*, 208–218.

Kim, H.Y.Y., Pang, M.-F.F., Varner, V.D., Kojima, L., Miller, E., Radisky, D.C., and Nelson, C.M. (2015). Localized smooth muscle differentiation is essential for epithelial bifurcation during branching morphogenesis of the mammalian lung. Dev. Cell *34*, 719–726.

Kondo, T., and Hayashi, S. (2013). Mitotic cell rounding accelerates epithelial invagination. Nature *494*, 125–129.

Kondo, T., and Hayashi, S. (2015). Mechanisms of cell height changes that mediate epithelial invagination. Dev. Growth Differ. *57*, 313–323.

Lecuit, T., and Lenne, P.F. (2007). Cell surface mechanics and the control of cell shape, tissue patterns and morphogenesis. Nat. Rev. Mol. Cell Biol. *8*, 633–644.

Lewis, W.H. (1947). Mechanics of invagination. Anat. Rec. *97*, 139–156.

Liang, H., and Mahadevan, L. (2009). The shape of a long leaf. Proc. Natl. Acad. Sci. USA *106*, 22049–22054.

Mao, Y., Tournier, A.L., Hoppe, A., Kester, L., Thompson, B.J., and Tapon, N. (2013). Differential proliferation rates generate patterns of mechanical tension that orient tissue growth. EMBO J. *32*, 2790–2803.

Marder, M., Sharon, E., Smith, S., and Roman, B. (2003). Theory of edges of leaves. Europhys. Lett. *62*, 498–504.

Marin-Riera, M., Moustakas-Verho, J., Savriama, Y., Jernvall, J., and Salazar-Ciudad, I. (2018). Differential tissue growth and cell adhesion alone drive early tooth morphogenesis: an ex vivo and in silico study. PLoS Comput. Biol. *14*, e1005981.

Meyer, E.J., Ikmi, A., and Gibson, M.C. (2011). Interkinetic nuclear migration is a broadly conserved feature of cell division in pseudostratified epithelia. Curr. Biol. *21*, 485–491.

Monier, B., Gettings, M., Gay, G., Mangeat, T., Schott, S., Guarner, A., and Suzanne, M. (2015). Apico-basal forces exerted by apoptotic cells drive epithelium folding. Nature *518*, 245–248.

Muñoz, J.J., and Jelenić, G. (2004). Sliding contact conditions using the master-slave approach with application on geometrically non-linear beams. Int. J. Solids Struct. *41*, 6963–6992.

Nelson, C.M. (2016). On buckling morphogenesis. J. Biomech. Eng. *138*, 021005.

Neufeld, N.P., de la Cruz, A.F., Johnston, L.A., and Edgar, B.A. (1998). Coordination of growth and cell division in the Drosophila wing. Cell *93*, 1183–1193.

Neumann, C.J., and Cohen, S.M. (1996). Distinct mitogenic and cell fate specification functions of wingless in different regions of the wing. Development *122*, 1781–1789.

Pastor-Pareja, J.C., and Xu, T. (2011). Shaping cells and organs in Drosophila by opposing roles of fat body-secreted collagen IV and perlecan. Dev. Cell *21*, 245–256.

Pocivavsek, L., Dellsy, R., Kern, A., Johnson, S., Lin, B., Lee, K.Y.C., and Cerda, E. (2008). Stress and fold localization in thin elastic membranes. Science *320*, 912–916.

Polyakov, O., He, B., Swan, M., Shaevitz, J.W., Kaschube, M., and Wieschaus, E. (2014). Passive mechanical forces control cell-shape change during Drosophila ventral furrow formation. Biophys. J. *107*, 998–1010.

Rauskolb, C., Sun, S., Sun, G., Pan, Y., and Irvine, K.D. (2014). Cytoskeletal tension inhibits Hippo signaling through an Ajuba-Warts complex. Cell *158*, 143–156.

Ray, R.P., Matamoro-Vidal, A., Ribeiro, P.S., Tapon, N., Houle, D., Salazar-Ciudad, I., and Thompson, B.J. (2015). Patterned anchorage to the apical extracellular matrix defines tissue shape in the developing appendages of Drosophila. Dev. Cell *34*, 310–322.

Rodríguez Dd, Ddel A., Terriente, J., Galindo, M.I., Couso, J.P., and Díaz-Benjumea, F.J. (2002). Different mechanisms initiate and maintain wingless expression in the Drosophila wing hinge. Development *129*, 3995–4004.

Rodriguez, E.K., Hoger, A., and McCulloch, A.D. (1994). Stress-dependent finite growth in soft elastic tissues. J. Biomech. *27*, 455–467.

Röper, K. (2012). Anisotropy of crumbs and aPKC drives myosin cable assembly during tube formation. Dev. Cell *23*, 939–953.

Savin, T., Kurpios, N.A., Shyer, A.E., Florescu, P., Liang, H., Mahadevan, L., and Tabin, C.J. (2011). On the growth and form of the gut. Nature *476*, 57–62.

Schluck, T., Nienhaus, U., Aegerter-Wilmsen, T., and Aegerter, C.M. (2013). Mechanical control of organ size in the development of the Drosophila Wing disc. PLoS One *8*, e76171.

Schneider, C.A., Rasband, W.S., and Eliceiri, K.W. (2012). NIH Image to ImageJ: 25 years of image analysis. Nat. Methods *9*, 671–675.

Shen, J., Dorner, C., Bahlo, A., and Pflugfelder, G.O. (2008). optomotor-blind suppresses instability at the A/P compartment boundary of the Drosophila wing. Mech. Dev. *125*, 233–246.

Sherrard, K., Robin, F., Lemaire, P., and Munro, E. (2010). Sequential activation of apical and basolateral contractility drives ascidian endoderm invagination. Curr. Biol. *20*, 1499–1510.

Shewchuk, J.R. (2005). Triangle: engineering a 2D quality mesh generator and Delaunay triangulator, Lect. Notes Comput. Sci.. In Applied Computational Geometry Toward Geometric Engineering, *1148*, M.C. Lin and D. Manocha, eds. (Springer), pp. 203–222

Shyer, A.E., Tallinen, T., Nerurkar, N.L., Wei, Z., Gil, E.S., Kaplan, D.L., Tabin, C.J., and Mahadevan, L. (2013). Villification: how the gut gets its villi. Science *342*, 212–218.

Štorgel, N., Krajnc, M., Mrak, P., Štrus, J., and Ziherl, P. (2016). Quantitative morphology of epithelial folds. Biophys. J. *110*, 269–277.

Sui, L., Alt, S., Weigert, M., Dye, N., Eaton, S., Jug, F., Myers, E.W., Jülicher, F., Salbreux, G., and Dahmann, C. (2018). Differential lateral and basal tension drive folding of Drosophila wing discs through two distinct mechanisms. Nat. Commun. *9*, 4620.

Sui, L., Pflugfelder, G.O., and Shen, J. (2012). The Dorsocross T-box transcription factors promote tissue morphogenesis in the Drosophila wing imaginal disc. Development *139*, 2773–2782.

Taber, L.A. (1995). Biomechanics of growth, remodeling, and morphogenesis. Appl. Mech. Rev. *48*, 487–545.

Tallinen, T., Chung, J.Y., Biggins, J.S., and Mahadevan, L. (2014). Gyrification from constrained cortical expansion. Proc. Natl. Acad. Sci. USA *111*, 12667–12672.

Tallinen, T., Chung, J.Y., Rousseau, F., Girard, N., Lefèvre, J., and Mahadevan, L. (2016). On the growth and form of cortical convolutions. Nat. Phys. *12*, 588–593.

Wang, D., Li, L., Lu, J., Liu, S., and Shen, J. (2016). Complementary expression of optomotor-blind and the Iroquois complex promotes fold formation to separate wing notum and hinge territories. Dev. Biol. *416*, 225–234.

Wang, Q., and Zhao, X. (2015). A three-dimensional phase diagram of growth-induced surface instabilities. Sci. Rep. *5*, 8887.

Wang, Y.C., Khan, Z., Kaschube, M., and Wieschaus, E.F. (2012). Differential positioning of adherens junctions is associated with initiation of epithelial folding. Nature *484*, 390–393.

Wen, F.L., Wang, Y.C., and Shibata, T. (2017). Epithelial folding driven by apical or basal-lateral modulation: geometric features, mechanical inference, and boundary effects. Biophys. J. *112*, 2683–2695.

STAR★METHODS

KEY RESOURCES TABLE

REAGENT or RESOURCE	SOURCE	IDENTIFIER
Antibodies		
Wingless MIgG1	DSHB	4D4; RRID: AB_528512
Donkey anti-mouse RRX	Jackson Immuno Research	715-295-151; RRID: AB_2340832
Chemicals, Peptides, and Recombinant Proteins		
DAPI	Sigma-Aldrich	D8417; RRID: AB_2307445
Phalloidin Alexa-647	Life Technologies	A22287
Phalloidin Alexa-555	Life Technologies	A34055
Hoechst	Sigma-Aldrich	B2261
Fluoromount G Slide mounting medium	Southern Biotech	0100-01
Bovine Serum Albumin	Sigma	A7030
Triton X-100	Sigma	T8787
16% w/v formaldehyde (Prediluted with PBS to 4% for fixation protocols)	TAAB Laboratories	F017/3
EM grade formaldehyde 36%	TAAB Laboratories Equipment Ltd	F003
EM grade glutaraldehyde 25%	TAAB Laboratories Equipment Ltd	G011
Osmium Tetroxide 2%	TAAB Laboratories Equipment Ltd	O005
Potassium ferricyanide	TAAB Laboratories Equipment Ltd	P018
Dodecenyl Succinic Anhydride	TAAB Laboratories Equipment Ltd	D027
2,4,6- Tri(Dimethylaminomethyl)	TAAB Laboratories Equipment Ltd	D032
Methyl Nadic Anhydride	TAAB Laboratories Equipment Ltd	M011
TAAB 812 resin	TAAB Laboratories Equipment Ltd	T023
Tannic Acid	TAAB Laboratories Equipment Ltd	T046
Lead Nitrate	TAAB Laboratories Equipment Ltd	L005
Potassium Ferricyanide	Sigma-Aldrich	P8131
Sodium citrate	Sigma-Aldrich	S4641
Experimental Models: Organisms/Strains		
w;; actin-FRT-CD2-FRT-Gal4, UAS-GFP^{NLS}	Neufeld et al., 1998	N/A
hsFLP	Bloomington *Drosophila* Stock Centre	BDSC8862
Yw	Bloomington *Drosophila* Stock Centre	
wingless$^{spd\text{-}fg}$	Bloomington *Drosophila* Stock Centre	BDSC1005
ywhsFLP; wg$^{spd\text{-}fg}$; S-T	Y.Mao	N/A
hsFLP; UAS-InR^{A1325D}/CyO; MKRS/TM6B	N. Tapon	N/A
w; actin-FRT-y-FRT-Gal4-UAS-CD8-mCherry/ CyO; arm-GFP/TM6	Y.Mao	N/A
Software and Algorithms		
Microsoft Excel 16	Microsoft	N/A
GraphPad Prism 7	GraphPad Software	N/A
Matlab 2016	Mathworks	N/A
ImageJ 1.51w	(Schneider et al., 2012) https://imagej.nih.gov/ij/docs/install/osx.html	N/A
Triangle - A two-dimensional quality mesh generator and Delaunay triangulator	(Shewchuk, 2005) https://www.cs.cmu.edu/~quake/triangle.html	N/A
Tissue morphogenesis FE simulation software	Bespoke model code developed in C++ for this study.	N/A

LEAD CONTACT AND MATERIALS AVAILABILITY

Further information and requests for resources and reagents should be directed to and will be fulfilled by the Lead Contact, Yanlan Mao (y.mao@ucl.ac.uk). All unique and/or stable reagents and the custom code generated for the manuscript are available from the Lead Contact upon request.

EXPERIMENTAL MODEL AND SUBJECT DETAILS

Drosophila melanogaster

Fly stocks were raised in non-crowded conditions on standard cornmeal molasses fly food medium at 25°C, unless otherwise indicated. Briefly, the fly food consisted of, per 1L, 10g agar, 15g sucrose, 33g glucose, 35g years, 15g maize meal, 10g wheat germ, 30g treacle, 7.22g soya flour, 1g nipagin, 5ml propionic acid. Male and female larvae were dissected at a range of developmental stages from 48hr AEL to 120hr AEL for experiments.

Strains used are listed in Key Resources Table, and include: For clone generation, tissue shape, and morphology measurements: hsFLP;; (BDSC8862) and w;; Act<CD2<GAL4, UAS-GFP (Neufeld et al., 1998). For wildtype height measurements: yellow white (yw;;) (BDSC). For *wingless* (*wg*) mutant analysis: ; *wg* $^{spd\text{-}fg}$; (BDSC1005), *wg* clones *yw*hsFLP; *wg*$^{spd\text{-}fg}$; S-T. For overgrowth clones with perturbation of the insulin pathway: hsFLP; UAS-InR^{A1325D}/CyO; MKRS/TM6B and w; Act<y<Gal4, UAS-CD8-mCherry/CyO; arm-GFP/TM6.

METHOD DETAILS

Clone Generation

To generate heat shock flip-out GFP clones of the correct density for growth rate analysis, the following regimes were used: for growth rates at 48–72h, 56-80h and 64–88h, heat shock was performed at 48h, 54hr or 64hr AEL respectively, for 12-20 min and dissected 24h later. For growth rates at 72–96 h, heat shock was performed at 72h for 10 min and wing discs dissected 24h later. All heat shocks were carried out at 37°C. To investigate spontaneous expression of GFP, we carried out non-heat shock controls (n=22 wing discs). Spontaneous GFP expression was indeed observed in larval epidermis, within the trachea and myoepithelial cells in the proximity of the wing disc. No such spontaneous expression is observed upon inspection of the wing disc columnar epithelium. For induction of insulin overgrowth clones marked by CD8-mCherry, heat shock was performed at 48hr AEL for 10 min at 37°C, followed by a 48hr growth before dissection. For the induction of flip-out GFP clones for growth rate analysis of the *wingless* mutant, the heat shock was performed at 48hr AEL for 10 min at 37°C and grown for 24 hrs before dissection.

Immunostaining and Imaging

Larval wing imaginal discs were dissected and stained as per the procedure described in (Gaul et al., 1992). In brief, wing discs were dissected at the appropriate age in ice cold PBS for up to 15 min and fixed in 4% formaldehyde in PBS, at room temperature, for 30 min.

For *wingless* mutants, fixed discs were repeatedly washed within a 40 min period in 0.3% PBT, followed by repeated washes with 0.5% BSA, 0.3% PBT for a further 40 min. Primary antibody, mouse anti-Wingless was prepared in 0.5% BSA, 0.3% PBT at 1:100 concentration and incubated overnight at 4°C. Washes were repeated as prior to primary antibody incubation. Secondary antibody, goat anti-mouse RRX (Jackson ImmunoResearch) (1:500), Alexa fluor 647-Phalloidin (Cell Signalling and Life Technologies) (1:20) and Hoechst (Sigma-Aldrich) (1:500) were prepared in 0.5% BSA, 0.3% PBT and incubated for 1 h at room temperature. Wing discs were washed repeatedly for 1 h in 0.3% PBT, prior to rinsing in PBS.

For *yw* larva used for height measurements and flip-out clone larva used for growth rate analysis, dissected and fixed wing discs were washed in 0.3% PBT repetitively for 20 min, then immediately incubated with Alexa fluor 647-Phalloidin (Cell Signalling and Life Technologies) (1:20) and Hoechst (Sigma-Aldrich) (1:500) in 0.3% PBT for 15 min at room temperature. Wing discs were washed repetitively for 30-40 min, and then rinsed with PBS.

Fixed and stained wing discs were mounted in fluoromount G Slide mounting medium (Southern Biotech) for imaging.

Wing discs were imaged on a Leica SP5 and SP8 inverted confocal microscope with a 40X oil objective at 1-2X zoom, 0.341 μm depth resolution and 512 by 512 or 1024 by 1024 pixel resolution.

Electron Microscopy

Wing discs were fixed in 2% formaldehyde/ 1.5% glutaraldehyde in PBS for 30 min prior to being flat, sandwich-embedded in 2.8% low melting point agarose dissolved in PBS. Once set, asymmetric cubes of agarose were cut out containing the wing discs, and they were secondarily fixed for 1 h in 1% osmium tetroxide/1.5% potassium ferricyanide at 4°C. Further fixation and contrast enhancement was achieved with, 1% tannic acid for 45 min. Samples were then dehydrated in increasing concentrations of ethanol solutions and embedded in Epon resin (TAAB 812). The 70nm ultrathin resin sections were cut with a diamond knife (Diatome) using an ultramicrotome (UC7; Leica) and sections were collected on formvar-coated slot grids and stained with lead citrate. Discs were imaged using a 120kV transmission electron microscope (Tecnai T12; FEI) equipped with a ccd camera (Morada; Olympus SIS).

The Model Definition

A detailed definition of the computational model, simulation results analysis methods and a table providing the parameter definitions together with the values used in the simulations are provided in the Methods S1, Methodology of the Computational Model.

QUANTIFICATION AND STATISTICAL ANALYSIS

Tissue Dimension Measurements

The tissue size is measured from maximum projection images. DV length is defined as the longest axis from ventral tip of the pouch to the dorsal tip of the notum. AP length is measured to be the longest axis of the tissue perpendicular to the measured DV axis. The number of discs measured for each stage are: 4 discs at 48hr AEL; 32 discs for early stages with no fold initiation; 38 discs for DV and 32 discs for AP for middle stages with some fold initiation; 22 discs for DV contour length, 31 discs for DV length and 17 discs for AP length for 96 h AEL discs. Fold positions are measured at the longest axis in tissue midline, corresponding to the axis of DV length measurement. Each fold position is normalized to DV length, dorsal tip being 0 and ventral tip 1. The NH fold position is averaged from 19 wing discs, HH fold from 26 and HP fold from 16 discs.

Growth Rate Analysis

The clone position, aspect ratio, and orientation were calculated with automated segmentation and ellipse fitting, number of nuclei in each clone was counted manually, all using ImageJ (Schneider et al., 2012). To convert the growth information of each clone to spatio-temporal maps of tissue growth, we go through age classification of the wing disc, alignment of wing morphology to average, binning the clone positions on a 2D projection of the tissue, and averaging data points, followed by overlaying the pouch growth rates from the literature (Mao et al., 2013), to generate the growth maps of Figure 4B. Next, the data points on the growth maps are intra- or extra-polated to cover the empty spaces of the map grid, to allow for the maps to be smoothly read during the simulations (Figures S3D and S3E).

For each clone, the manually counted number of nuclei determine the growth rate, the fitted ellipse determines the growth orientation and aspect ratio (Figure 4Aii). The centre of the fitted ellipse defines the position of the measured growth as normalised to the tissue bounding box. Assuming symmetry in the anterior-posterior axis, all single clone measurements are binned on one half of the bounding box (Figure 4Aiii).

The wing discs are divided into three age groups depending on their morphology. The first indication of folds on wing discs occurs between 72-80 h AEL (Sui et al., 2018). Therefore the wing discs with no visible fold initiation, except for minor actin accumulation on the fold region, corresponding to up to 80hr AEL age (Figure S3Ai) are classified into the “early phase”. The wing discs with at least one, mostly two to three initiated folds, but not reached to fully folded morphology, corresponding to the range 80-88hr AEL age (Figures S3Aii–S3Aiv) are classified into mid-phase and finally, wing discs with all three folds formed, corresponding to 96 h AEL age are classified into “late phase”. The age definitions are not clear-cut at all times, therefore we will refer to disc growth periods as early, mid, and late phase throughout the manuscript, referring to the above morphological characterisation (Figure S3A).

Upon division of the data points into age groups, where the wing disc has any markers for initiation of the first (HH) fold, the HH fold position of the individual disc is aligned to the average HH fold position, and the position of the clones updated (Figure S3B). This alignment step is not applicable to wing-discs of the earliest growth phase, where no folds are visible. Next, all the clones for a selected time point are binned on a 20 by 10 grid, according to their normalised planar positions within the bounding box of the tissue (Figure 4Aiii). Any grid bin with less than two data points is treated as empty. The growth data from the clones in each bin are averaged, Gaussian average is applied to number of nuclei, and the orientation angles, the orientation of the long axis of the fitted ellipse, are defined to be within $\pi/2$ degrees of each other before taking a Gaussian average. The aspect ratios are averaged with geometric average.

The growth rates of the pouch region of the wing disc have previously been characterised in high detail by Mao et al. (2013). We overlay these measurements onto the generated growth and orientation maps by defining the position of the HP fold as the dorsal tip of the pouch and using the pouch sizes measured in this study (Figure S3C). Finally, with the assumed anterior-posterior symmetry, the resulting growth map is reflected to the remaining half, and our final spatial-temporal growth profiles to be utilised in our simulations are obtained (Figures 4B, S3Aiii, and S3E).

The simulation requires a complete map, without gaps, such that each element can read its growth rate at each time step. Therefore, we fill the empty points of the grid by interpolating the existing measurements. Starting from the centre of the grid and moving out radially (Figure S3Di), once an empty grid point is detected, all the populated points within its eight immediate neighbors are averaged to fill the grid point (Figure S3Dii). The order of filling is of significance, as once a point is filled with averaging the neighbors, it will be counted as a populated point in following iterations. This allows us to fill the grid points at all regions, and we obtain the maps in Figure S3E. Of note, the corner points are not necessarily sampled in the simulation, as the emergent simulation tissue shape is similar to that of the experiments, nevertheless, the map should cover a slightly larger area then the immediate experimental boundary to ensure continuity. For an example of the region sampled throughout the simulations, see Figure 6D. While reading the growth rates from these maps, each element of the simulation takes its centre point normalised to tissue bounding box, reads the closest four corner values from the growth and orientation maps, and interpolates the actual growth rate/orientation to apply depending on its distance from each of the four corners.

Growth in apical-basal axis is calculated from measurement of tissue thickness (Figures 5Fii–5Fvii and S5D). The growth rate is directly calculated form the height increase, yet one complexity here became the pseudostratification of the tissue. As the tissue grows, the nuclei become pseudostratified, first in the pouch, followed by the notum (Figures 5Fiii–5Fvii and S5D). Coinciding with the relative pouch thickness increase, the pseudostratification is visible in the pouch region as early as the initiation of the HH fold as an apical indentation (Figure 5Fv), while notum nuclei are still organized in a single layer (Figure 5Fiv). As the development progresses, the pseudostratification can be seen everywhere, the extent being significantly higher in the pouch (Figures 5Fvi and 5vii). The measurements demonstrate tissue thickness can increase without pseudostratification (Figure 5Fiv), indicating addition of material to tissue height independent of cell division, i.e., the nuclei count from which we derive our planar growth rates. On the other hand, the pouch region of the tissue increases in height faster, to a greater extent, and pseudostratification is more predominant in this region (Figures 5Fiii and 5Fvi), which should influence our definition of planar growth. To account for this difference, we allowed for tissue height increase to the level of the notum thickening, without altering the planar growth rates, reflecting the cell height increase independent of nuclei stratification. The additional height increase observed for the pouch region, the difference between the notum and pouch height increases, is reduced from the planar growth rates, "using up" the increase in nuclei numbers.

Clonal Volume Measurement

We quantify the volume variability of the cells through an analysis analogous to growth rate analysis. The total volume of each clone is approximated by the maximum projected clone area and the height the clone spans in the z-stack. Then combining this data with the number of nuclei in the clone, we obtain the average cell volume in each clone. Normalising the measured cell volume to the average of the sample set, and following the same spatial and temporal classification of clones as in the growth rate analysis, we generate the volume variability maps (Figure S5F, top panels). Then we scale our growth maps with these volume maps, and obtained the volume scaled growth maps (Figure S5F, bottom panels).

Prism 7 was used for statistical analysis (Figure 6C). Two-tailed t test was used with the exact n values used for each of the experiments. The following statistical significance cut off was applied:

* $p < 0.05$,
** $p < 0.01$,
***$p < 0.001$,
****$p < 0.0001$.

DATA AND CODE AVAILABILITY

The authors declare that the data supporting the findings of this study and custom code generated for the manuscript are available from the corresponding author, Yanlan Mao (y.mao@ucl.ac.uk), upon reasonable request.

Developmental Cell, Volume *51*

Supplemental Information

Planar Differential Growth Rates Initiate Precise Fold Positions in Complex Epithelia

Melda Tozluoǧlu, Maria Duda, Natalie J. Kirkland, Ricardo Barrientos, Jemima J. Burden, José J. Muñoz, and Yanlan Mao

Index

Supplementary Figure Legends
- Supplementary Figure 1: related to Figure 2
- Supplementary Figure 2: related to Figure 3
- Supplementary Figure 3: related to Figure 4
- Supplementary Figure 4: related to Figure 5
- Supplementary Figure 5: related to Figure 5
- Supplementary Figure 6: related to Figure 6

Method S1: Methodology for the computational model, related to STAR Methods

Supplementary Figures

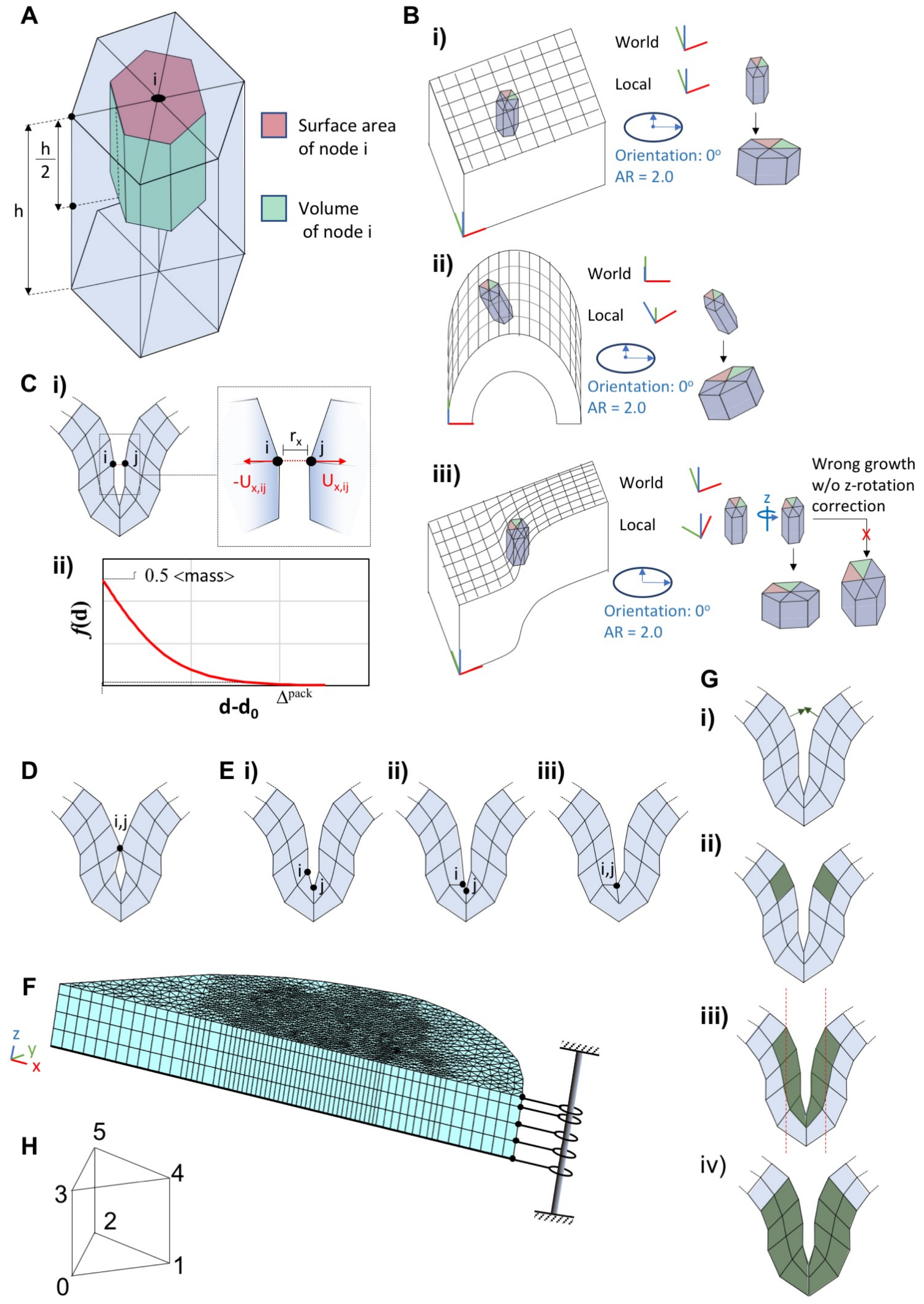

Supplementary Figure 1: Legend on next page.

Supplementary Figure 1: Related to figure 2. Modelling methodology details. A) Schematic for the externally exposed surface area and volume allocation to nodes. Node i is shared among six triangular prism elements, the total surface area highlighted in red, volume on green. B) Schematics for the oriented growth and related rotational corrections. A growth of doubling in volume, parallel to x-axis, with aspect ratio of 2 is demonstrated for all cases. All coordinate axes display x in red, y in green and z in blue. In the simulations, x is aligned with the DV axis, y with AP axis and z with AB axis of the tissue. i) Simple scenario where the world and local coordinates are aligned. ii) The elements have a rigid body rotation that deviates the local z-axis from the world coordinates, x & y are aligned. The growth follows local coordinate system, and the rigid body rotation is not accounted for. iii) The case where due to deformation in the tissue, the elements have gone through a rigid body rotation around the z-axis. The growth orientation is corrected for the rotation around z, and growth on x-y plane is applied in world coordinates. The wrong emergent grown shape in the case when this rotation was ignored is shown for comparison. C) Schematic for the hard wall potential applied to ensure volume exclusion. i) packing forces between nodes i and j in x-axis. The dashed box is enlarged on the inset, the calculated potential is applied in the opposite directions on both nodes. ii) The packing potential with distance displayed, the parameters defining the potential function are marked. D) Schematic displaying the adhesion of nodes i and j, the initial configuration same as Ci, the nodes are carried to the mid-point and their degrees of freedom bound. E) Schematic for node collapse on elements with nodes approaching within a small threshold distance of each other, implemented to limit element flipping. i) Node configuration outside collapse limit, ii) nodes moved within the collapse limit of each other due to viscoelastic system forces. iii) Configuration after node collapse. In D and E, the schematics are for demonstration purposes only and distances are not to scale. F) A sample simulated initial mesh, displaying the symmetricity assumption and showing the simulated half. Schematic added on the simulation mesh to demonstrate the no-bending boundary condition at circumference. G) Schematic demonstrating the algorithm to detect fold initiation with element surface normals. The detection is carried out on elements with exposed surfaces on either apical or basal surfaces, apical surface is utilised in demonstration. i) two normals on elements (green arrows) are within the vicinity of each other and the angle between the normals is wider than the selected threshold. ii) Two elements are assigned to be on fold initiation regions. iii) All elements that have their apical surfaces within the bounding box of the identified element couple are marked to be on fold initiation surface. For fold identification on the basal surface, the bounding box will check for basal surfaces of the remaining elements. iv) The fold initiation region is extended to cover the whole tissue thickness. H) The numbering of prism nodes in finite element formulations.

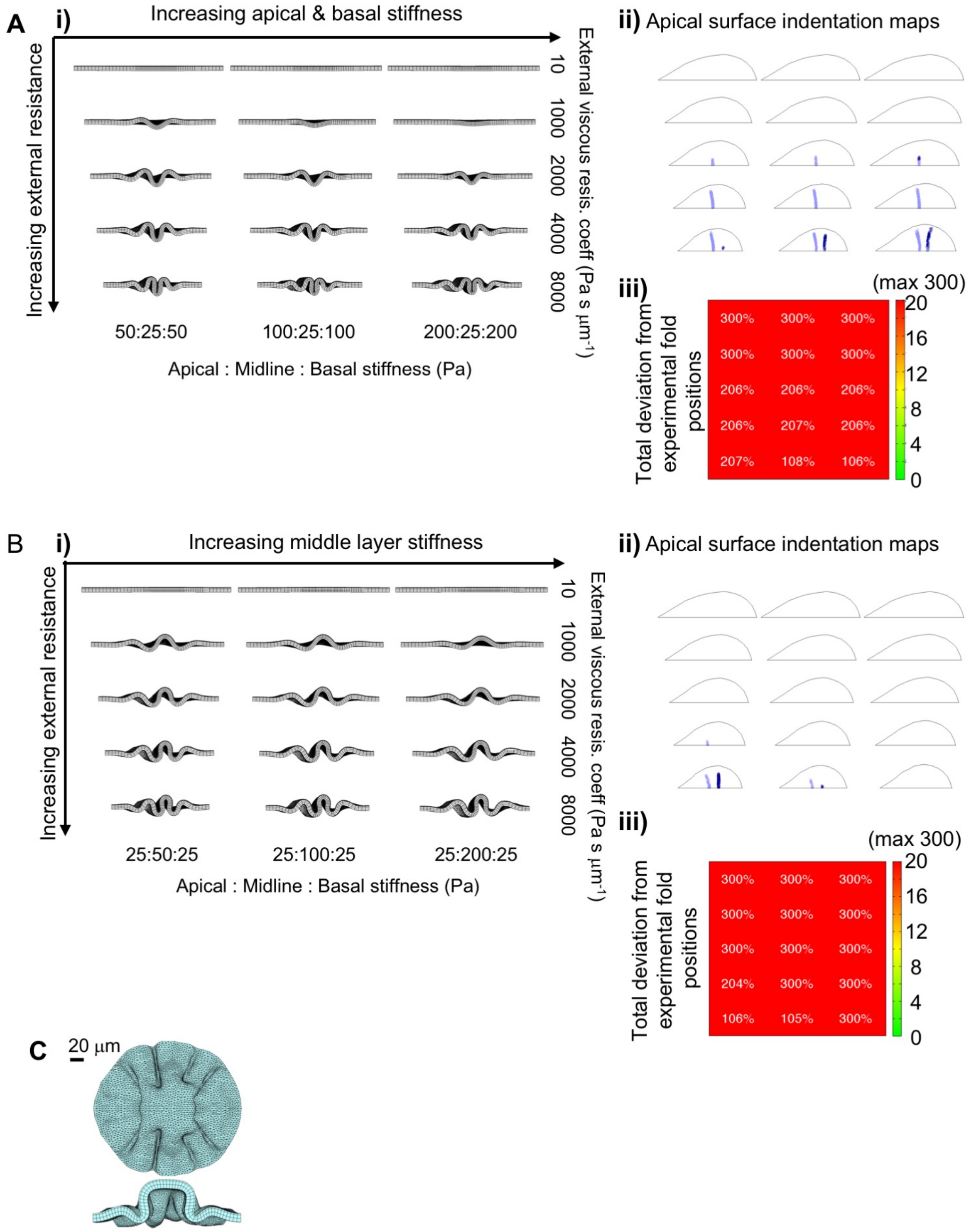

Supplementary Figure 2: Legend on next page.

Supplementary Figure 2: Related to Figure 3. Alternative relative stiffness states of the tissue. Tissue morphology with, A) increasing relative stiffness of apical and basal surfaces and external viscous resistance; B) increasing relative stiffness of tissue midline, and external viscous resistance. Each panel demonstrates simulation results for a tissue growing from 48 hour AEL to 96 hours AEL, with uniform in-plane growth rates, as stated in Figure 3A. Images are taken the cross-section of the tissue midline at 96 hours AEL, ventral tip on the right. Row and Column organisation same as Figure 3A. Both in A and B, ii) Apical indentation maps automatically identified from the curvature of facing surfaces, each continuous folding region is marked in a single colour. iii) Fold position deviations, calculated as sum of percentage deviation from each experimental fold at the tissue centre. Both i & ii calculated at same time points of (i), row column organisation is same as in (i). C) Simulation with same parameters as Figure 3B, on a symmetric, circular initial mesh. Snapshot is from 96 hours AEL, sagittal view as central cross-section, and dorsal tip to the right. Scale bar 20 μm.

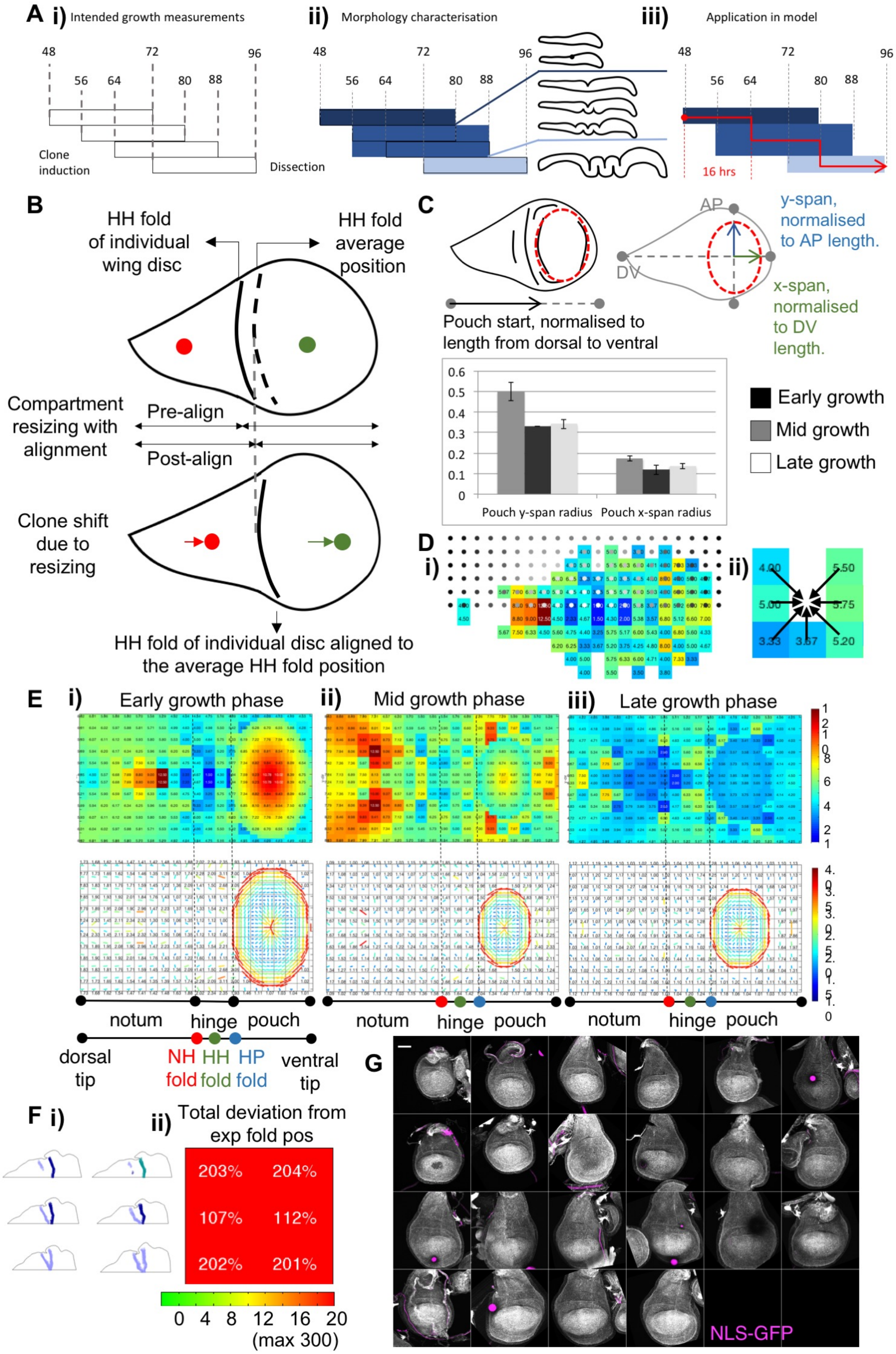

Supplementary Figure 3: Legend on next page.

Supplementary Figure 3: Related to Figure 4. Details of the growth rate analysis methodology. A) Definition of the growth phases. i) The targeted experimental timings. ii) The morphological staging of the dissected wing discs according to the folding stage. iii) The timing for the application of the growth phases in the simulations. B) Schematic representing the alignment of the growth rate measurements for a given time period. HH fold position (dashed line) is calculated as the average of all the wing-discs utilised in growth rate measurements for the selected time period. The HH position of each individual wing disc (solid line) is aligned to the average position. The two sides of the fold are rescaled to fit within the average size of the tissue, moving the data points (red on notum side, and green on pouch side) in the process. C) Measurements for the size of the pouch at different time periods within 48 to 96 hours AEL. Top schematics demonstrate how the pouch position and size are normalised. Plot demonstrates means with whiskers on one standard deviation. D) The order in which the grid points are checked, and filled as necessary. i) Order goes from light to dark shades of dots on grid points. ii) Once an empty grid point (with no experimental data points) is reached, the existing data in immediate neighbours are averaged to cover the empty point. The order of sampling for empty grid points is of significance as filled regions contribute to the filling of their neighbours, thus enabling us to fill the extended patches of empty regions on the grid. E) The extended version of growth maps demonstrated in Figure 4B. Colour bars on the right hand side of the panes are valid for all. The measured fold positions, and the corresponding tissue compartments are marked on the heatmaps, the positions are measured for 72-88 hours AEL in i&ii, for 96 hours AEL in iii. F) i) Apical indentation maps and ii) fold position deviations of simulations with experimental growth rate, as presented in Figure 4C. G) Non-heat shock controls, NLF-GFP in magenta. No spontaneous expression is observed in wing disc columnar epithelium. Scale bars 50 μm

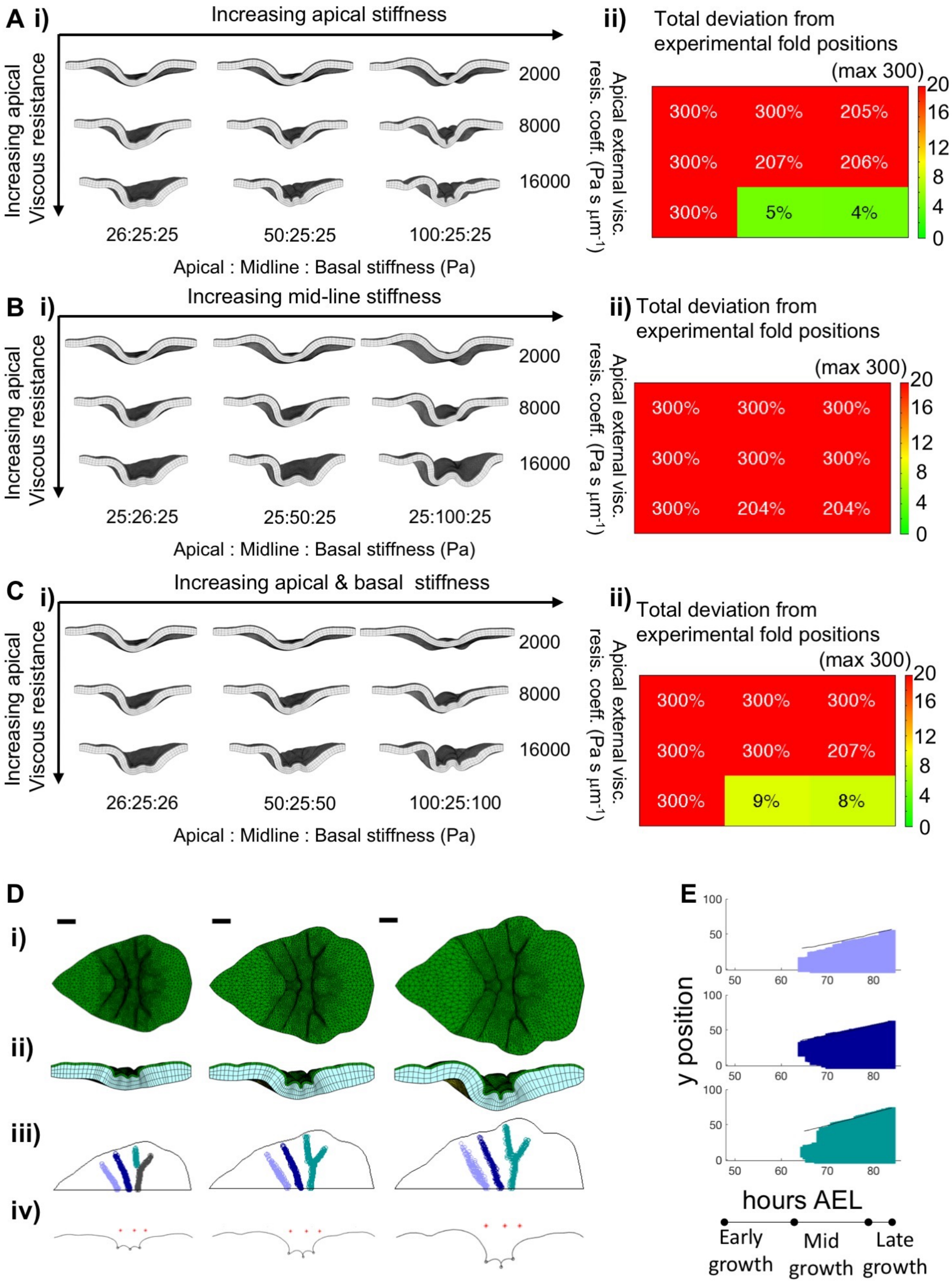

Supplementary Figure 4: Legend on next page.

Supplementary Figure 4: Related to Figure 5. A) Simulations with apical surface is stiffer than the rest of the cell body. i) The effects of apical viscous resistance coefficient and tissue stiffness heterogeneity on emergent morphology, columns: increasing apical viscous resistance coefficient, rows: increasing relative stiffness of apical surface. Simulation in lower right corner is detailed in Figure 5D. ii) Fold position deviations, calculated as sum of percentage deviation from each experimental fold at the tissue centre, row column organisation is same as in (i). The tissue has explicit BM definition at 1600 Pa stiffness and renewal half-life of 8 hour, basal viscous resistance coefficient is 10 Pa s μm^{-1}. B) Simulations with tissue midline stiffer than the rest of the tissue, grid organisation and all remaining simulation parameters are same as (A). C) Simulations with tissue apical and basal surfaces stiffer than the rest of the cell body, grid organisation and all remaining simulation parameters are same as (A). D) Timeline of z-growth added simulation in Figure 5G and Movie 5. Snapshots are from 72, 78, and 84 hours AEL, respectively. Scale bar is 20 μm. i) top view, ii) cross-section view, iii) apical indentation maps. iv) The positions of the folds on the tissue cross section apical surface profile, the red stars mark the experimental fold positions measured for 72-88 hours AEL (Fig. 1Ci). E) Kymographs of apical indentations in time. Y position of all nodes falling into the three major folds at 84 hours AEL are plotted in time. Colour coding same as (Diii) at 84 hours AEL.

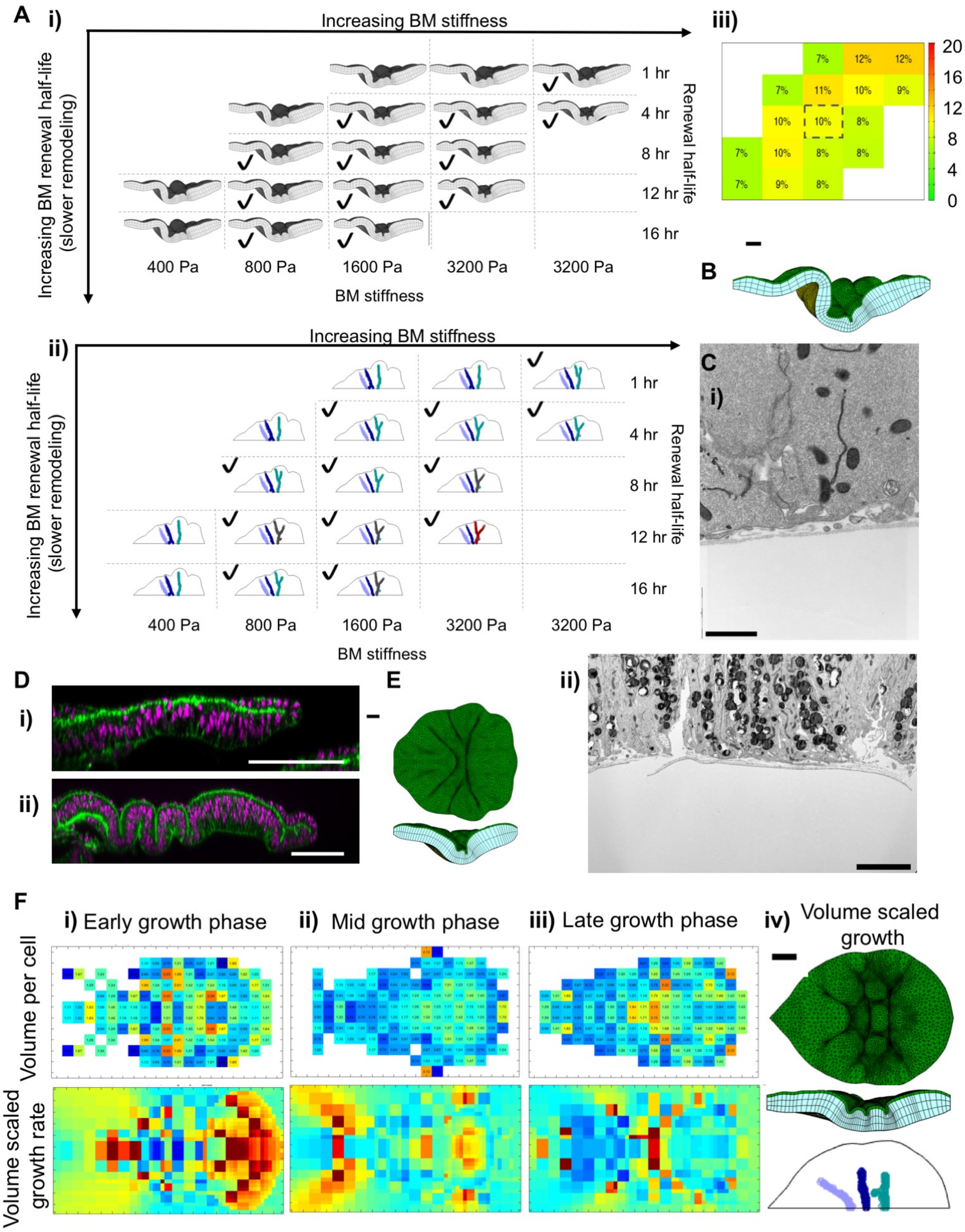

Supplementary Figure 5: Legend on next page.

Supplementary Figure 5: Related to Figure 5. A) The effects of BM stiffness and BM renewal half-life on emergent morphology. Rows, increasing BM stiffness, columns increasing renewal half-life (slower remodelling). i) Sagittal views displayed for each parameter combination at 84 hr AEL. ii) apical indentation maps. Acceptable parameter combinations marked with a green tick in (i & ii). Failing simulations form a forked HH fold, merging with the NH fold at tissue midline. iii) Percentage deviation of fold positions from the experimental positions measured at tissue midline at 36 hr AEL. The simulation presented in Figure 5G is marked by the dashed rectangle. B) The late stages of the simulation in Figure 5G, at 96 hours AEL. The initiated folds do not successfully progress into a fully folded morphology. The tissue goes through large scale buckling, the HH fold is opened up and hinge sinks well below the notum in z. Scale bar, 20 μm. C) The larger field view images of EM images (Figure 5A), i) 72 hr AEL, scale bar 1 μm, ii) 120 hr AEL, scale bar 5 μm. D) The larger field of view images where the pseudostratification images are taken from i) Figure 5Fiv-v, ii) Figure 5Fvi-vii. Scale bars 50 μm. E) Simulation on a circular initial tissue shape, with parameters same as Figure 5G. Scale bar 20 μm. F) i-iii)Normalised cell volume maps in three growth phases (top) and the growth rates scaled with the cell volume (bottom). iv) Simulation with volume scaled growth rates, snapshots are from 78 hr AEL, top panel: simulation snapshot from top view, middle: simulation snapshot from cross-section of the midline, bottom: fold initiation map of the simulation. Scale bar is 20 μm. The physical properties and boundary conditions of simulations in E/G-H are the same as presented on Figure 5D.

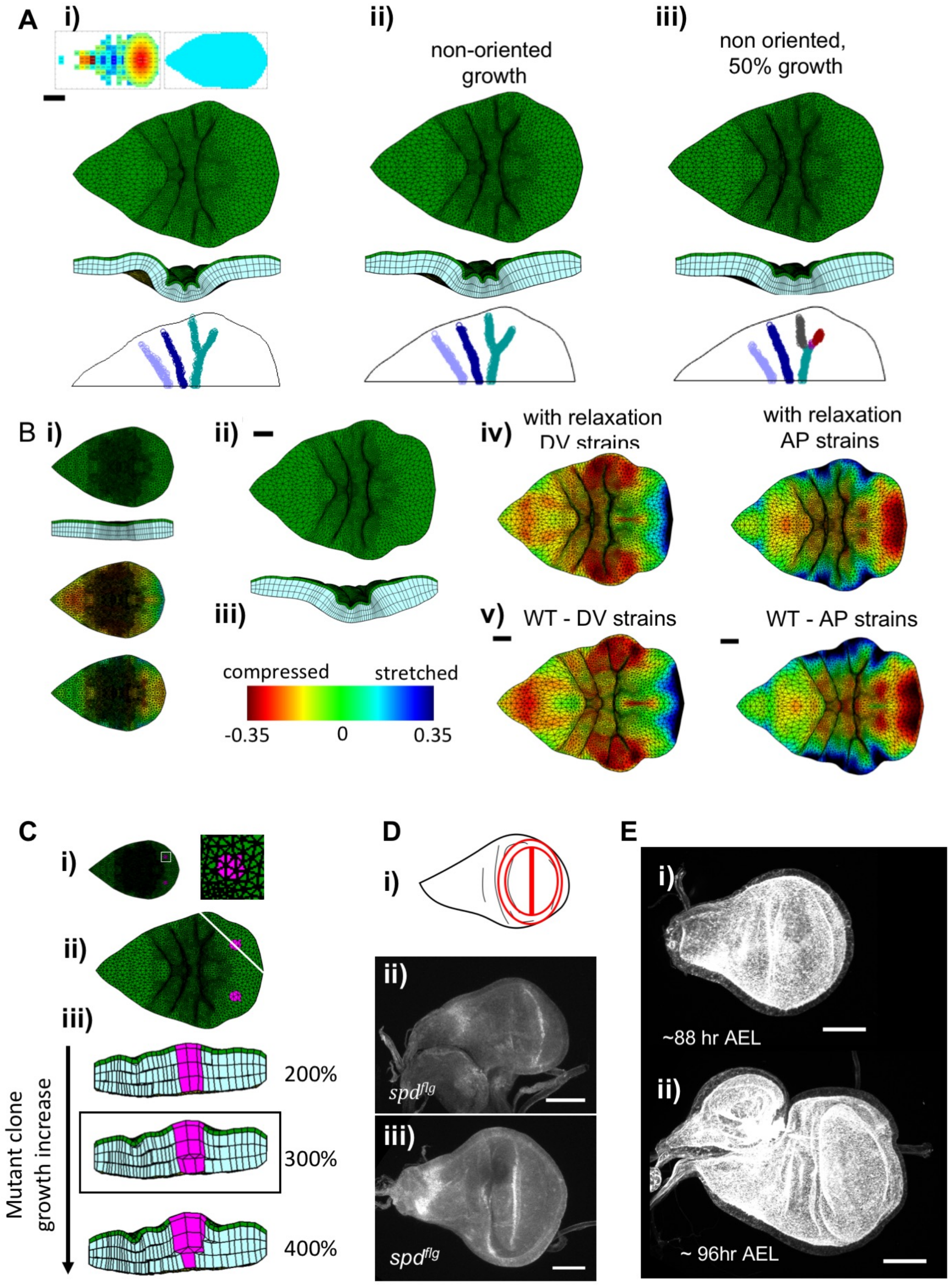

Supplementary Figure 6: Legend on next page.

Supplementary Figure 6: Related to Figure 6. The differential growth in early growth phases and related force accumulation is necessary and sufficient for correct morphology in overgrowth clones, wild type and mutant wing discs. A) i) Early growth rates and orientations applied for the initial 16 hours (48 to 64 hours AEL) of simulation, continued by uniform growth. Top panel: The growth maps applied, colour coding same as Figure 4B. Middle panel: Simulation snapshots from 84 hours AEL, top and sagittal view, scale bar 20 μm. Bottom panel: Apical indentations map at tissue at 84 hours AEL, scale same as simulation snapshots. ii) Early growth rates and orientations applied for the initial 16 hours (48 to 64 hours AEL) of simulation, continued by experimental growth rates without any orientation. Top panel: Simulation snapshots from 84 hours AEL, top and sagittal view. Bottom panel: Apical indentations map at tissue at 84 hours AEL, scale same as simulation snapshots. iii) Same as (ii), but the growth rates are also reduced to 50 percent of experimentally measured after the initial 16 hours. All simulation physical parameters are same as 5D. B) Simulation with experimental growth rates, with all accumulated forces relaxed at 58 hours AEL (10 hours into simulation). i) Top panels: Tissue morphology at 58 hours AEL, immediately prior to relaxation of forces. Middle panel: Strains accumulated in DV orientation, and bottom panel: strains on AP orientation, colour coding in (iii). ii) Simulation snapshots at 84 hours AEL, following the relaxation of forces at 58 hr AEL. iv) Strains of (ii), left: strains accumulated in DV orientation, right: strains on AP orientation, colour coding in (iii). v) Accumulated strains in wild type simulation, in Fig. 5G, panel structure and colour coding same as (iv). C) The range of tested overgrowth in clones, i) snapshot at the onset of simulation (48 hr AEL), with the induced clone (magenta) within white rectangle enlarged on the right. Clone diameter approximately 4 micrometers. ii) Simulation snapshot, top view from 75 hr AEL, for 300 percent growth in clone (corresponding to Fig. 6D). The while line marks the line of cross-section represented in (iii). iii) Simulation snapshots demonstrating ectopic fold emergence as the overgrowth is increased. The setup demonstrated in Figure 6 B is boxed. Simulation snapshots from 75 hr AEL unless stated otherwise on the image, all physical properties same as Fig. 5D. D) i) Schematic marking the pattern of *wingless* expression in wild type wing discs. The inner ring appears prior to fold formation in early third instar, followed by the outer ring in late third instar. ii) *Wingless* staining in spd^{fg}, prior to formation of folds, the stage where the inner ring should have appeared. iii) Wingless staining in late third instar mutant wing disc, showing the outer ring of expression, and lacking the inner ring. Scale bars 50 μm. E) The top views maximum projection images of the experimental spd^{fg} mutants shown in Figure 6I, Scale bars 50 μm

Method S1: Methodology for the computational model, related to STAR Methods

1 Introduction

This methods section describes our finite element model of tissue morphogenesis. In our model, the tissue is treated as a continuous material. The discretisation is based on 6-node prism elements, that can grow and vary in size from subcellular to multicellular (Fig. 2A-C, S1A). The driving force behind the shape change of the tissue is this growth of the elements. Emergent nodal positions, thus the tissue morphology, is governed by the balance of the elastic forces and external viscous resistance to movement. The discrete form of balance equations give rise to a system of non-linear equations, that are linearised and solved numerically with a Newton-Raphson method.

The stress-strain relationship of the tissue is represented by a Neo-Hookean material model. The sub-compartments of the tissue utilise different set of parameters, but satisfy the same governing equations, for example the basement membrane has relatively higher stiffness. When applied, the external viscous resistance is proportional to the exposed surface area and velocity. The parameters utilised through the manuscript are summarised in Supplementary Table 3.

The initial geometry is defined by tessellation of the contour of a wing disc at 48 hours AEL using the Trianlge software (Shewchuk, 2005). The contour is scaled to the average dimensions (Fig. 1C, 2E). We assume anterior-posterior symmetry and simulate half of the tissue. As such, the simulations are run with fixed y-position (along AP axis) for all nodes at the dorsal-ventral plane of symmetry, that is at the midline of the tissue. The outer boundary conditions at the circumference of the tissue limit bending, such that all nodes on the same column at the boundary have the same x & y coordinates (Fig. S1F). During simulations, the tissue surface can form adhesions. Volume exclusion is ensured by a hard-wall potential and element flipping is avoided by collapsing nodes on single elements when the edge of the element is below a set threshold (Fig. S1C-E).

2 Modelling methodology

2.1 Equilibrium equation and finite element discretisation

The tissue is modelled as a deformable body occupying a time varying domain Ω_t. We denote by $\boldsymbol{x}(t)$ the current positions of the material point that is initially at position $\boldsymbol{X}$. After neglecting body loads (gravity) and inertial terms, the equilibrium equations of Ω_t are given by Cauchy's equations and appropriate boundary conditions:

$$\begin{aligned} \nabla \cdot \boldsymbol{\sigma} &= \boldsymbol{0}, \ \forall \boldsymbol{x} \in int(\Omega_t) \\ \boldsymbol{\sigma n} &= \bar{\boldsymbol{t}}, \ \forall \boldsymbol{x} \in \Gamma_n \\ \boldsymbol{x} &= \bar{\boldsymbol{x}} \ \forall \boldsymbol{x} \in \Gamma_x, \end{aligned} \tag{1}$$

with $\bar{\boldsymbol{t}}$ and $\bar{\boldsymbol{x}}$ prescribed loads and positions at boundaries Γ_n and Γ_x, respectively, which will specified later, and $\boldsymbol{n}$ the external normal.

The finite element formulation is obtained by a standard construction of the equivalent weak form (Bonet and Wood, 2008), which reads,

$$\int_{\Omega_t} \boldsymbol{d}(\delta\boldsymbol{v}) : \boldsymbol{\sigma} dV_t = \int_{\Gamma_n} \delta\boldsymbol{v} \cdot \bar{\boldsymbol{t}} dS_t, \forall \delta\boldsymbol{v} \in H_0 \tag{2}$$

The vector $\delta\boldsymbol{v}$ is a test function belonging to appropriate Hilbert space H_0 of functions with bounded integrals, and the strain tensor $\boldsymbol{d}(\delta\boldsymbol{v}) = \frac{1}{2}\left(\nabla_x \delta\boldsymbol{v} + (\nabla_x \delta\boldsymbol{v})^T\right)$ is a measure of the body deformation.

The finite element discretisation is achieved by introducing an interpolation of the positions $\boldsymbol{x}(t) \approx N_j(\boldsymbol{x})\boldsymbol{x}_j(t)$ and the test functions $\delta\boldsymbol{v} \approx N_i(\boldsymbol{x})\delta\boldsymbol{v}_i$ with a set of complete shape functions $N_i(\boldsymbol{x})$ (Supplementary Table 1) and where $\boldsymbol{x}_j(t)$ are time varying nodal positions. In our case, we use six noded elements forming a triangular prism aligned along the apical-basal axis (see Figure S1H), so that positions are interpolated linearly along each element height, and bi-liniearly at each element cross-section. Then within each element, a numerical approximation is carried out with six Gauss points quadrature (Supplementary Table 2).

The imposition of the discretised version of the weak form in (2) leads to the following system of equations (see Bonet and Wood, 2008 for a detailed derivation):

$$\boldsymbol{g}(\boldsymbol{x}, t) \equiv \boldsymbol{g}^{elast}(\boldsymbol{x}, t) - \boldsymbol{g}^{ext}(\boldsymbol{x}, t) = \boldsymbol{0} \tag{3}$$

where for each node i, the elastic and external residual contributions are given by,

$$\boldsymbol{g}_i^{elast} = \int_{\Omega_t} \boldsymbol{\sigma} \nabla_x N_i dV_t \tag{4}$$

$$\boldsymbol{g}_i^{ext} = \int_{\Omega_t} N_i \bar{\boldsymbol{t}} dS_t \tag{5}$$

Here, and in the subsequent derivations, we use $\boldsymbol{x}$ to represent $\boldsymbol{x}\,(t)$ for clarity. This nodal contributions are computed element-wise and assembled in the standard manner in the finite element context (Bonet and Wood, 2008).

As it will be described below, the stress tensor $\boldsymbol{\sigma}$ follows a non-linear constitutive law that varies along time, while the external forces in $\boldsymbol{g}^{ext}$ includes time dependent viscous forces. As a result, and due to the presence of large deformations, the set of non-linear equations in (3) is discretised in time using a backward Euler implicit scheme and solved iteratively at each time-step with a Newton-Raphson process in order to achieve quadratic convergence. This process requires the linearisation of the residual $\boldsymbol{g}(\boldsymbol{x}_n, t_n)$ at each time-step t_n. At iteration k, the new iterative changes of the displacements $\delta\boldsymbol{x}$ are found by solving the following linear equations:

$$\boldsymbol{g}(\boldsymbol{x}_n^k, t_n) + \boldsymbol{K}\delta\boldsymbol{x} = \boldsymbol{0} \;\rightarrow\; \boldsymbol{x}_n^{k+1} = \boldsymbol{x}_n^k + \delta\boldsymbol{x}. \tag{6}$$

The Jacobian $\boldsymbol{K} = \frac{\partial \boldsymbol{g}}{\partial \boldsymbol{x}}$ is also updated at each iteration from $\boldsymbol{x}_n^k$ and $\boldsymbol{x}_{n-1}$. Its expression will be specified below.

2.2 Deformation gradient decomposition and elastic constitutive law

The elastic constitutive law requires the computation of the total deformation gradient $\boldsymbol{F} = \frac{\partial \boldsymbol{x}}{\partial \boldsymbol{X}}$, which is decomposed into elastic and growth components,

$$\boldsymbol{F} = \boldsymbol{F}^e \boldsymbol{F}^g \tag{7}$$

The stress tensor $\boldsymbol{\sigma}$ is assumed to follow a compressible Neo-Hookean non-linear constitutive law that solely depends on the elastic component $\boldsymbol{F}^e$ through an elastic density function (Bonet and Wood, 2008)

$$W(\boldsymbol{C}^e) = \frac{\mu}{2}(trace(\boldsymbol{C}^e) - 3) - \mu \ln J^e + \frac{\lambda}{2}(\ln J^e)^2 \tag{8}$$

with λ and μ constant material parameters, $\boldsymbol{C}^e = \boldsymbol{F}^{eT}\boldsymbol{F}^e$ the right Cauchy-Green strain tensor, and $J^e = \det(\boldsymbol{F}^e) = \sqrt{\det(\boldsymbol{C}^e)}$. The Cauchy stress is then given by

$$\boldsymbol{\sigma} = J^{e-1}\boldsymbol{F}^e \boldsymbol{S} \boldsymbol{F}^{eT} \tag{9}$$

with $\boldsymbol{S} = 2\frac{\partial W(\boldsymbol{C}^e)}{\partial \boldsymbol{C}^e}$ the second Piola-Kirchhoff stress tensor:

$$\boldsymbol{S}^e = \mu(\mathbf{I} - \mathbf{C}^{-1}) + \lambda(lnJ^e)\mathbf{C}^{-1} \tag{10}$$

Note that the computation of the stresses from the current deformation $\boldsymbol{F}$ depends on the growth component $\boldsymbol{F}^g$, which as will shown below is time dependent.

From the expression of $\boldsymbol{g}_i^{elas}$ in equation (4) and the definition of the elasticity function and stresses through equations (8)-(9), the following contribution to the Jacobian, coupling nodes i and j can be derived

$$\mathbf{K}_{ij}^{elast} = \frac{\partial \boldsymbol{g}_i^{elast}}{\partial \boldsymbol{x}_j} = \int_V \boldsymbol{B}_i^T \boldsymbol{F}\{\mathcal{C}\}\boldsymbol{F}^T \boldsymbol{B}_j dV + \mathbf{I}\int_V \nabla_x N_i^T \boldsymbol{\sigma} \nabla_x N_j dV. \tag{11}$$

with $\boldsymbol{B}_i$ a deformation matrix that allows to compute the deformation rate as $\boldsymbol{d}(\delta\boldsymbol{x}) = \boldsymbol{B}_i \delta \boldsymbol{x}_i$, and $\{\mathcal{C}\}$ the matrix notation of the fourth order elasticity tensor $\mathcal{C}$,

$$\mathcal{C}_{ijkl} = \lambda \boldsymbol{C}_{ij}^{-1}\boldsymbol{C}_{kl}^{-1} + 2\left(\mu - \lambda ln(J)\right)\mathcal{I}_{ijkl} \tag{12}$$

where $\mathcal{I}_{ijkl}$ is given by

$$\mathcal{I}_{ijkl} = \frac{1}{2}\left[\boldsymbol{C}_{ik}^{-1}\boldsymbol{C}_{jl}^{-1} + \boldsymbol{C}_{il}^{-1}\boldsymbol{C}_{jk}^{-1}\right]. \tag{13}$$

2.3 External force contribution

We assume that the external forces at the boundary Γ_n correspond to a viscous loading due to friction with the external environment. The nodal drag forces are consequently given by

$$\boldsymbol{g}_i^{ext} = \int_{\Gamma_n} \eta_{ext} N_i \boldsymbol{v} dS_t$$

with η_{ext} the external viscous resistance coefficient, and $\boldsymbol{v}$ the velocity at the boundary, the boundary domain being the externally exposed apical and basal surfaces of the tissue..

This resistance is defined on a nodal basis and is heterogeneous within different regions of the tissue, having most significant influence on the dynamics of the apical surface (Supplementary Table 3). Both the velocity and the boundary Γ_n are time-dependent. In order to reduce the computations and the linearisation needed for the Newton-Rapshon solution, the nodal contribution is approximated at time t_n as

$$\boldsymbol{g}_{i,n}^{ext} = \int_{\Gamma_n} \eta_{ext} N_i \boldsymbol{v} dS_t \approx \eta_{ext} A_{n-1,i} \frac{\boldsymbol{x}_n - \boldsymbol{x}_{n-1}}{\Delta t}$$

with $\Delta t = t_n - t_{n-1}$ the time-step size, and $A_{n-1,i}$ the area attributed to node i at time t_{n-1}, which is computed as,

$$A_{n-1,i} = \sum_{e=1:n_{owner}} \frac{A_{n-1,e}}{n_{e,surf}} \tag{14}$$

Here, n_{owner} is the number of elements connected to node i, $A_{n-1,e}$ is the exposed surface area of interest on element e at time t_{n-1}, and $n_{e,surf}$ is the number of nodes that element e has on its exposed surface of interest (Fig. S1A). For instance, for prisms of 6 nodes, the number of nodes associated with the apical surface would be 3. From the expression of $\boldsymbol{g}_i^{ext}$, the following contribution of the external forces to the Jacobian can be deduced:

$$\boldsymbol{K}_{ij}^{ext} = \frac{\eta_{ext} A_{n-1,i}}{\Delta t} \mathbf{I}.$$

2.4 Calculation of growth

The growth rates are input to the simulations in the form of the experimental growth maps of Figure 4. To obtain the local growth rate of a single element, first the relative position of the element centre in the xy-plane bounding box of the tissue is calculated. Then the growth rate, $[r_x, r_y, r_z]$, and the growth orientation angle, θ_g, is interpolated from the nearest corners of the input growth map grid (Figure 2 for experimental measurements). This growth is then incorporated into the element by a multiplicative decomposition in equation (7): $\boldsymbol{F} = \boldsymbol{F}^e \boldsymbol{F}^g$ (see Fig. 2B). Consequently, $\boldsymbol{F}^g$ must be updated at each time step Δt, depending on the input growth rates, growth orientation, and the current rigid body rotations of the element according to,

$$\boldsymbol{F}_{t+\Delta t}^g = \boldsymbol{R}_{er} \boldsymbol{R}_{gr} \begin{bmatrix} e^{r_x \Delta t} & 0 & 0 \\ 0 & e^{r_y \Delta t} & 0 \\ 0 & 0 & e^{r_z \Delta t} \end{bmatrix} \boldsymbol{R}_{gr}^T \boldsymbol{R}_{er}^T \boldsymbol{F}_t^g. \tag{15}$$

Here the growth rates are in the local coordinates of the element, $\boldsymbol{R}_{gr}$ is the rotation matrix for the growth orientation angle (θ_g) and $\boldsymbol{R}_{er}$ is the rotation matrix associated with the current elemental rotation in the plane of the tissue. $\boldsymbol{R}_{gr}$ is simply the rotation around the z-axis with the input growth orientation angle θ_g.

This growth orientation angle θ_g in simulation inputs is calculated from the maximum projection of the experimental images on the xy-plane. To match this methodology, the current rotation of the element around the z-axis is corrected in order to ensure that the orientation of the growth follows the xy-plane of the tissue. At the beginning of the simulations, the local coordinate system of each element is aligned with the world coordinate

system. During the simulation, the local coordinate system of the element could deviate from the world coordinates, due to rigid body rotations, imposed by the deformations of the surrounding tissue (Fig. S1B). Any rotation around the z-axis, changing the xy-plane of the element, should be accounted for, so that the element will continue growing on the desired orientation in the world coordinates. On the other hand, the tilt of the z-axis itself should be ignored; an element with tilted apical-basal (AB) axis should not start elongating in the AB direction (Fig. S1B). To obtain the rotation matrix $\boldsymbol{R}_{er}$ first the current rigid body rotation of the element is calculated from the deformation gradient via single value decomposition such that:

$$\boldsymbol{F} = \boldsymbol{V}\boldsymbol{P}\boldsymbol{U}^T. \tag{16}$$

Then the rotation matrix corresponding to the rigid body rotation of the deformed element can be obtained from:

$$\boldsymbol{R}_{rigid} = \boldsymbol{V}\boldsymbol{U}^T \tag{17}$$

and the angle of rotation around the z axis is extracted from the calculated rigid body rotation matrix from:

$$\theta_z = \arctan\left(\boldsymbol{R}_{rigid}\left(0,1\right), \boldsymbol{R}_{rigid}\left(0,0\right)\right) \tag{18}$$

where

$$\boldsymbol{R}_{rigid} = \boldsymbol{R}_x\left(\theta_x\right)\boldsymbol{R}_y\left(\theta_y\right)\boldsymbol{R}_z\left(\theta_z\right). \tag{19}$$

The elemental rotation matrix $\boldsymbol{R}_{er}$ corrects for the rotation in z, as such, $\boldsymbol{R}_{er}$ is rotation around z axis by $-\theta_z$.

2.5 Calculation of remodelling

While the cellular elements of the tissue grow with specified growth rates and orientations, the basal membrane (BM) grows by remodelling. The application of remodelling follows the logic of equation (15), with the growth increment and related rotations defined from deformations, rather than an input growth profile. As such, the rigid body rotation correction, $\boldsymbol{R}_{er}$, is the identity matrix for BM remodelling, and the equivalent of growth orientation, $\boldsymbol{R}_{gr}$, is obtained through the elastic deformation orientation. In the generalised definition, the remodelling growth at each time step is obtained via Eigen value decomposition of the Cauchy-Green strain matrix $\boldsymbol{E} = \frac{1}{2}\left(\boldsymbol{C} - \boldsymbol{I}\right)$ of the element, and the deformations on the principal axes are calculated via equation,

$$F_{kk} = \sqrt{2e_k + 1} \tag{20}$$

Here, F_{kk} is the current deformation along the principal axis k, (such that a 50% stretch will give a value of 1.5), and e_k is the kth Eigen value of $\boldsymbol{E}$. In the specific case of basement membrane remodelling, as the BM is stretched there is no evidence that the BM should be getting thinner. On the contrary, BM does get thicker with age as demonstrated in our quantification of EM images of wing disc BMs at 72 and 120 hr AEL (Figure 5A). Moreover, as the remodelling in the simulation is based on the strains on the elements, while the BM is deformed and the new BM is allocated, the BM thickness should not be

reduced with remodelling. As such, remodelling is limited to plane of the tissue. BM is not remodelled in the apical-basal axis, only the x & y dimensions of the strain matrix are included in the decomposition. Here, the range of k in equations (20) - (22) are limited to $k = 1 : 2$ in 2D, excluding local z coordinates of the element, which is aligned with apical-basal axis. Similarly, the Eigen value decomposition is also carried out on the x-y plane of the Cauchy-Green strain matrix $\boldsymbol{E}$. The deformation after the relaxation to be observed within the current time step Δt is then calculated from the given remodelling half-life, $t_{1/2}$ as follows:

$$F_{kk}^{t+\Delta t} = \left(F_{kk}^{t} - 1\right)\left(\frac{1}{2}\right)^{\Delta t/t_{1/2}} \tag{21}$$

The calculated new deformation is then converted to a growth increment, with the orientation of the growth defined by the eigenvectors matrix $\boldsymbol{V}_{Eigen}$ to give the remodelling deformation gradient.

$$\boldsymbol{F}^{r} = \boldsymbol{V}_{eigen} \begin{bmatrix} F_{11}^{t+\Delta t}/F_{11}^{t} & 0 & 0 \\ 0 & F_{22}^{t+\Delta t}/F_{22}^{t} & 0 \\ 0 & 0 & 1 \end{bmatrix} \boldsymbol{V}_{eigen}^{T} \tag{22}$$

The remodelling serves to mimic BM remodelling carried out by the cells as the tissue grows, adding or removing material from the BM layer as needed. Therefore, remodelling of BM is done without volume conservation (determinant of $\boldsymbol{F}_r$ can deviate from unity). At each time step, the remodelling growth increment $\boldsymbol{F}_r$ is added to total growth deformation gradient $\boldsymbol{F}^g$,

$$\boldsymbol{F}^{g} = \boldsymbol{F}^{r}\boldsymbol{F}^{g'} \tag{23}$$

where $\boldsymbol{F}^{g'}$ is the growth increment of the current step prior to remodelling as calculated through equation (15). For a simplified visual representation of the emergent remodelling, the relaxation of deformation is represented in a 2D schematic in Figure 5C, where upon change of the current shape of an element (blue square, Fig. 5Ci-ii), the preferred shape (red dashed square) resulting from application of remodelling on reference shape gradually changes and aligns with the current shape (Fig 5Cii-iii), relaxing the strains in the process (Fig. 5Civ).

2.6 Node-node interactions

Nodes can interact with each other via a packing hard-wall potential to simulate tissue self-contact, or through adhesions. When the two surfaces of the tissue are in close vicinity, they form adhesions, that is, nodes are joined together and are collapsed to a single node. Packing forces are used instead for cases where adhesion between the nodes would cause the shared element to flip. In cases where the nodes of an element are approaching too close to each other, such as the case of a highly constrained apical surface at a fold, the nodes of the element are also collapsed, in order to avoid element flipping.

2.6.1 Calculation of packing hard-wall potential

The hard wall potential is defined to simulate contact and ensure volume exclusion as the elements move too close to each other (Fig. S1C). The potential is applied on a nodal

basis. The threshold of repulsion force application, Δ^{pack}, is dynamic in the simulations, scaling to the average side length of an element in the vicinity of potential node-node interaction. The threshold in the simulations throughout the manuscript is defined to be 40% of the average local side length, calculated on a 10 by 5 grid on the tissue xy bounding box. The parameters of the hard-wall potential are selected numerically in order to ensure volume exclusion and stability, not necessitating a biological basis, the values are presented in Supplementary Table 3.

The magnitude $f(d)$ of the applied packing force is a function of the distance d between nodes, and is calculated with an inverse logic function:

$$f\left(d\right) = \frac{\langle mass \rangle}{1 + e^{-k(d-d_0)}} \tag{24}$$

Here, the amplitude $\langle mass \rangle$ is defined such that the force will scale with the average mass of the packing node couple. The mass itself is taken proportional to the volume associated to each node. The slope of the curve in the force profile will tend to zero as the distance between nodes approaches the packing threshold Δ^{pack}. Parameter k of the sigmoid function in (24) is defined by the saturation term s^{sat} and the packing threshold Δ^{pack} as

$$-k = \frac{2\, s^{sat}}{\Delta^{pack}} \tag{25}$$

The packing threshold Δ^{pack} is dependent on the current average side lengths of mesh elements. The sigmoid saturation is set to 5, as this is the approximate saturation distance of the standard logistic function. The distance is shifted with distance d_0 to move the mid point of the function to approximately 60% of the packing threshold distance. The forces between each pair of nodes i and j is computed from $f(d)$ as,

$$\boldsymbol{f}_i(d) = f(d)\, \boldsymbol{e}_i \ , \ \boldsymbol{f}_j(d) = f(d)\, \boldsymbol{e}_j = -f(d)\, \boldsymbol{e}_i = -\boldsymbol{f}_i \tag{26}$$

with $d = ||\boldsymbol{x}_i - \boldsymbol{x}_j||$ and $\boldsymbol{e}_i = \left(\boldsymbol{x}_i - \boldsymbol{x}_j\right)/d$. The forces $\boldsymbol{f}_i$ and $\boldsymbol{f}_j$ are added to the global residual vector $\boldsymbol{g}$ in (3), and the term

$$\frac{\partial \boldsymbol{f}_i}{\partial \boldsymbol{x}_j} = \frac{f(d)}{d}\left(\mathbf{I} - \boldsymbol{e}_i \boldsymbol{e}_j^T\right) + f'(d)\boldsymbol{e}_i \boldsymbol{e}_j^T,$$

with $f'(d) = -2\,\langle mass \rangle\, s^{sat} f(d)(1 - f(d))/\Delta^{pack}$, is also added to the corresponding term $\boldsymbol{K}_{ij}$ in the Jacobian during the Newton-Raphson solution process.

2.6.2 Calculation of node binding for node adhesion, elemental collapse and boundary conditions

Nodes within a close vicinity can bind to each other (Fig. S1D). This can be due to adhesion of two surfaces in the case of nodes that are not shared among any elements, or due to the collapse of an elemental surface. Adhesion is defined by moving both nodes to the mid-point, and collapsing their degrees of freedom. The two nodes are assigned master and slave status arbitrarily. All the driving and drag forces of the slave node are carried on to the master node, and the Jacobian is updated accordingly. Upon obtaining the displacements, the displacement of the slave node is updated with the displacement

of the master node. This is equivalent to a master-slave treatment of the nodal constraint $\boldsymbol{x}_{slave} = \boldsymbol{x}_{master}$ (see for instance (Muñoz, J.J. and Jelenić, 2004).

The distance threshold for adhesion is calculated the same manner as the hard wall potential. The threshold for element collapse is stricter than adhesion; defined on an elemental basis, as a distance below 10% of the initial reference length between the two potentially collapsing nodes, as opposed to being proportional to the current average side length of the system (Fig. S2E). The residual nodal forces $\boldsymbol{g}$ and Jacobian $\boldsymbol{K}$ are then modified as follows:

$$\boldsymbol{K}_{bound} = \boldsymbol{N}^T \boldsymbol{K}\boldsymbol{N} + \bar{\boldsymbol{I}} \tag{27}$$

$$\boldsymbol{g}_{bound} = \boldsymbol{N}^{\boldsymbol{T}} \boldsymbol{g} \tag{28}$$

$$\boldsymbol{N}_{ij} = \begin{cases} 1 & if \quad i = j \text{ and } i \text{ is not a slave} \\ 1 & if \qquad\quad i \text{ is slave to } j \\ 0 & \qquad\qquad \text{elsewhere} \end{cases} \tag{29}$$

$$\bar{\boldsymbol{I}}_{ij} = \begin{cases} 1 & if \quad i = j \text{ and } i \text{ is a slave} \\ 0 & \qquad\qquad \text{elsewhere} \end{cases} \tag{30}$$

The same master slave definition is used for no-bending boundary condition. At the circumference, the basal node of each column of nodes is assigned as the master of all the remaining nodes of the column, and the degrees of freedom in x and y directions of slaves are fixed on the master (Fig. S1F).

2.7 A beginners guide to implementation of the linearised form of elastic stresses and the stiffness matrix

2.7.1 Nodal definition and shape functions of a prism

For a triangular prism, the number of nodes, n, is 6, the numbering starting from the bottom three nodes (Fig. S1F). The finite element modelling discretisation using Lagrangian interpolation on a nodes can be carried through shape functions N. These shape functions and their derivatives in the parametric coordinates η, ζ and ν are given in Table 1 for a six point discretisation of the prism element. The numerical calculation for nodal forces ($\boldsymbol{g}$) and the derivatives of the forces with respect to nodal movements ($\boldsymbol{K}$) are carried out at six Gauss points, with the parametric coordinates and weights as given in Table 2.

2.7.2 Calculation of deformation gradient $\boldsymbol{F}$

The deformation gradient can be represented in the form of derivatives of current and reference coordinates in the parametric coordinates:

$$\boldsymbol{F} = \frac{\partial \boldsymbol{x}}{\partial \boldsymbol{X}} = \frac{\partial \boldsymbol{x}}{\partial \boldsymbol{\xi}} \left(\frac{\partial \boldsymbol{X}}{\partial \boldsymbol{\xi}} \right)^{-1} \tag{31}$$

By the definition of shape functions $\boldsymbol{x} = \sum_{i=1}^{n} N_i x_i$, $\partial \boldsymbol{x} / \partial \boldsymbol{\xi}$ can be obtained from the shape function derivatives with n=6 for the current prism definition and the nodal data in Table 1:

$\lambda = 1 - \xi - \eta \; \alpha = (1 - \zeta)/2 \; b = (1 + \zeta)/2$				
Node	Shape function. N	Shape function derivative wrt ξ $\frac{\partial N}{\xi}$	Shape function derivative wrt ζ $\frac{\partial N}{\zeta}$	Shape function. derivative wrt η $\frac{\partial N}{\eta}$
1	$\lambda\alpha$	$-\alpha$	$-\alpha$	$\frac{-\lambda}{2}$
2	$\xi\alpha$	α	0	$\frac{-\xi}{2}$
3	$\eta\alpha$	0	α	$\frac{-\nu}{2}$
4	λb	$-b$	$-b$	$\frac{\lambda}{2}$
5	ξb	b	0	$\frac{\xi}{2}$
6	ηb	0	b	$\frac{\eta}{2}$

Supplementary Table 1: Shape functions and derivatives of prism element. Related to STAR methods

Gauss point	ξ	ζ	η	weight
1	$\frac{1}{6}$	$\frac{1}{6}$	$\frac{1}{\sqrt{3}}$	$\frac{1}{6}$
2	$\frac{2}{3}$	$\frac{1}{6}$	$\frac{1}{\sqrt{3}}$	$\frac{1}{6}$
3	$\frac{1}{6}$	$\frac{2}{3}$	$\frac{1}{\sqrt{3}}$	$\frac{1}{6}$
4	$\frac{1}{6}$	$\frac{1}{6}$	$-\frac{1}{\sqrt{3}}$	$\frac{1}{6}$
5	$\frac{2}{3}$	$\frac{1}{6}$	$-\frac{1}{\sqrt{3}}$	$\frac{1}{6}$
6	$\frac{1}{6}$	$\frac{2}{3}$	$-\frac{1}{\sqrt{3}}$	$\frac{1}{6}$

Supplementary Table 2: Gauss points used in calculation. Related to STAR methods

$$\frac{\partial \boldsymbol{x}}{\partial \boldsymbol{\xi}} = \left(\begin{bmatrix} Shape \\ Function \\ Derivatives \end{bmatrix} \boldsymbol{x} \right)^T = \left(\begin{bmatrix} \frac{\partial N_1}{\partial \xi} & \frac{\partial N_2}{\partial \xi} & \cdots & \frac{\partial N_6}{\partial \xi} \\ \frac{\partial N_1}{\partial \eta} & \frac{\partial N_2}{\partial \eta} & \cdots & \frac{\partial N_6}{\partial \eta} \\ \frac{\partial N_1}{\partial \zeta} & \frac{\partial N_2}{\partial \zeta} & \cdots & \frac{\partial N_6}{\partial \zeta} \end{bmatrix} \begin{bmatrix} x_1 & y_1 & z_1 \\ x_2 & y_2 & z_2 \\ . & . & . \\ . & . & . \\ . & . & . \\ x_6 & y_6 & z_6 \end{bmatrix} \right)^T \tag{32}$$

and similarly,

$$\frac{\partial \boldsymbol{X}}{\partial \boldsymbol{\xi}} = \left(\begin{bmatrix} Shape \\ Function \\ Derivatives \end{bmatrix} \boldsymbol{X} \right)^T \tag{33}$$

2.7.3 Calculation of nodal elastic forces

After obtaining $\boldsymbol{F^e}$ through the calculation in Section 2.7.2, the elemental Cauchy stress can be calculated via equation (9), through the right Cauch-Green strain tensor and the second Piola-Kirshoff stress tensor $\boldsymbol{S}^e$ in (10). Once the elemental elastic stresses are obtained, these can be mapped to the nodal forces through equation (34),

$$\boldsymbol{g^e_{elemental}} = \iiint \boldsymbol{B}^T \boldsymbol{\sigma^e} |\boldsymbol{F}| \left| \frac{\partial \boldsymbol{X}}{\partial \boldsymbol{\xi}} \right| d\xi d\eta d\zeta \tag{34}$$

Here, matrix $\boldsymbol{B} = [\boldsymbol{B}_1 \dots \boldsymbol{B}_n]$ is the deformation matrix, where each matrix $\boldsymbol{B}_i$ is given by (Hughes, 2008):

$$\boldsymbol{B}_i = \begin{bmatrix} N_{i,x} & 0 & 0 \\ 0 & N_{i,y} & 0 \\ 0 & 0 & N_{i,z} \\ N_{i,y} & N_{i,x} & 0 \\ N_{i,y} & 0 & N_{i,x} \\ 0 & N_{i,z} & N_{i,x} \end{bmatrix} \tag{35}$$

and with $N_{i,x}$ being the short hand for $\partial N_i/\partial x$. Note that these derivatives are computed using the Jacobian $\partial \boldsymbol{x}/\partial \boldsymbol{\xi}$ in 32,

$$\nabla_x N_i = \left(\frac{\partial \boldsymbol{x}}{\partial \boldsymbol{\xi}} \right)^{-T} \nabla_\xi N_i$$

where $\nabla_x N_i$ is the nodal shape function derivative array as in $\nabla_x N_i = [N_{i,x}\ N_{i,y}\ N_{i,z}]^T$ and $\nabla_\xi N = [N_{i,\xi}\ N_{i,\zeta}\ N_{i,\zeta}]^T$.

2.7.4 Calculation of the stiffness matrix ($\mathbf{K}^{elast}$)

The stiffness matrix $\boldsymbol{K^{elast}}$, elastic part of the system Jacobian, is calculated in two parts, as given in the integral form in equation (11). The first part is carried out through a series of nested loops for the nodal contributions of node pair a & b. For any $\boldsymbol{K}_{ab,ik}$,

the ik component of the nodal contributions for the node pair ab of the on the element, the summation should be carried out over j,l & IJKL. The volume integration is included by the multiplication by determinants of the deformation gradient and reference shape position derivatives with respect to parametric coordinates.

$$[\mathbf{K}^e_{ab}]^1_{ik} = \iiint \sum_j \sum_l \sum_I \sum_J \sum_K \sum_L F^e_{iI} F^e_{jJ} F^e_{kK} F^e_{lL} \mathcal{C}_{IJKL} \nabla_x N^b_l \nabla_x N^a_j \, |\boldsymbol{F^g}| \left| \frac{\partial \boldsymbol{X}}{\partial \boldsymbol{\xi}} \right| d\xi d\eta d\zeta. \tag{36}$$

Here, $\nabla_x N^b$ is the nodal shape function derivative array as in $\nabla_x \boldsymbol{N}^b = [N_{b,\xi} \; N_{b,\zeta} \; N_{b,\nu}]^T$ and $\mathcal{C}$ is the Lagrangian elasticity tensor obtained from equation (12).

The second part of the integral is calculated from the $\nabla_x N^a$ and $\nabla_x N^b$ as defined above, the elemental stresses from equation (9) and the volume integration:

$$[\mathbf{K}^e_{ab}]^2_{ik} = \iiint \nabla_x N^{aT} \sigma^e \nabla_x N^b \, |\boldsymbol{F}| \left| \frac{\partial \boldsymbol{X}}{\partial \boldsymbol{\xi}} \right| d\xi d\eta d\zeta. \tag{37}$$

2.8 A pseudo algorithm of the simulation procedure

```
Initiate simulation mesh
    Set up system symmetry
    Set up tissue bounding box and relative element positions
    Set up tissue compartments (ECM, apical actomyosin layer)
    Assign tissue physical properties
    Set up boundary conditions
    Calculate shape function derivatives
    Calculate nodal masses and exposed external surfaces
    Induce mutant clones (if applicable)
Set time, t = 0.
While t < t^final:
    Reset all system forces
    Update elemental rotation matrices R_er.
    Update bounding box and relative element positions.
    Calculate F^g increments from experimental growth maps or other
    specified input (Section 2.4).
    Calculate ECM remodelling increments F^r (Section 2.5).
    Update total growth F^g of elements (Section 2.4).
    Update nodal exposed surfaces (Equation (14)).
    Detect packing nodes (Section 2.6.1).
    Update node adhesion (Section 2.6.2).
    Update elemental collapse to avoid flipping (Section 2.6.2).
    Solve for positions via implicit Newton-Raphson numerical integration:
       Initiate x_n^k = x_{n-1} for k=1.
       While the iteration displacements (δx) have not converged to zero:
          Reset matrices (g = 0 , K = I).
          Calculate nodal displacements dx(t) = x_n^k - x_{n-1}.
          Calculate elastic terms g^elast_elemental and K^elast_elemental:
             Interpolated at 6 Gauss points for each element:
                Calculate F (Section 2.7.2).
```

```
            Extract F^e (Equation (7)).
            Calculate σ (Equation (9)).
            Calculate g^{elast}_{elemental} (Section 2.7.3).
            Calculate K^{elast}_{elemental} (Section 2.7.4).
        Add each g^{elast}_{elemental} to system residual g.
        Add each K^{elast}_{elemental} to system Jacobian K.
    Calculate external viscous resistance terms:
        Calculate and add g^{ext} to system residual g (Section 2.3).
        Calculate and add K^{ext} to the system Jacobian K (Section 2.3).
    Calculate packing:
        Calculate and add packing forces to g (Section 2.6.1).
        Calculate and add packing related terms to K (Section 2.6.1 ).
    Update fixed degrees of freedom and node binding (Section 2.6.2).
    Solve for δx from g and K (Equation (6)).
    Update x_n^{k+1} = x_n^k + δx
    k = k + 1
    Check if system converged with norm(δx) < 1E-8
t = t + Δt
```

3 Analysis of simulation results

3.1 Automated fold detection and construction of apical indentation maps

Fold initiation is automatically detected by the curvature of the facing regions on the apical surface. This is done by calculating the surface normal of all elements exposed on the apical surface. If the surface normals within the vicinity of each other face in opposite directions (dot product being negative), the elements are defined to be on a folding curve. All the elements lying in between the two elements are also included on the fold surface (Fig. S1G). The threshold distance for identifying curved regions is selected as 3 μm. The threshold is selected to ensure detection of fold initiation that is clearly visible when the morphology is visualised, but it does not assign fold initiation identity to elements at opposing sides of a possible curve peak. For the looser folds of the uniform growth rate simulations, a more generous threshold of 6 μm is used. In the apical indentation maps, these automatically identified indentations are marked on the tissue outline with each continuous indentation given in a single colour (for example Fig. 3Aii).

3.2 Simulation fold position scoring

The positions of the continuous folds that are detected and that reach the midline are aligned with the experimental fold positions (Fig, 1Cii), so that the minimum deviation is calculated. If the simulation produces less than 3 folds, each missing fold is counted as 100% deviation. The deviation score is calculated as sum of percentage deviation from each experimental fold at the tissue centre, maximum total deviation being 300% The score does not penalise for additional folds, such as those observed with uniform growth of tissues with explicit BM definition (Figure 5E). The deviation score also does not check the fold morphology, such as the high hinge folds with uniform growth rates that reach taller than the pouch region (Fig. 3Ai). Therefore each low deviation value should be examined against both additional folds and non-biological fold morphology.

Parameter	Description	Value / Range	Reference
Tissue viscoelasticity			
E_{cells}	Cellular layer Young's modulus	25 Pa	Simulated for range
E_{actin}	Actin rich apical layer Young's modulus	26 - 200 Pa	Simulated for range
η_{ext}	External viscous resistance coefficient	10 - 16000 $Pa\ s\ \mu m^{-1}$	Simulated for range
ν	Poisson ratio of the tissue	0.29 $Pa\ s\ \mu m^{-1}$	Schluck et al, 2013
Basement membrane			
h_{BM}	The thickness of basement membrane	$0.2\mu m$	Calculated from measurements in this study
$t_{1/2}$	Basement membrane remodelling half-life	1 - 16 hr	Simulated for range
E_{BM}	Basement membrane Young's modulus	400 - 3200 Pa	Simulated for range
Node-node interactions			
Δ^{pack}	Packing hard-wall potential cut-off distance	40 % of local average mesh side length	selected for numerical stability selected for numerical stability
d_0	Packing hard-wall potential sigmoid shift	60 % of Δ^{pack}	selected for numerical stability
s^{sat}	Packing hard-wall potential sigmoid saturation term	5	standard logistic function
$\Delta^{collapse}$	The distance threshold below which nodes of an element are collapsed	10 % of equivalent reference shape edge length	selected for numerical stability
$\Delta^{adhesion}$	The distance threshold below which two nodes adhere	Δ^{pack}	selected for numerical stability
Tissue dimensions	The width, length and height of the tissue	see Fig. 1	Measurements in this study
Growth rates	Spatio-temporal growth rates defined by the growth maps	see Fig. 4	Measurements in this study

Supplementary Table 3: Parameters of the simulations. Related to STAR methods
